# Enhanced resting-state EEG source functional connectivity within the default mode and reward-salience networks in internet gaming disorder

**DOI:** 10.1017/S0033291722000137

**Published:** 2022-08

**Authors:** Ji-Yoon Lee, Chi-Hyun Choi, Minkyung Park, Sunyoung Park, Jung-Seok Choi

**Affiliations:** 1 Department of Psychiatry, Samsung Medical Center, Seoul, Republic of Korea; 2 Department of Psychiatry, SMG-SNU Boramae Medical Center, Seoul, Republic of Korea

**Keywords:** Default mode network, electroencephalography, internet gaming disorder, reward-salience network, source connectivity

## Abstract

**Background:**

The two key mechanisms affected by internet gaming disorder (IGD) are cognitive and reward processing. Despite their significance, little is known about neurophysiological features as determined using resting-state electroencephalography (EEG) source functional connectivity (FC).

**Methods:**

We compared resting-state EEG source FC within the default mode network (DMN) and reward/salience network (RSN) between patients with IGD and healthy controls (HCs) to identify neurophysiological markers associated with cognitive and reward processing. A total of 158 young male adults (79 patients with IGD and 79 HCs) were included, and the source FC of the DMN and RSN in five spectral bands (delta, theta, alpha, beta, and gamma) were assessed.

**Results:**

Patients with IGD showed increased theta, alpha, and beta connectivity within the DMN between the orbitofrontal cortex and parietal regions compared with HCs. In terms of RSN, patients with IGD exhibited elevated alpha and beta connectivity between the anterior cingulate gyrus and temporal regions compared with HCs. Furthermore, patients with IGD showed negative correlations between the severity of IGD symptoms and/or weekly gaming time and theta and alpha connectivity within the DMN and theta, alpha, and beta connectivity within the RSN. However, the duration of IGD was not associated with EEG source FC.

**Conclusions:**

Hyper-connectivities within the DMN and RSN may be considered potential state markers associated with symptom severity and gaming time in IGD.

## Introduction

The coronavirus disease-2019 (COVID-19) has profoundly impacted all aspects of human life globally. In particular, a marked increase in online gaming and related activities has occurred since people have been required to stay at home to protect the mselves and prevent the spread of the disease (World Health Organization, 2020). Many people struggle with social, financial, health, and pandemic-related stressors, all of which can lead to internet-related addictive behaviors (Masaeli & Farhadi, 2021). The World Health Organization (WHO) has issued a warning against excessive screen time or gaming for young people and provides guidance for limiting these activities (WHO, 2020). Internet gaming disorder (IGD) is defined by the Diagnostic and Statistical Manual of Mental Disorders, Fifth Edition (DSM-5), and the description indicates that persistent and recurrent use of internet games results in clinically significant impairment or distress and symptoms of withdrawal (American Psychiatric Association, 2013). The WHO considers gaming addiction a serious public health problem, and it was included in the International Classification of Diseases 11th Revision in 2018 (WHO, 2015, 2018). Individuals with IGD commonly have comorbid psychiatric disorders, including substance use disorders, attention-deficit hyperactivity disorders, depression, hostility, and social anxiety disorders (Ko, Yen, Yen, Chen, & Chen, 2012). Due to the rapid increase in the number of individuals diagnosed with IGD during the COVID-19 pandemic and its adverse effects, understanding the underlying mechanism is necessary for treating patients with this condition.

The two essential underlying features of IGD can be described as both cognitive and reward processing (Brand, Young, & Laier, 2014), and many studies have reported that patients with IGD display dysfunction in these abilities. Individuals with IGD showed generally lower cognitive function indices, such as full-scale intelligence quotient, verbal comprehension index, processing speed, and working memory scores compared with regular gamers and/or non-gamers (Jang et al., 2021). In an event-related potential study, the IGD group showed a higher late positive potential in response to game-related stimuli and a poor quality of decision-making compared with healthy controls (HCs; Kim et al., 2021). In a resting-state EEG study, enhanced gamma power of those with internet addiction (IA) was associated with their impaired inhibitory control (Choi et al., 2013). Higher delta and theta activity were likely associated with dysfunctional inhibitory control in those with IGD (Kim et al., 2017). With regard to reward processing, Lyvers, Karantonis, Edwards, and Thorberg (2016) suggested that reward sensitivity may be an important risk factor for IA. Another event-related potential study revealed that individuals addicted to the internet exhibited a larger P300 than HCs, which may indicate stronger reward sensitivity (He et al., 2017).

Resting-state functional connectivity (rsFC) is a measure of temporal synchrony or correlation between separate brain areas that can be used as an index of intrinsic interactions (Canuet et al., 2011; Sutherland, McHugh, Pariyadath, & Stein, 2012). In particular, this method can disclose two key features of IGD: impaired cognitive processing by the default mode network (DMN) and altered reward processing by the reward network (RN) and salience network (SN). The DMN is defined as the brain system that deactivates when people perform tasks and activates when they are at rest, and it can be used as an index to assess cognitive functions (Buckner, Andrews-Hanna, & Schacter, 2008; Dong, Li, Wang, & Potenza, 2017). RN is defined as a bias network attention toward rewarding and motivating stimuli, which are thought to underlie psychological dysfunctions associated with addiction in reward as well as affective and cognitive processing (Sutherland et al., 2012). SN is related to the hierarchical initiation of cognitive control signals and is important for identifying relevant internal and external stimuli in order to guide behavior (Li et al., 2017). Patients with IGD showed dysfunctional modulation in the DMN during functional magnetic resonance imaging (fMRI), possibly because they failed to stop playing online games regardless of the potential negative results (Wang et al., 2016). Patients with IGD exhibit marginally increased functional connectivity (FC) in the RN, which indicates that IGD enhances reward sensitivity compared with HCs (Dong, Lin, Hu, Xie, & Du, 2015). They also have increased SN-DMN connectivity, suggesting that they allocate more resources to the DMN to sustain internal mental processes against cognitive controls (Zhang et al., 2017).

Recently, however, the focus of the rsFC study has been turned into the localization of specialized brain activations to the interpretation of interrelationships in brain dynamics (Chen, Ros, & Gruzelier, 2013). Brain dynamics in the resting state reflect the electrophysiological underpinnings of human behavior and deciphering them could enhance the current understanding of basic brain function (Massar, Kenemans, & Schutter, 2014). Similar to rsFC measured with fMRI, electroencephalography (EEG) coherence, the normalized cross-power spectrum per frequency of two signals simultaneously recorded at different sites of the scalp, may serve as an expression of a person's FC (Locatelli, Cursi, Liberati, Franceschi, & Comi, 1998). Our previous study showed that individuals with IGD have excessive intrahemispheric gamma coherence, regardless of depression, anxiety, and impulsivity (Park et al., 2017). In a longitudinal coherence study, participants with IGD showed increased delta, beta, and gamma intrahemispheric coherence at baseline. Even though their IGD symptoms improved after 6 months of outpatient management, they continued to show increased beta and gamma intrahemispheric coherence compared with HCs (Park et al., 2018). In terms of network analysis, patients with IGD exhibit an enhanced theta band characteristic path length compared with HCs, and this might signify decreased efficacy of the functional network in patients with IGD (Park et al., 2020).

However, to our knowledge, no studies have investigated the underlying cognitive and reward processing using resting-state EEG source-level FC. Although rsFC usually has been measured with fMRI because of its high spatial resolution, EEG has a high temporal resolution in the millisecond range and is suitable for assessing dynamic postsynaptic activity in the cerebral cortex (Canuet et al., 2011). This study aimed to explore neurophysiological markers associated with cognitive and reward processing in IGD in the resting state via EEG source FC within the DMN and RSN. Based on previous studies, we hypothesized that patients with IGD would show hypersynchrony in the DMN and RSN compared with the HC group and that altered source FC is positively related to the severity of IGD and the amount of time spent gaming.

## Materials and methods

### Participants

The present study initially included 171 male adults aged between 18 and 40 years who were recruited from the outpatient clinic of SMG-SNU Boramae Medical Center and the local community. All participants were medication-naïve and right-handed. There were no participants with a history of psychotic or neurological disorders, significant head injury, seizure, or intellectual disability [intelligence quotient (IQ) ⩽ 80]. IGD was diagnosed by a clinically experienced psychiatrist based on the DSM-5 criteria. The Young Internet Addiction Test (IAT; Lee *et al*. 2013; Young, 1998) was used to assess the severity of IGD. All HCs were recruited from the local community and universities, none had a history of any psychiatric disorder, and all played internet games for less than 2 h per day according to previous studies (Choi et al., 2013, Son et al., 2015). The data of 13 participants were not included in the final analyses; 10 participants had EEG recordings disrupted by excessive movement or the EEG channels had poor quality (>100 *μ*V^2^), and three participants had an IQ less than 80. Thus, a total of 158 participants were included in the present analyses (79 patients with IGD and 79 HCs).

The institutional review board of SMG-SNU Boramae Medical Center approved the study protocol per the principles of the Declaration of Helsinki. All participants understood the study procedure and provided written informed consent before participation.

### Psychological assessments

#### Korean version of the young internet addiction test

The severity of IGD was assessed using the Korean version of IAT (Lee et al., 2013; Young, 1998). It includes 20 items rated using 5-point scales with a higher score reflecting a greater tendency toward IGD symptoms. The Cronbach's alpha coefficient of the IAT was 0.965.

#### Korean version of the beck depression inventory-2

The Korean version of the Beck Depression Inventory-2 (BDI) includes 21 items that measure the severity of depressive symptoms during the previous 2 weeks (Beck, Steer, & Brown, 1996; Sung et al., 2008). Items are scored on a 4-point Likert scale with higher scores indicating more severe depressive symptoms. The Cronbach's alpha coefficient for the BDI was 0.943.

#### Korean version of the beck anxiety inventory

The Korean version of the Beck Anxiety Inventory (BAI) is a 21-question assessment used to measure an individual's level of anxiety during the previous week (Beck, Epstein, Brown, & Steer, 1988; Yook & Kim, 1997). The items are scored on a 4-point Likert scale with higher scores indicating more severe anxiety symptoms. Cronbach's alpha of the BAI was 0.946.

#### Korean version of the Wechsler adult intelligence scale – fourth edition

The Korean version of the Wechsler Adult Intelligence Scale fourth edition (K-WAIS–IV; Hwang, Kim, Park, Chey, and Hong, 2012) was administered to 108 participants (55 IGD group and 53 HCs) to estimate the levels of IQ. Fifty enrolled participants (24 IGD group and 26 HCs) were assessed using the K-WAIS (Yeom, Park, Oh, Kim, & Lee, 1992). We conducted linear regression (LM) analyses of the IQ scores in each group to evaluate the differences between both K-WAIS versions in each group.

### EEG data

#### Data collection

The participants were seated in a resting position in an isolated sound-shielded room connected to the recording room via a one-way glass window. EEG was recorded for 10 min: 4 min with eyes closed, 2 min with eyes open, and 4 min with eyes closed. EEG activity was recorded using a 64-channel Quik-Cap (Compumedics Neuroscan, El Paso, TX, USA) following the modified International 10–20 system, in conjunction with recordings from vertical and horizontal electrooculograms and 1 bipolar reference electrode connected to the mastoid. All EEG recordings were obtained using SynAmps 2 (Compumedics, Abbotsford, Victoria, Australia) and the Neuroscan system (Scan 4.5; Compumedics). The electrode impedance was kept below 5 kΩ. EEG signals were amplified at a sampling rate of 1000 Hz using a 0.1–100 Hz online bandpass filter and a 0.1–50 Hz offline bandpass filter.

#### Data preprocessing

All acquired EEG data were processed using NeuroGuide software (NG; ver. 3.0.5; Applied Neuroscience, St. Petersburg, FL, USA). For the analyses, 19 of the 64 channels were selected according to a montage set with the linked-ear references from the NG as follows: FP1, FP2, F3, F7, Fz, F4, F8, C3, Cz, C4, T3, T5, T4, T6, P3, Pz, P4, O1, and O2. All EEG recordings obtained under eyes-closed conditions were selected. Artifacts were removed using the artifact rejection toolbox in the NG and were visually inspected to eliminate eye muscle movements and other artifacts. Artifact-free epochs under eyes-closed conditions were selected for the analyses.

We performed instantaneous source-level coherence analysis with a Laplacian operator using the NeuroNavigator software (NG Deluxe 3.0.5; Applied Neuroscience). The NeuroNavigator uses the structural images of the standard Collin's brain from the Montreal Neurological Institute. It uses 12 270 cortical voxel projections of the International Consortium for Brain Mapping and computes the inverse problem using standardized weighted low-resolution brain electromagnetic tomography (swLORETA; Palmero-Soler, Dolan, Hadamschek, & Tass, 2007; Park & Jung, 2020).

#### Connectivity

Based on the source-level coherence adjunct matrix, weighted and undirected networks were calculated. The nodes of the network were Brodmann area (BA) regions computed by swLORETA (Thatcher, 2010) and the edges were weighted by the coherence value within each pair of vertices. Source-level coherence between BA regions was calculated over 5 frequency bands using fast Fourier transforms: delta (1–4 Hz), theta (4–8 Hz), alpha (8–12 Hz), beta (12–30 Hz), and gamma (30–40 Hz) bands. The following were the 20 regions of interest (ROIs) for DMN from a total of 96 BAs for source-level analysis on the left and right sides: the prefrontal cortex (PFC, BA10), orbital frontal cortex (OFC, BA11), medial temporal lobe and parahippocampal gyrus (MTL&PHG, BA35), postcentral gyrus (PCG, BA2), angular gyrus and inferior parietal lobe (AG&IPL, BA39), inferior parietal lobe (IPL, BA40), posterior cingulate and superior transverse temporal gyrus (PCC&STG, BA29), posterior cingulate and cuneus (PCC&Cnu, BA30), supramarginal gyrus (SMG, BA7), and occipital cortex (OC, BA19).

RSN is anatomically defined by the NeuroNavigator software and comprises a combination of edges, with duplicated edges excluded. Twelve ROIs for the RN on the left and right sides were as follows: inferior frontal lobe (IFL, BA47), inferior frontal and extra-nuclear gyrus of the prefrontal lobes (IFL&ENG, BA44), middle frontal gyrus (MFG, BA46), superior temporal gyrus and subcallosal gyrus-entorhinal area (STG&SGTA, BA34), anterior cingulate gyrus (ACC, BA24), and insula (In, BA13). Fourteen ROIs for the SN were selected as follows on the left and right sides: PFC (BA10), temporal lobe (TL, BA22), ACC (BA24), PCC (BA23), PCC&STG (BA29), PCC&Cnu (BA30), and In (BA13).

### Statistical analyses

All statistical analyses were performed using R version 4.0.2 (R Development Core Team, Vienna, Austria). Comparisons of psychological and neurocognitive variables between groups (IGD *v.* HCs) were performed using LM. Next, a generalized linear regression model (GLM) was used to analyze the source FC in each frequency band. Data distribution was inspected via QQ-plot (R::Package ‘car’) before the analysis. We adapted a gamma GLM with a log link since all EEG data in the present study had a right-skewed distribution. Before inputting covariates into the model for group differences, collinearity was tested using the variance inflation factor (VIF) that exceed 10 indicates multicollinearity (R::Package ‘vif’). In addition, the effect size of group comparisons was calculated using the partial eta squared (*η_p_^2^*) (R::Package ‘lmsupport’).

Furthermore, we performed Spearman's correlation analyses to determine the relationships between game-related features, including weekly gaming time, IAT and the duration of IGD, and EEG source FC, which showed significant group effects in GLM analysis in patients with IGD (R::Package ‘Hmisc’). Post hoc comparisons in each analysis were corrected using the Bonferroni–Holm correction (P_BH_; Holm, 1979). The significance level for all statistical tests was set at *p* < 0.05. Visualization was performed using ggplot2 (R::Package ‘ggplot2’) and BrainNet Viewer (Xia, Wang, & He, 2013).

## Results

### Demographic and psychological assessments

The demographic and psychological characteristics of the IGD and HC groups are shown in Table 1. Age was not significantly different between groups (IGD = 24.53 ± 5.50; HCs = 25.34 ± 3.78; *p* = 0.28) whereas the HC group had more years of education than the IGD group (IGD = 13.37 ± 1.93; HC = 14.45 ± 1.76; *p* < 0.001; *η_p_^2^* = 0.08). The average onset age of IGD was 16.63 ± 5.67 years and the average duration of IGD was 8.33 ± 4.19 years. Patients with IGD spent more time gaming in a week (IGD = 6.25 ± 3.54, HC = 0.89 ± 1.12; *p* < 0.001; *η_p_^2^* = 0.51) and had a stronger severity of IGD symptoms (IGD = 66.15 ± 12.99; HC = 30.45 ± 9.02; *p* < 0.001; *η_p_^2^* = 0.72) than that of the HC group. Additionally, IGD individuals showed higher levels of depressive (IGD = 16.92 ± 9.96; HC = 3.86 ± 4.25; *p* < 0.001; *η_p_^2^* = 0.42) and anxiety symptoms (IGD = 13.49 ± 10.91, HC = 4.31 ± 5.04; *p* < 0.001; *η_p_^2^* = 0.23) and lower IQ levels (IGD = 109.81 ± 13.47; HC = 118.16 ± 10.31; *p* < 0.001; *η_p_^2^* = 0.11) than those in the HC group. BDI, BAI, and IQ were included as covariates for further EEG source FC analysis to control for depression, anxiety, and intelligence effects. The VIF values of those variables were < 10, indicating that multicollinearity was not very likely (BDI = 3.43; BAI 2.43; IQ = 1.19). In terms of K-WAIS versions, no significant differences between K-WAIS and K-WAIS–IV were found in each group (*p* = 0.121 in the IGD group; *p* = 0.075 in the HC group).

**Table 1.** Demographic and psychological characteristics between the IGD and HC groups

Variables	IGD (*n* = 79)		HCs (*n* = 79)		*t*	*p*	*η_p_^2^*
	Mean	(s.d.)	Mean	(s.d.)			
Age	24.53	(5.50)	25.34	(3.78)	−1.08	0.28	.
Education (years)	13.37	(1.93)	14.45	(1.76)	−3.64 ***	<0.001	0.08
Onset of IGD (years)	16.63	(5.67)					
Duration of IGD (years)	8.33	(4.19)					
Weekly gaming time (hours)	6.25	(3.54)	0.89	(1.12)	12.47 ***	<0.001	0.51
IAT	66.15	(12.99)	30.45	(9.02)	19.98 ***	<0.001	0.72
BDI	16.92	(9.96)	3.86	(4.25)	10.59 ***	<0.001	0.42
BAI	13.49	(10.91)	4.31	(5.04)	6.71 ***	<0.001	0.23
IQ	109.81	(13.47)	118.16	(10.31)	−4.38 ***	<0.001	0.11

IGD, Internet gaming disorder; HCs, healthy controls; s.d., standard deviation, IAT, Young's Internet addiction test; BDI, Beck Depression Inventory-2; BAI, Beck Anxiety Inventory; IQ, intelligence quotient; ***p < 0.001.

### EEG source connectivity

#### DMN

**Fig. 1.**
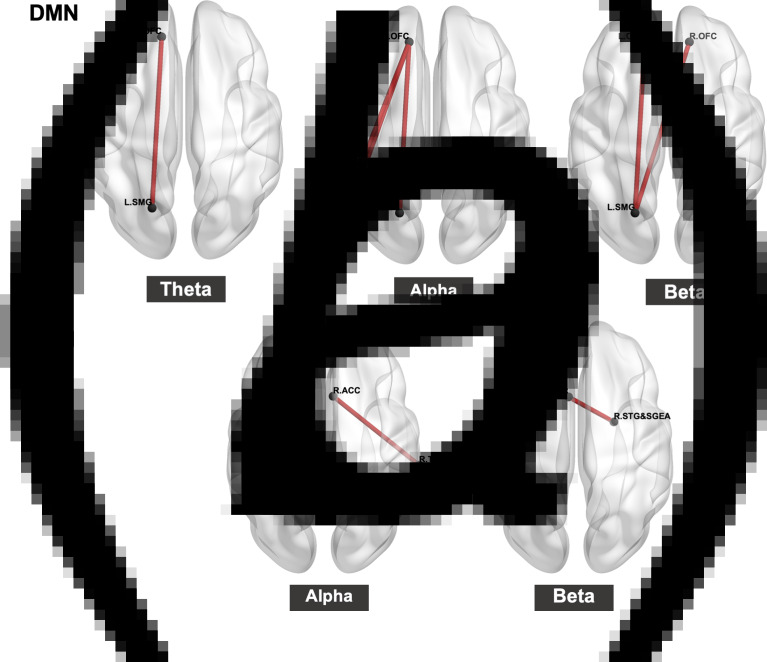
Source connectivity within (a) default mode network (DMN) and (b) reward-salience network (RSN) in each frequency band. The red line represents increased connectivity in the Internet gaming disorder (IGD) group compared with healthy controls (HCs). OFC, orbitofrontal cortex; SMG, supramarginal gyrus; IPL, inferior parietal lobe; ACC, anterior cingulate gyrus; TL, temporal lobe; STG&SGTA, superior temporal gyrus & subcallosal gyrus-entorhinal area.

The group differences for source FC within the DMN and RSN are described in Fig. 1 and online Supplementary Table S1. In the IGD group, connectivity between the left OFC and left SMG (*t* = 3.84, P_BH_ = 0.04, *η_p_^2^* = 0.76) in the DMN in the theta band was higher than that in the HC group. In the alpha band, networks between the left OFC and left PCG (*t* = 4.22, P_BH_ = 0.01, *η_p_^2^* = 0.81), left OFC and left IPL (*t* = 3.98, P_BH_ = 0.02, *η_p_^2^* = 0.79), and left OFC and left SMG (*t* = 3.84, P_BH_ = 0.03, *η_p_^2^* = 0.78) in subjects with IGD were higher than those in the HCs. In terms of DMN in the beta band, IGD had higher connectivity between the left OFC and left SMG (*t* = 4.36, P_BH_ < 0.001, *η_p_^2^* = 0.78), and right OFC and left SMG (*t* = 4.65, P_BH_ < 0.001, *η_p_^2^* = 0.81). Delta and gamma source FC within DMN were not significantly different.

#### RSN

In the RSN, patients with IGD showed increased alpha connectivity between the right TL and right ACC (*t* = 3.99, P_BH_ = 0.04, *η_p_^2^* = 0.83) compared with HCs. Furthermore, patients with IGD had increased beta activity between the left ACC and right STG&SGTA (*t* = 4.01, P_BH_ = 0.04, *η_p_^2^* = 0.96) compared with HCs. There were no significant group differences in the source-level FC within RSN in the delta, theta, and gamma bands.

### Correlation analyses

The results of the correlation analyses are presented in Table 2. In the DMN, theta source connectivity at the left OFC and left SMG of those with IGD was negatively correlated with weekly gaming time (*r* = −0.23, *p* = 0.04). There were significant negative correlations between weekly gaming time and alpha source connectivity in the left OFC and left PCG (*r* = −0.24, *p* = 0.03) and left OFC and left SMG (*r* = −0.24, *p* = 0.03). However, alpha source connectivity in the left OFC and left IPL was not correlated with IAT (*r* = −0.17, *p* = 0.14) or weekly gaming time (*r* = −0.22, *p* = 0.05). In the beta band, weekly gaming time was negatively correlated with each network at the right OFC and left SMG (*r* = −0.27, *p* = 0.02), and at the right OFC and left SMG (*r* = −0.28, *p* = 0.01). Regarding the duration of IGD, there were no significant correlations with theta, alpha, and beta connectivity. Compared with individuals who had IGD, HCs had no significant correlations between EEG source FC and weekly gaming time or IAT.

**Table 2.** Correlation between EEG source connectivity, IAT, weekly gaming time, and duration of IGD

Network /Frequency Band	Nodes	IGD						HCs			
		IAT		weekly gaming time		Duration of IGD		IAT		weekly gaming time	
		*r*	*p*	*r*	*p*	*r*	*p*	*r*	*p*	*r*	*p*
DMN												
Theta	left OFC	left SMG	−0.20	0.08	−0.23*	0.04	0.15	0.43	−0.09	0.43	0.03	0.78
Alpha	left OFC	left PCG	−0.21	0.07	−0.24*	0.03	0.22	0.25	0.05	0.69	−0.05	0.67
	left OFC	left IPL	−0.17	0.14	−0.22	0.05	0.24	0.20	0.02	0.87	−0.09	0.46
	left OFC	left SMG	−0.15	0.18	−0.24*	0.03	0.21	0.26	−0.01	0.94	−0.03	0.78
Beta	left OFC	left SMG	−0.21	0.06	−0.25*	0.03	0.21	0.26	0.03	0.80	0.06	0.64
	right OFC	left SMG	−0.19	0.10	−0.28*	0.01	0.21	0.27	0.10	0.36	0.13	0.28
RSN												
Alpha	right ACC	right TL	−0.27*	0.02	−0.27*	0.02	0.23	0.22	0.11	0.33	−0.06	0.64
Beta	left ACC	right STG&SGTA	−0.23*	0.04	−0.13	0.24	0.26	0.17	−0.03	0.79	0.00	0.99

EEG, electroencephalography; IAT, Young's Internet Addiction Test; weekly gaming time, weekly time spent gaming per hour; IGD, internet gaming disorder, HCs, health controls; *r*, correlation coefficient; DMN, default mode network; RSN, reward-salience network; OFC, orbitofrontal cortex; SMG, supramarginal gyrus; PCG, post central gyrus; TL, temporal lobe; ACC, anterior cingulate gyrus; STG&SGTA, superior temporal gyrus & subcallosal gyrus-entorhinal area; *p < 0.05.

**Fig. 2.**
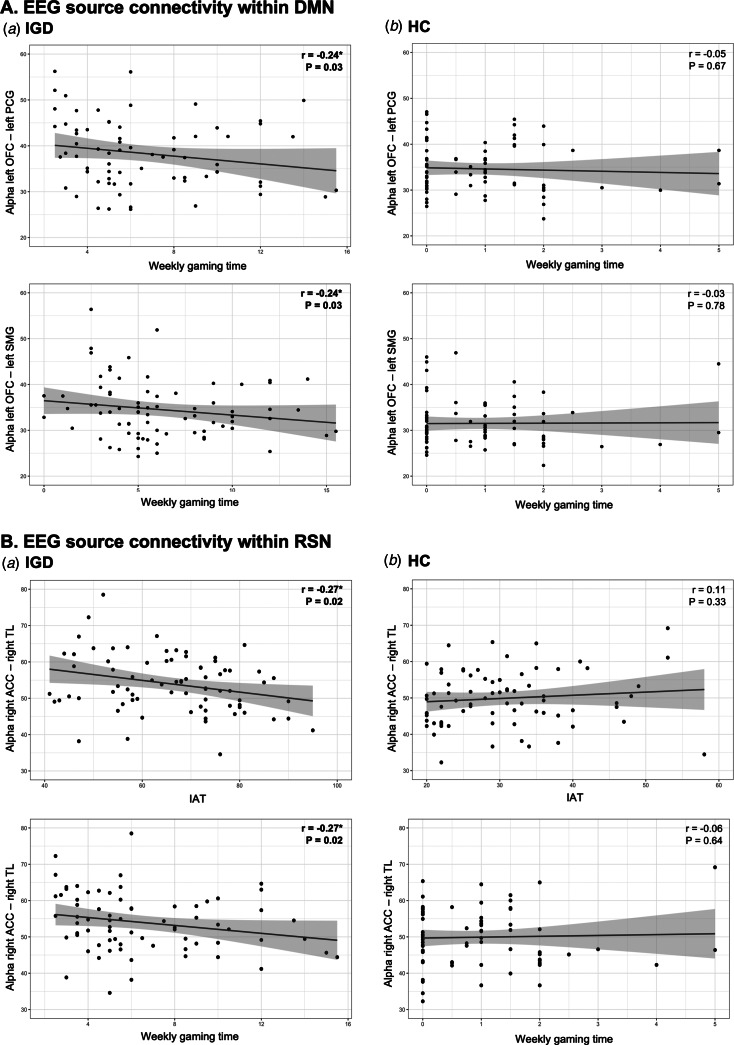
Correlations in the Internet gaming disorder (IGD) and healthy control (HC) groups. **A.** EEG source connectivity within default mode network (DMN). (a) Negative correlations between weekly gaming time (hours) and alpha source connectivity in the left orbitofrontal cortex (OFC) and left postcentral gyrus (PCG; upper) and left OFC and left supramarginal gyrus (SMG; lower) in the IGD group. (b) There was no significant correlation between weekly gaming time and alpha source connectivity in the left OFC and left PCG (upper) and left OFC and left SMG (lower) in the HC group. **B.** EEG source connectivity within a reward-salience network (RSN). (a) Negative correlations between alpha source connectivity at the anterior cingulate gyrus (ACC) and right temporal lobe (TL) and scores on Young's Internet Addiction Test (IAT; upper) weekly gaming time (lower) in the IGD group. (b) There was no significant correlation between alpha source connectivity at the ACC and TL and scores on IAT (upper) weekly gaming time (lower) in the HC group.

In the RSN, patients with IGD showed negative correlations between alpha source connectivity at the right ACC and right TL and IAT (*r* = −0.27, *p* = 0.02) and weekly gaming time (*r* = −0.27, *p* = 0.02). Furthermore, beta source connectivity at the left ACC and right STG&SGTA was negatively correlated with IAT (*r* = −0.23, *p* = 0.04). However, the duration of IGD was not correlated with alpha and beta connectivity. Along with DMN, there were no significant correlations in the HC group. Correlations with alpha source connectivity within DMN and RSN are presented in Fig. 2.

## Discussion

The present study aimed to identify alterations in cognitive and reward processing in individuals with IGD in the resting state via EEG source connectivity within the DMN and RSN. In the DMN, the IGD group exhibited elevated theta FC at the OFC and left SMG and increased alpha FC at the left/right OFC and left PCG, left IPL or left SMG, and increased beta FC at the left OFC and left/right SMG compared with the HC group. Regarding the RSN, patients with IGD showed increased alpha FC at the left/right ACC and right TL, and elevated beta FC at the left ACC and right STG and STG&SGTA compared with the HC group. In the correlation analysis, patients with IGD had a negative correlation between theta, alpha, and beta source FC within the DMN at the left/right OFC and left SMG or left PCG and weekly gaming time. In the RSN, alpha and beta bands functional source connectivity of IGD at the left/right ACC and right TL, and right STG&SGTA were correlated with the severity of IGD symptoms and/or weekly gaming time. The duration of IGD, on the other hand, was not related to the EEG source FC.

The strong links between the frontal and parietal regions within the DMN suggest that individuals with IGD have impairments in resting-state FC associated with cognitive domains compared with the HCs. Prefrontal lobe function contributes to various cognitive processes, including memory, inhibitory and attentional control, and behavioral monitoring (Hu et al., 2012). Previous research has found associations between the frontal lobe and cognitive functioning in the IGD. Those addicted to gaming have poorer response inhibition, working memory, decision-making, and emotion regulation, which is associated with reduced prefrontal cortex functioning (Kuss, Pontes, & Griffiths, 2018). Recent meta-analysis studies have found that people with problematic internet usage (PIU) have cognitive impairments including motor inhibitory control, working memory, and decision-making and decreased gray matter in the dorsolateral prefrontal cortex and anterior cingulate cortex, which are implicated in reward processing, decision making and top-down inhibitory control (Ioannidis et al., 2019; Solly, Hook, Grant, Cortese, & Chamberlain, 2021). Increased EEG FC in the alpha and beta frequency bands within the right hemisphere may be involved in the recurrent activation of visuospatial working memory and executive function due to excessive gaming (Burleigh, Griffiths, Sumich, Wang, & Kuss, 2020). Therefore, excessive online gaming has the possibility to impair cognitive processes, such as executive function, inhibitory control, working memory, and risk decision-making. This may be reflected in dysfunctional brain activity at rest and increased theta, alpha, and beta source connectivity in the frontoparietal cortex.

As we hypothesized, the present study showed that those with IGD had enhanced source connectivity with the ACC when their brains were at rest despite controlling emotional states, including depressive and anxious mood. The ACC, in particular, evaluates the consequences of actions and whether reward or punishment has been received, and utilizes the information to learn the action to obtain a reward or avoid punishment (Rolls, 2019). Furthermore, it is associated with emotion regulation, since it links reward and punishment information that generates emotional responses to behavior (Fitzgerald, Klumpp, Langenecker, & Phan, 2019). The ventral ACC is responsible for regulating negative emotion, whereas the dorsal ACC is responsible for response selection and interference control (Zhang et al., 2020). The ACC may be activated to evaluate the reward significance and be implicated in determining the degree of desire for online gaming, according to an event-related fMRI study (Ko et al., 2013). The over-activation in the dorsal ACC of participants with IGD suggests that they need to engage more effort and resources to downregulate their negative emotions than recreational game users, resulting in a higher cognitive load (Zhang et al., 2020). In terms of EEG oscillations, the alpha band plays an important role in different memory processes and emotion regulation (Imperatori et al., 2014). Taken together, the hyperactivations may be attributed to the difficulty in evaluating the consequences of actions when patients with IGD receive a reward or punishment and lack of control for emotional regulation of their actions. Therefore, they continue internet gaming even if they receive negative feedback for their actions. Our findings are restricted, however, because the ACC was not separated into dorsal and ventral regions, and further studies are needed to establish the specific emotion regulation functions of the ACC.

In terms of beta source connectivity in both DMN and RSN, our previous longitudinal study revealed that increased beta coherence could be a neurobiological marker for the pathophysiology of IGD (Park et al., 2018). Therefore, beta source connectivity between the frontoparietal regions and the frontotemporal regions can also be suggested as a neurobiological marker for the mechanism of IGD.

Interestingly, theta, alpha, and beta source connectivities within the DMN and RSN exhibited negative associations with IAT and weekly gaming time but there was no significant relationship with the duration of IGD in the current study. This, however, was contrary to our hypothesis. Patients with IGD who had more severe IGD symptoms and spent more time playing games exhibited lowered EEG source connectivity. In case of substance abuse disorders, prolonged exposure to the dopamine-enhancing effects of most drugs resulted in neuroadaptations of the reward system (Volkow, Koob, & McLellan, 2016). According to Kuss and Griffiths (2012), IA appears to enhance brain activity in the same way as other behavioral addictions do. This may lead to neuroadaptation, in which the brain is altered as a result of excessive internet and game use. However, our findings were restricted in terms of cross-sectional design, and further longitudinal studies are needed to clarify a causal relationship between EEG source FC and IGD symptoms or gaming activity.

The present study had several limitations. First, only male participants were included. Second, the sample size was insufficient to be considered representative of all patients with IGD, and because these issues may limit the generalizability of the results, further investigations with larger sample sizes and including female participants are necessary. Third, there is a lack of evaluation of psychopathological symptoms and mental health comorbidities, which are prominent in IGD and may influence its neurocognitive connections. Although an expert psychiatrist conducted a clinical interview with the IGD group, it is difficult to control for the possibility that present results were influenced by the confounding effects of other psychopathological symptoms. Forth, the results of the present study are limited since it uses only 19-channel derived from 64 channels. We cannot completely neglect the bias of employing a small number of electrodes, including mislocalization and/or blurring for source connectivity analysis. Therefore, further research with a large number of electrodes should be conducted to clarify EEG source FC related with IGD. Fifth, the causal effects between the EEG source FC and psychological variables were insufficient. Further studies attending mediating or moderating effects are required.

Despite these limitations, the present study revealed dysfunctional cognitive and reward processes of IGD in the resting state via EEG source FC within the DMN and RSN. Enhanced EEG source connectivities within the DMN and RSN, in particular, may be considered potential state markers associated with symptom severity and gaming time in IGD. Our findings provide a greater understanding of the underlying neural characteristics in IGD to help discover possible targets for further treatment of IGD.

## Supplementary material

For supplementary material accompanying this paper visit https://doi.org/10.1017/S0033291722000137. click here to view supplementary material
