# Supplementary material for: Enhanced resting-state EEG source functional connectivity within the default mode and reward-salience networks in internet gaming disorder
Source: Psychol Med. 2022 Feb 23;52(11):2189–97. doi: 10.1017/S0033291722000137 (PMC9386438; doi:10.1017/S0033291722000137)
Supplement: Supplementary file 1 [file S0033291722000137sup001.docx]

**Supplementary table S1.** Group comparisons of EEG source connectivity within default mode network between IGD and HC groups

| **Node** | | **Delta** | | | **Theta** | | | **Alpha** | | | **Beta** | | | **Gamma** | | |
| --- | --- | --- | --- | --- | --- | --- | --- | --- | --- | --- | --- | --- | --- | --- | --- | --- |
|  |  | **t** | **P** | **P_BH_** | **t** | **P** | **P_BH_** | **t** | **P** | **P_BH_** | **t** | **P** | **P_BH_** | **t** | **P** | **P_BH_** |
| Left Pre-Frontal Cortex | Right Pre-Frontal Cortex | -0.28 | 0.78 | >.999 | -0.67 | 0.50 | >.999 | -1.01 | 0.31 | >.999 | -0.75 | 0.46 | >.999 | -0.12 | 0.90 | >.999 |
| Left Pre-Frontal Cortex | Left Orbital Frontal | -0.63 | 0.53 | >.999 | -0.94 | 0.35 | >.999 | -0.53 | 0.59 | >.999 | -1.34 | 0.18 | >.999 | -1.07 | 0.28 | >.999 |
| Left Pre-Frontal Cortex | Right Orbital Frontal | 0.45 | 0.65 | >.999 | 0.27 | 0.79 | >.999 | 0.55 | 0.58 | >.999 | 0.22 | 0.83 | >.999 | 0.29 | 0.77 | >.999 |
| Left Pre-Frontal Cortex | Left Occipital Cortex | 0.67 | 0.50 | >.999 | 1.68 | 0.09 | >.999 | 2.82 | 0.01 | 0.73 | 1.05 | 0.29 | >.999 | 0.06 | 0.95 | >.999 |
| Left Pre-Frontal Cortex | Right Occipital Cortex | 0.26 | 0.79 | >.999 | 1.39 | 0.17 | >.999 | 2.55 | 0.01 | >.999 | 0.36 | 0.72 | >.999 | -1.05 | 0.29 | >.999 |
| Left Pre-Frontal Cortex | Left Posterior Cingulate & Superior Transverse Temporal Gyrus | 2.29 | 0.02 | >.999 | 2.16 | 0.03 | >.999 | 2.92 | <.001 | 0.59 | 2.64 | 0.01 | >.999 | 1.82 | 0.07 | >.999 |
| Left Pre-Frontal Cortex | Right Posterior Cingulate & Superior Transverse Temporal Gyrus | 2.18 | 0.03 | >.999 | 1.77 | 0.08 | >.999 | 2.68 | 0.01 | 0.95 | 2.08 | 0.04 | >.999 | 1.19 | 0.24 | >.999 |
| Left Pre-Frontal Cortex | Left Posterior Cingulate & Cuneus | 1.65 | 0.10 | >.999 | 1.39 | 0.17 | >.999 | 2.27 | 0.02 | >.999 | 1.95 | 0.05 | >.999 | 1.46 | 0.15 | >.999 |
| Left Pre-Frontal Cortex | Right Posterior Cingulate & Cuneus | 1.74 | 0.08 | >.999 | 1.27 | 0.21 | >.999 | 2.14 | 0.03 | >.999 | 1.77 | 0.08 | >.999 | 1.09 | 0.28 | >.999 |
| Left Pre-Frontal Cortex | Left Medial Temporal Lobe & Parahippocampal Gyrus | 1.43 | 0.15 | >.999 | 1.26 | 0.21 | >.999 | 2.24 | 0.03 | >.999 | 2.20 | 0.03 | >.999 | 1.74 | 0.08 | >.999 |
| Left Pre-Frontal Cortex | Right Medial Temporal Lobe & Parahippocampal Gyrus | 1.60 | 0.11 | >.999 | 1.26 | 0.21 | >.999 | 1.85 | 0.07 | >.999 | 2.33 | 0.02 | >.999 | 2.03 | 0.04 | >.999 |
| Left Pre-Frontal Cortex | Left Angular Gyrus & Inferior Parietal Lobe | 1.89 | 0.06 | >.999 | 2.43 | 0.02 | >.999 | 2.67 | 0.01 | 0.96 | 1.65 | 0.10 | >.999 | 0.81 | 0.42 | >.999 |
| Left Pre-Frontal Cortex | Right Angular Gyrus & Inferior Parietal Lobe | 0.95 | 0.34 | >.999 | 1.63 | 0.11 | >.999 | 2.80 | 0.01 | 0.76 | 1.17 | 0.24 | >.999 | -0.74 | 0.46 | >.999 |
| Left Pre-Frontal Cortex | Left Inferior Parietal Lobe Angular Gyrus | 1.83 | 0.07 | >.999 | 2.34 | 0.02 | >.999 | 3.13 | <.001 | 0.34 | 1.68 | 0.10 | >.999 | 0.38 | 0.70 | >.999 |
| Left Pre-Frontal Cortex | Right Inferior Parietal Lobe Angular Gyrus | 1.46 | 0.15 | >.999 | 1.75 | 0.08 | >.999 | 2.73 | 0.01 | 0.84 | 1.73 | 0.09 | >.999 | 0.90 | 0.37 | >.999 |
| Right Pre-Frontal Cortex | Left Orbital Frontal | 0.08 | 0.93 | >.999 | -0.48 | 0.63 | >.999 | -0.38 | 0.71 | >.999 | 0.39 | 0.69 | >.999 | 1.38 | 0.17 | >.999 |
| Right Pre-Frontal Cortex | Right Orbital Frontal | -1.13 | 0.26 | >.999 | -1.78 | 0.08 | >.999 | -1.95 | 0.05 | >.999 | -1.83 | 0.07 | >.999 | ->.999 | 0.32 | >.999 |
| Right Pre-Frontal Cortex | Left Occipital Cortex | 0.60 | 0.55 | >.999 | 1.48 | 0.14 | >.999 | 2.29 | 0.02 | >.999 | 2.24 | 0.03 | >.999 | 1.65 | 0.10 | >.999 |
| Right Pre-Frontal Cortex | Right Occipital Cortex | 0.50 | 0.62 | >.999 | 1.16 | 0.25 | >.999 | 2.58 | 0.01 | >.999 | 2.77 | 0.01 | >.999 | 1.30 | 0.20 | >.999 |
| Right Pre-Frontal Cortex | Left Posterior Cingulate & Superior Transverse Temporal Gyrus | 1.26 | 0.21 | >.999 | 1.28 | 0.20 | >.999 | 2.17 | 0.03 | >.999 | 2.59 | 0.01 | >.999 | 1.90 | 0.06 | >.999 |
| Right Pre-Frontal Cortex | Right Posterior Cingulate & Superior Transverse Temporal Gyrus | 0.67 | 0.51 | >.999 | 0.90 | 0.37 | >.999 | 1.92 | 0.06 | >.999 | 1.64 | 0.10 | >.999 | 0.74 | 0.46 | >.999 |
| Right Pre-Frontal Cortex | Left Posterior Cingulate & Cuneus | 1.44 | 0.15 | >.999 | 0.83 | 0.41 | >.999 | 1.34 | 0.18 | >.999 | 1.47 | 0.14 | >.999 | 1.17 | 0.24 | >.999 |
| Right Pre-Frontal Cortex | Right Posterior Cingulate & Cuneus | 0.38 | 0.70 | >.999 | 0.40 | 0.69 | >.999 | 1.11 | 0.27 | >.999 | 0.81 | 0.42 | >.999 | 0.37 | 0.72 | >.999 |
| Right Pre-Frontal Cortex | Left Medial Temporal Lobe & Parahippocampal Gyrus | 1.64 | 0.10 | >.999 | 0.91 | 0.37 | >.999 | 1.30 | 0.20 | >.999 | 1.66 | 0.10 | >.999 | 1.53 | 0.13 | >.999 |
| Right Pre-Frontal Cortex | Right Medial Temporal Lobe & Parahippocampal Gyrus | 0.66 | 0.51 | >.999 | 0.32 | 0.75 | >.999 | 0.60 | 0.55 | >.999 | 1.00 | 0.32 | >.999 | 0.99 | 0.32 | >.999 |
| Right Pre-Frontal Cortex | Left Angular Gyrus & Inferior Parietal Lobe | 2.04 | 0.04 | >.999 | 2.30 | 0.02 | >.999 | 2.50 | 0.01 | >.999 | 1.51 | 0.13 | >.999 | 0.47 | 0.64 | >.999 |
| Right Pre-Frontal Cortex | Right Angular Gyrus & Inferior Parietal Lobe | 0.28 | 0.78 | >.999 | 1.59 | 0.11 | >.999 | 2.78 | 0.01 | 0.80 | 1.97 | 0.05 | >.999 | 0.55 | 0.58 | >.999 |
| Right Pre-Frontal Cortex | Left Inferior Parietal Lobe Angular Gyrus | 1.91 | 0.06 | >.999 | 2.10 | 0.04 | >.999 | 2.78 | 0.01 | 0.79 | 1.17 | 0.24 | >.999 | 0.15 | 0.88 | >.999 |
| Right Pre-Frontal Cortex | Right Inferior Parietal Lobe Angular Gyrus | 2.06 | 0.04 | >.999 | 2.79 | 0.01 | >.999 | 3.05 | <.001 | 0.42 | 1.96 | 0.05 | >.999 | 0.88 | 0.38 | >.999 |
| Left Orbital Frontal | Right Orbital Frontal | 1.70 | 0.09 | >.999 | 1.38 | 0.17 | >.999 | 1.18 | 0.24 | >.999 | 3.17 | <.001 | 0.34 | 3.38 | <.001 | 0.18 |
| Left Orbital Frontal | Left Occipital Cortex | 1.48 | 0.14 | >.999 | 1.69 | 0.09 | >.999 | 2.47 | 0.01 | >.999 | 1.50 | 0.14 | >.999 | 0.50 | 0.62 | >.999 |
| Left Orbital Frontal | Right Occipital Cortex | 0.86 | 0.39 | >.999 | 1.79 | 0.08 | >.999 | 2.69 | 0.01 | 0.95 | 1.47 | 0.14 | >.999 | 0.37 | 0.71 | >.999 |
| Left Orbital Frontal | Left Posterior Cingulate & Superior Transverse Temporal Gyrus | 2.87 | <.001 | 0.87 | 3.18 | <.001 | 0.33 | 3.52 | <.001 | 0.11 | 3.70 | <.001 | 0.06 | 2.64 | 0.01 | >.999 |
| Left Orbital Frontal | Right Posterior Cingulate & Superior Transverse Temporal Gyrus | 2.62 | 0.01 | >.999 | 2.98 | <.001 | 0.61 | 3.25 | <.001 | 0.25 | 3.00 | <.001 | 0.58 | 1.63 | 0.10 | >.999 |
| Left Orbital Frontal | Left Posterior Cingulate & Cuneus | 2.06 | 0.04 | >.999 | 2.28 | 0.02 | >.999 | 2.86 | <.001 | 0.68 | 1.97 | 0.05 | >.999 | 0.90 | 0.37 | >.999 |
| Left Orbital Frontal | Right Posterior Cingulate & Cuneus | 2.06 | 0.04 | >.999 | 2.36 | 0.02 | >.999 | 2.75 | 0.01 | 0.83 | 1.91 | 0.06 | >.999 | 0.63 | 0.53 | >.999 |
| Left Orbital Frontal | Left Medial Temporal Lobe & Parahippocampal Gyrus | 2.46 | 0.02 | >.999 | 2.75 | 0.01 | >.999 | 3.21 | <.001 | 0.27 | 2.67 | 0.01 | >.999 | 1.68 | 0.10 | >.999 |
| Left Orbital Frontal | Right Medial Temporal Lobe & Parahippocampal Gyrus | 2.78 | 0.01 | >.999 | 2.99 | <.001 | 0.59 | 3.13 | <.001 | 0.34 | 2.81 | 0.01 | 0.99 | 1.73 | 0.09 | >.999 |
| Left Orbital Frontal | Left Angular Gyrus & Inferior Parietal Lobe | 2.63 | 0.01 | >.999 | 2.76 | 0.01 | >.999 | 3.22 | <.001 | 0.27 | 1.68 | 0.09 | >.999 | 0.15 | 0.88 | >.999 |
| Left Orbital Frontal | Right Angular Gyrus & Inferior Parietal Lobe | 1.43 | 0.16 | >.999 | 2.39 | 0.02 | >.999 | 3.13 | <.001 | 0.34 | 0.92 | 0.36 | >.999 | -0.95 | 0.35 | >.999 |
| Left Orbital Frontal | Left Inferior Parietal Lobe Angular Gyrus | 3.42 | <.001 | 0.15 | 2.94 | <.001 | 0.68 | 3.98* | <.001 | 0.02 | 2.35 | 0.02 | >.999 | -0.23 | 0.82 | >.999 |
| Left Orbital Frontal | Right Inferior Parietal Lobe Angular Gyrus | 1.91 | 0.06 | >.999 | 2.56 | 0.01 | >.999 | 3.24 | <.001 | 0.26 | 2.09 | 0.04 | >.999 | 0.58 | 0.56 | >.999 |
| Right Orbital Frontal | Left Occipital Cortex | 1.60 | 0.11 | >.999 | 1.95 | 0.05 | >.999 | 2.63 | 0.01 | >.999 | 2.04 | 0.04 | >.999 | 1.19 | 0.24 | >.999 |
| Right Orbital Frontal | Right Occipital Cortex | 0.57 | 0.57 | >.999 | 1.43 | 0.15 | >.999 | 2.61 | 0.01 | >.999 | 2.09 | 0.04 | >.999 | 1.21 | 0.23 | >.999 |
| Right Orbital Frontal | Left Posterior Cingulate & Superior Transverse Temporal Gyrus | 2.53 | 0.01 | >.999 | 2.98 | <.001 | 0.60 | 3.31 | <.001 | 0.21 | 3.61 | <.001 | 0.08 | 2.82 | 0.01 | >.999 |
| Right Orbital Frontal | Right Posterior Cingulate & Superior Transverse Temporal Gyrus | 2.05 | 0.04 | >.999 | 2.61 | 0.01 | >.999 | 3.01 | <.001 | 0.47 | 2.78 | 0.01 | >.999 | 1.60 | 0.11 | >.999 |
| Right Orbital Frontal | Left Posterior Cingulate & Cuneus | 2.07 | 0.04 | >.999 | 2.34 | 0.02 | >.999 | 2.79 | 0.01 | 0.77 | 2.24 | 0.03 | >.999 | 1.42 | 0.16 | >.999 |
| Right Orbital Frontal | Right Posterior Cingulate & Cuneus | 1.33 | 0.18 | >.999 | 1.87 | 0.06 | >.999 | 2.51 | 0.01 | >.999 | 1.74 | 0.08 | >.999 | 0.76 | 0.45 | >.999 |
| Right Orbital Frontal | Left Medial Temporal Lobe & Parahippocampal Gyrus | 2.47 | 0.01 | >.999 | 2.66 | 0.01 | >.999 | 3.12 | <.001 | 0.35 | 2.87 | <.001 | 0.84 | 2.24 | 0.03 | >.999 |
| Right Orbital Frontal | Right Medial Temporal Lobe & Parahippocampal Gyrus | 1.61 | 0.11 | >.999 | 2.15 | 0.03 | >.999 | 2.48 | 0.01 | >.999 | 2.17 | 0.03 | >.999 | 1.51 | 0.13 | >.999 |
| Right Orbital Frontal | Left Angular Gyrus & Inferior Parietal Lobe | 2.70 | 0.01 | >.999 | 2.81 | 0.01 | 0.98 | 3.36 | <.001 | 0.18 | 2.06 | 0.04 | >.999 | 0.62 | 0.54 | >.999 |
| Right Orbital Frontal | Right Angular Gyrus & Inferior Parietal Lobe | 0.79 | 0.43 | >.999 | 2.01 | 0.05 | >.999 | 2.96 | <.001 | 0.52 | 1.76 | 0.08 | >.999 | 0.07 | 0.94 | >.999 |
| Right Orbital Frontal | Left Inferior Parietal Lobe Angular Gyrus | 3.08 | <.001 | 0.45 | 2.58 | 0.01 | >.999 | 3.66 | <.001 | 0.06 | 2.54 | 0.01 | >.999 | 0.43 | 0.67 | >.999 |
| Right Orbital Frontal | Right Inferior Parietal Lobe Angular Gyrus | 1.78 | 0.08 | >.999 | 2.61 | 0.01 | >.999 | 3.24 | <.001 | 0.26 | 2.44 | 0.02 | >.999 | 1.04 | 0.30 | >.999 |
| Left Occipital Cortex | Right Occipital Cortex | 1.09 | 0.28 | >.999 | 1.26 | 0.21 | >.999 | 1.67 | 0.10 | >.999 | 1.71 | 0.09 | >.999 | 2.01 | 0.05 | >.999 |
| Left Occipital Cortex | Left Posterior Cingulate & Superior Transverse Temporal Gyrus | 0.80 | 0.43 | >.999 | 0.63 | 0.53 | >.999 | 0.87 | 0.39 | >.999 | 1.06 | 0.29 | >.999 | 1.08 | 0.28 | >.999 |
| Left Occipital Cortex | Right Posterior Cingulate & Superior Transverse Temporal Gyrus | 1.02 | 0.31 | >.999 | 0.72 | 0.47 | >.999 | 1.21 | 0.23 | >.999 | 2.09 | 0.04 | >.999 | 2.24 | 0.03 | >.999 |
| Left Occipital Cortex | Left Posterior Cingulate & Cuneus | 0.84 | 0.40 | >.999 | 0.81 | 0.42 | >.999 | 1.17 | 0.24 | >.999 | 1.47 | 0.14 | >.999 | 1.84 | 0.07 | >.999 |
| Left Occipital Cortex | Right Posterior Cingulate & Cuneus | 1.26 | 0.21 | >.999 | 1.31 | 0.19 | >.999 | 1.91 | 0.06 | >.999 | 2.62 | 0.01 | >.999 | 2.54 | 0.01 | >.999 |
| Left Occipital Cortex | Left Medial Temporal Lobe & Parahippocampal Gyrus | 1.06 | 0.29 | >.999 | 0.65 | 0.52 | >.999 | 1.11 | 0.27 | >.999 | 1.83 | 0.07 | >.999 | 2.29 | 0.02 | >.999 |
| Left Occipital Cortex | Right Medial Temporal Lobe & Parahippocampal Gyrus | 1.35 | 0.18 | >.999 | 1.47 | 0.14 | >.999 | 2.00 | 0.05 | >.999 | 2.41 | 0.02 | >.999 | 2.16 | 0.03 | >.999 |
| Left Occipital Cortex | Left Angular Gyrus & Inferior Parietal Lobe | 0.63 | 0.53 | >.999 | 0.45 | 0.65 | >.999 | 0.77 | 0.44 | >.999 | -0.01 | 0.99 | >.999 | -0.37 | 0.71 | >.999 |
| Left Occipital Cortex | Right Angular Gyrus & Inferior Parietal Lobe | 0.06 | 0.95 | >.999 | 1.17 | 0.24 | >.999 | 2.31 | 0.02 | >.999 | 0.48 | 0.63 | >.999 | -0.30 | 0.76 | >.999 |
| Left Occipital Cortex | Left Inferior Parietal Lobe Angular Gyrus | 1.79 | 0.08 | >.999 | 1.30 | 0.20 | >.999 | 1.39 | 0.17 | >.999 | 0.27 | 0.78 | >.999 | -0.59 | 0.55 | >.999 |
| Left Occipital Cortex | Right Inferior Parietal Lobe Angular Gyrus | 0.89 | 0.38 | >.999 | 1.43 | 0.15 | >.999 | 2.71 | 0.01 | 0.90 | 2.05 | 0.04 | >.999 | 1.07 | 0.29 | >.999 |
| Right Occipital Cortex | Left Posterior Cingulate & Superior Transverse Temporal Gyrus | 1.81 | 0.07 | >.999 | 1.50 | 0.14 | >.999 | 2.17 | 0.03 | >.999 | 2.42 | 0.02 | >.999 | 1.37 | 0.17 | >.999 |
| Right Occipital Cortex | Right Posterior Cingulate & Superior Transverse Temporal Gyrus | 2.55 | 0.01 | >.999 | 1.76 | 0.08 | >.999 | 2.08 | 0.04 | >.999 | 2.80 | 0.01 | >.999 | 1.65 | 0.10 | >.999 |
| Right Occipital Cortex | Left Posterior Cingulate & Cuneus | 1.42 | 0.16 | >.999 | 1.43 | 0.16 | >.999 | 2.26 | 0.03 | >.999 | 1.90 | 0.06 | >.999 | 0.95 | 0.34 | >.999 |
| Right Occipital Cortex | Right Posterior Cingulate & Cuneus | 1.55 | 0.12 | >.999 | 1.19 | 0.24 | >.999 | 1.65 | 0.10 | >.999 | 1.81 | 0.07 | >.999 | 1.28 | 0.20 | >.999 |
| Right Occipital Cortex | Left Medial Temporal Lobe & Parahippocampal Gyrus | 1.59 | 0.11 | >.999 | 1.74 | 0.08 | >.999 | 2.52 | 0.01 | >.999 | 1.56 | 0.12 | >.999 | 0.72 | 0.47 | >.999 |
| Right Occipital Cortex | Right Medial Temporal Lobe & Parahippocampal Gyrus | 1.26 | 0.21 | >.999 | 1.02 | 0.31 | >.999 | 1.72 | 0.09 | >.999 | 1.33 | 0.18 | >.999 | 0.76 | 0.45 | >.999 |
| Right Occipital Cortex | Left Angular Gyrus & Inferior Parietal Lobe | 0.84 | 0.40 | >.999 | 2.13 | 0.03 | >.999 | 2.85 | 0.01 | 0.69 | 0.62 | 0.54 | >.999 | -0.25 | 0.80 | >.999 |
| Right Occipital Cortex | Right Angular Gyrus & Inferior Parietal Lobe | 2.74 | 0.01 | >.999 | 1.88 | 0.06 | >.999 | 2.54 | 0.01 | >.999 | 2.18 | 0.03 | >.999 | 0.96 | 0.34 | >.999 |
| Right Occipital Cortex | Left Inferior Parietal Lobe Angular Gyrus | 2.22 | 0.03 | >.999 | 2.93 | <.001 | 0.69 | 3.27 | <.001 | 0.24 | 1.59 | 0.11 | >.999 | -0.31 | 0.75 | >.999 |
| Right Occipital Cortex | Right Inferior Parietal Lobe Angular Gyrus | 1.94 | 0.05 | >.999 | 2.21 | 0.03 | >.999 | 2.85 | <.001 | 0.68 | 2.08 | 0.04 | >.999 | 0.46 | 0.64 | >.999 |
| Left Posterior Cingulate & Superior Transverse Temporal Gyrus | Right Posterior Cingulate & Superior Transverse Temporal Gyrus | 1.15 | 0.25 | >.999 | 0.75 | 0.46 | >.999 | 1.20 | 0.23 | >.999 | 1.60 | 0.11 | >.999 | 1.26 | 0.21 | >.999 |
| Left Posterior Cingulate & Superior Transverse Temporal Gyrus | Left Posterior Cingulate & Cuneus | 1.57 | 0.12 | >.999 | 1.69 | 0.09 | >.999 | 1.76 | 0.08 | >.999 | 2.56 | 0.01 | >.999 | 2.60 | 0.01 | >.999 |
| Left Posterior Cingulate & Superior Transverse Temporal Gyrus | Right Posterior Cingulate & Cuneus | 1.16 | 0.25 | >.999 | >.999 | 0.32 | >.999 | 1.66 | 0.10 | >.999 | 2.39 | 0.02 | >.999 | 2.47 | 0.01 | >.999 |
| Left Posterior Cingulate & Superior Transverse Temporal Gyrus | Left Medial Temporal Lobe & Parahippocampal Gyrus | 1.96 | 0.05 | >.999 | 1.93 | 0.06 | >.999 | 2.17 | 0.03 | >.999 | 2.47 | 0.01 | >.999 | 2.72 | 0.01 | >.999 |
| Left Posterior Cingulate & Superior Transverse Temporal Gyrus | Right Medial Temporal Lobe & Parahippocampal Gyrus | 1.28 | 0.20 | >.999 | 1.37 | 0.17 | >.999 | 1.78 | 0.08 | >.999 | 2.29 | 0.02 | >.999 | 2.56 | 0.01 | >.999 |
| Left Posterior Cingulate & Superior Transverse Temporal Gyrus | Left Angular Gyrus & Inferior Parietal Lobe | 0.74 | 0.46 | >.999 | 1.17 | 0.24 | >.999 | 1.31 | 0.19 | >.999 | 0.13 | 0.90 | >.999 | -1.19 | 0.24 | >.999 |
| Left Posterior Cingulate & Superior Transverse Temporal Gyrus | Right Angular Gyrus & Inferior Parietal Lobe | 1.58 | 0.12 | >.999 | 1.59 | 0.11 | >.999 | 2.34 | 0.02 | >.999 | 1.47 | 0.14 | >.999 | -0.09 | 0.92 | >.999 |
| Left Posterior Cingulate & Superior Transverse Temporal Gyrus | Left Inferior Parietal Lobe Angular Gyrus | 1.04 | 0.30 | >.999 | 1.24 | 0.22 | >.999 | 1.52 | 0.13 | >.999 | 0.39 | 0.70 | >.999 | -1.06 | 0.29 | >.999 |
| Left Posterior Cingulate & Superior Transverse Temporal Gyrus | Right Inferior Parietal Lobe Angular Gyrus | 0.43 | 0.67 | >.999 | 0.87 | 0.39 | >.999 | 1.99 | 0.05 | >.999 | 0.65 | 0.52 | >.999 | 0.07 | 0.94 | >.999 |
| Right Posterior Cingulate & Superior Transverse Temporal Gyrus | Left Posterior Cingulate & Cuneus | 1.12 | 0.26 | >.999 | 0.97 | 0.33 | >.999 | 1.49 | 0.14 | >.999 | 1.72 | 0.09 | >.999 | 1.53 | 0.13 | >.999 |
| Right Posterior Cingulate & Superior Transverse Temporal Gyrus | Right Posterior Cingulate & Cuneus | 1.94 | 0.05 | >.999 | 1.41 | 0.16 | >.999 | 1.02 | 0.31 | >.999 | 1.67 | 0.10 | >.999 | 1.60 | 0.11 | >.999 |
| Right Posterior Cingulate & Superior Transverse Temporal Gyrus | Left Medial Temporal Lobe & Parahippocampal Gyrus | 1.98 | 0.05 | >.999 | 1.92 | 0.06 | >.999 | 2.21 | 0.03 | >.999 | 2.05 | 0.04 | >.999 | 1.49 | 0.14 | >.999 |
| Right Posterior Cingulate & Superior Transverse Temporal Gyrus | Right Medial Temporal Lobe & Parahippocampal Gyrus | 1.86 | 0.06 | >.999 | 1.65 | 0.10 | >.999 | 1.36 | 0.17 | >.999 | 1.55 | 0.12 | >.999 | 1.26 | 0.21 | >.999 |
| Right Posterior Cingulate & Superior Transverse Temporal Gyrus | Left Angular Gyrus & Inferior Parietal Lobe | 1.46 | 0.15 | >.999 | 1.69 | 0.09 | >.999 | 1.89 | 0.06 | >.999 | 2.03 | 0.04 | >.999 | 1.15 | 0.25 | >.999 |
| Right Posterior Cingulate & Superior Transverse Temporal Gyrus | Right Angular Gyrus & Inferior Parietal Lobe | 1.82 | 0.07 | >.999 | 1.76 | 0.08 | >.999 | 2.42 | 0.02 | >.999 | 1.90 | 0.06 | >.999 | 0.50 | 0.62 | >.999 |
| Right Posterior Cingulate & Superior Transverse Temporal Gyrus | Left Inferior Parietal Lobe Angular Gyrus | 0.95 | 0.35 | >.999 | 1.42 | 0.16 | >.999 | 2.05 | 0.04 | >.999 | 1.64 | 0.10 | >.999 | 0.12 | 0.90 | >.999 |
| Right Posterior Cingulate & Superior Transverse Temporal Gyrus | Right Inferior Parietal Lobe Angular Gyrus | 0.31 | 0.76 | >.999 | 0.86 | 0.39 | >.999 | 1.74 | 0.08 | >.999 | 0.65 | 0.52 | >.999 | 0.04 | 0.97 | >.999 |
| Left Post Central Gyrus | Left Pre-Frontal Cortex | 1.24 | 0.22 | >.999 | 1.97 | 0.05 | >.999 | 3.29 | <.001 | 0.22 | 1.33 | 0.19 | >.999 | 0.09 | 0.93 | >.999 |
| Left Post Central Gyrus | Right Pre-Frontal Cortex | 1.56 | 0.12 | >.999 | 1.84 | 0.07 | >.999 | 2.84 | 0.01 | 0.69 | 1.28 | 0.20 | >.999 | 0.32 | 0.75 | >.999 |
| Left Post Central Gyrus | Left Orbital Frontal | 3.61 | <.001 | 0.08 | 3.21 | <.001 | 0.30 | 4.22* | <.001 | 0.01 | 2.91 | <.001 | 0.76 | 0.32 | 0.75 | >.999 |
| Left Post Central Gyrus | Right Orbital Frontal | 2.83 | 0.01 | 0.97 | 2.56 | 0.01 | >.999 | 3.73 | <.001 | 0.05 | 2.86 | <.001 | 0.87 | 0.74 | 0.46 | >.999 |
| Left Post Central Gyrus | Left Occipital Cortex | 2.59 | 0.01 | >.999 | 2.11 | 0.04 | >.999 | 2.21 | 0.03 | >.999 | 1.52 | 0.13 | >.999 | 0.32 | 0.75 | >.999 |
| Left Post Central Gyrus | Right Occipital Cortex | 1.93 | 0.06 | >.999 | 2.31 | 0.02 | >.999 | 3.07 | <.001 | 0.41 | 1.69 | 0.09 | >.999 | -0.21 | 0.83 | >.999 |
| Left Post Central Gyrus | Left Posterior Cingulate & Superior Transverse Temporal Gyrus | 1.93 | 0.06 | >.999 | 1.66 | 0.10 | >.999 | 1.94 | 0.05 | >.999 | 1.04 | 0.30 | >.999 | -0.55 | 0.58 | >.999 |
| Left Post Central Gyrus | Right Posterior Cingulate & Superior Transverse Temporal Gyrus | 1.62 | 0.11 | >.999 | 1.75 | 0.08 | >.999 | 2.44 | 0.02 | >.999 | 2.03 | 0.04 | >.999 | 0.03 | 0.98 | >.999 |
| Left Post Central Gyrus | Right Post Central Gyrus | -0.30 | 0.77 | >.999 | 0.53 | 0.59 | >.999 | 2.18 | 0.03 | >.999 | -0.11 | 0.91 | >.999 | -0.69 | 0.49 | >.999 |
| Left Post Central Gyrus | Left Posterior Cingulate & Cuneus | 3.19 | <.001 | 0.32 | 3.10 | <.001 | 0.42 | 3.05 | <.001 | 0.42 | 1.74 | 0.08 | >.999 | -0.16 | 0.87 | >.999 |
| Left Post Central Gyrus | Right Posterior Cingulate & Cuneus | 1.78 | 0.08 | >.999 | 2.04 | 0.04 | >.999 | 2.86 | <.001 | 0.68 | 1.99 | 0.05 | >.999 | 0.10 | 0.92 | >.999 |
| Left Post Central Gyrus | Left Medial Temporal Lobe & Parahippocampal Gyrus | 2.83 | 0.01 | 0.96 | 3.09 | <.001 | 0.44 | 3.18 | <.001 | 0.29 | 0.56 | 0.57 | >.999 | -1.08 | 0.28 | >.999 |
| Left Post Central Gyrus | Right Medial Temporal Lobe & Parahippocampal Gyrus | 2.31 | 0.02 | >.999 | 2.22 | 0.03 | >.999 | 3.04 | <.001 | 0.43 | 1.85 | 0.07 | >.999 | -0.05 | 0.96 | >.999 |
| Left Post Central Gyrus | Left Angular Gyrus & Inferior Parietal Lobe | -0.05 | 0.96 | >.999 | 0.33 | 0.75 | >.999 | 0.82 | 0.41 | >.999 | -0.63 | 0.53 | >.999 | -1.55 | 0.12 | >.999 |
| Left Post Central Gyrus | Right Angular Gyrus & Inferior Parietal Lobe | 0.93 | 0.36 | >.999 | 2.13 | 0.03 | >.999 | 2.99 | <.001 | 0.50 | 0.74 | 0.46 | >.999 | -0.73 | 0.46 | >.999 |
| Left Post Central Gyrus | Left Inferior Parietal Lobe Angular Gyrus | 0.58 | 0.57 | >.999 | 0.38 | 0.71 | >.999 | 0.53 | 0.60 | >.999 | 0.61 | 0.54 | >.999 | 0.19 | 0.85 | >.999 |
| Left Post Central Gyrus | Right Inferior Parietal Lobe Angular Gyrus | -0.47 | 0.64 | >.999 | 0.62 | 0.53 | >.999 | 2.43 | 0.02 | >.999 | -0.17 | 0.87 | >.999 | -0.73 | 0.46 | >.999 |
| Left Post Central Gyrus | Left Supramarginal Gyrus | -0.69 | 0.49 | >.999 | -0.41 | 0.68 | >.999 | 1.17 | 0.24 | >.999 | 0.23 | 0.82 | >.999 | -0.39 | 0.70 | >.999 |
| Left Post Central Gyrus | Right Supramarginal Gyrus | <.001 | >.999 | >.999 | 1.19 | 0.24 | >.999 | 2.98 | <.001 | 0.51 | -0.35 | 0.73 | >.999 | -1.27 | 0.20 | >.999 |
| Right Post Central Gyrus | Left Pre-Frontal Cortex | 1.71 | 0.09 | >.999 | 1.73 | 0.09 | >.999 | 2.64 | 0.01 | >.999 | 1.80 | 0.07 | >.999 | 1.47 | 0.14 | >.999 |
| Right Post Central Gyrus | Right Pre-Frontal Cortex | 1.75 | 0.08 | >.999 | 2.25 | 0.03 | >.999 | 2.65 | 0.01 | >.999 | 0.67 | 0.51 | >.999 | -0.13 | 0.90 | >.999 |
| Right Post Central Gyrus | Left Orbital Frontal | 2.11 | 0.04 | >.999 | 2.42 | 0.02 | >.999 | 3.23 | <.001 | 0.26 | 2.47 | 0.01 | >.999 | 1.60 | 0.11 | >.999 |
| Right Post Central Gyrus | Right Orbital Frontal | 2.16 | 0.03 | >.999 | 2.49 | 0.01 | >.999 | 3.24 | <.001 | 0.26 | 1.93 | 0.06 | >.999 | 0.72 | 0.47 | >.999 |
| Right Post Central Gyrus | Left Occipital Cortex | 0.41 | 0.68 | >.999 | 1.12 | 0.26 | >.999 | 2.54 | 0.01 | >.999 | 1.81 | 0.07 | >.999 | 0.99 | 0.32 | >.999 |
| Right Post Central Gyrus | Right Occipital Cortex | 1.96 | 0.05 | >.999 | 2.45 | 0.02 | >.999 | 2.91 | <.001 | 0.60 | 2.02 | 0.05 | >.999 | -0.02 | 0.98 | >.999 |
| Right Post Central Gyrus | Left Posterior Cingulate & Superior Transverse Temporal Gyrus | 0.27 | 0.78 | >.999 | 0.82 | 0.41 | >.999 | 1.99 | 0.05 | >.999 | 0.34 | 0.73 | >.999 | -0.08 | 0.94 | >.999 |
| Right Post Central Gyrus | Right Posterior Cingulate & Superior Transverse Temporal Gyrus | 0.21 | 0.83 | >.999 | 0.89 | 0.37 | >.999 | 1.71 | 0.09 | >.999 | 0.51 | 0.61 | >.999 | 0.08 | 0.94 | >.999 |
| Right Post Central Gyrus | Left Posterior Cingulate & Cuneus | 0.61 | 0.54 | >.999 | 0.78 | 0.44 | >.999 | 1.98 | 0.05 | >.999 | -0.26 | 0.80 | >.999 | -1.22 | 0.22 | >.999 |
| Right Post Central Gyrus | Right Posterior Cingulate & Cuneus | 1.20 | 0.23 | >.999 | 1.28 | 0.20 | >.999 | 1.98 | 0.05 | >.999 | 0.73 | 0.47 | >.999 | 0.05 | 0.96 | >.999 |
| Right Post Central Gyrus | Left Medial Temporal Lobe & Parahippocampal Gyrus | 1.08 | 0.28 | >.999 | 1.02 | 0.31 | >.999 | 2.12 | 0.04 | >.999 | -0.16 | 0.87 | >.999 | -0.89 | 0.38 | >.999 |
| Right Post Central Gyrus | Right Medial Temporal Lobe & Parahippocampal Gyrus | 1.28 | 0.20 | >.999 | 1.40 | 0.16 | >.999 | 2.11 | 0.04 | >.999 | 1.13 | 0.26 | >.999 | 0.69 | 0.49 | >.999 |
| Right Post Central Gyrus | Left Angular Gyrus & Inferior Parietal Lobe | 0.18 | 0.86 | >.999 | 1.98 | 0.05 | >.999 | 2.54 | 0.01 | >.999 | 0.95 | 0.34 | >.999 | 0.17 | 0.86 | >.999 |
| Right Post Central Gyrus | Right Angular Gyrus & Inferior Parietal Lobe | -0.47 | 0.64 | >.999 | 0.75 | 0.46 | >.999 | 1.91 | 0.06 | >.999 | 1.05 | 0.30 | >.999 | 0.37 | 0.71 | >.999 |
| Right Post Central Gyrus | Left Inferior Parietal Lobe Angular Gyrus | -0.53 | 0.60 | >.999 | 0.59 | 0.56 | >.999 | 2.12 | 0.04 | >.999 | -0.21 | 0.83 | >.999 | -0.46 | 0.65 | >.999 |
| Right Post Central Gyrus | Right Inferior Parietal Lobe Angular Gyrus | -0.09 | 0.93 | >.999 | 0.21 | 0.83 | >.999 | 0.22 | 0.82 | >.999 | 0.25 | 0.80 | >.999 | -0.06 | 0.95 | >.999 |
| Right Post Central Gyrus | Left Supramarginal Gyrus | 0.38 | 0.71 | >.999 | 1.35 | 0.18 | >.999 | 2.94 | <.001 | 0.56 | 0.25 | 0.81 | >.999 | -0.19 | 0.85 | >.999 |
| Right Post Central Gyrus | Right Supramarginal Gyrus | -1.55 | 0.12 | >.999 | -0.57 | 0.57 | >.999 | 1.18 | 0.24 | >.999 | 0.35 | 0.73 | >.999 | 0.57 | 0.57 | >.999 |
| Left Posterior Cingulate & Cuneus | Right Posterior Cingulate & Cuneus | 0.80 | 0.43 | >.999 | 0.55 | 0.59 | >.999 | 1.58 | 0.12 | >.999 | 1.75 | 0.08 | >.999 | 1.75 | 0.08 | >.999 |
| Left Posterior Cingulate & Cuneus | Left Medial Temporal Lobe & Parahippocampal Gyrus | 0.99 | 0.32 | >.999 | 0.28 | 0.78 | >.999 | 0.84 | 0.40 | >.999 | 0.20 | 0.84 | >.999 | 0.21 | 0.84 | >.999 |
| Left Posterior Cingulate & Cuneus | Right Medial Temporal Lobe & Parahippocampal Gyrus | 0.83 | 0.41 | >.999 | 0.56 | 0.57 | >.999 | 1.00 | 0.32 | >.999 | 1.14 | 0.26 | >.999 | 1.36 | 0.18 | >.999 |
| Left Posterior Cingulate & Cuneus | Left Angular Gyrus & Inferior Parietal Lobe | 1.62 | 0.11 | >.999 | 1.80 | 0.07 | >.999 | 2.01 | 0.05 | >.999 | 0.78 | 0.44 | >.999 | -0.13 | 0.90 | >.999 |
| Left Posterior Cingulate & Cuneus | Right Angular Gyrus & Inferior Parietal Lobe | 1.19 | 0.23 | >.999 | 1.27 | 0.21 | >.999 | 2.30 | 0.02 | >.999 | 0.50 | 0.62 | >.999 | -1.08 | 0.28 | >.999 |
| Left Posterior Cingulate & Cuneus | Left Inferior Parietal Lobe Angular Gyrus | 2.32 | 0.02 | >.999 | 2.48 | 0.01 | >.999 | 2.78 | 0.01 | 0.80 | 1.42 | 0.16 | >.999 | -0.19 | 0.85 | >.999 |
| Left Posterior Cingulate & Cuneus | Right Inferior Parietal Lobe Angular Gyrus | 0.63 | 0.53 | >.999 | 0.92 | 0.36 | >.999 | 2.14 | 0.03 | >.999 | -0.09 | 0.93 | >.999 | -1.35 | 0.18 | >.999 |
| Right Posterior Cingulate & Cuneus | Left Medial Temporal Lobe & Parahippocampal Gyrus | 1.80 | 0.07 | >.999 | 1.85 | 0.07 | >.999 | 2.39 | 0.02 | >.999 | 2.03 | 0.04 | >.999 | 1.48 | 0.14 | >.999 |
| Right Posterior Cingulate & Cuneus | Right Medial Temporal Lobe & Parahippocampal Gyrus | 1.72 | 0.09 | >.999 | 1.35 | 0.18 | >.999 | 0.90 | 0.37 | >.999 | 0.97 | 0.33 | >.999 | 0.10 | 0.92 | >.999 |
| Right Posterior Cingulate & Cuneus | Left Angular Gyrus & Inferior Parietal Lobe | 2.34 | 0.02 | >.999 | 2.36 | 0.02 | >.999 | 2.65 | 0.01 | >.999 | 2.44 | 0.02 | >.999 | 1.09 | 0.28 | >.999 |
| Right Posterior Cingulate & Cuneus | Right Angular Gyrus & Inferior Parietal Lobe | 2.19 | 0.03 | >.999 | 1.90 | 0.06 | >.999 | 2.57 | 0.01 | >.999 | 1.63 | 0.10 | >.999 | 0.36 | 0.72 | >.999 |
| Right Posterior Cingulate & Cuneus | Left Inferior Parietal Lobe Angular Gyrus | 1.27 | 0.21 | >.999 | 1.77 | 0.08 | >.999 | 2.62 | 0.01 | >.999 | 1.82 | 0.07 | >.999 | 0.13 | 0.89 | >.999 |
| Right Posterior Cingulate & Cuneus | Right Inferior Parietal Lobe Angular Gyrus | 1.36 | 0.18 | >.999 | 1.35 | 0.18 | >.999 | 2.15 | 0.03 | >.999 | 0.66 | 0.51 | >.999 | -0.32 | 0.75 | >.999 |
| Left Medial Temporal Lobe & Parahippocampal Gyrus | Right Medial Temporal Lobe & Parahippocampal Gyrus | 2.04 | 0.04 | >.999 | 2.00 | 0.05 | >.999 | 1.89 | 0.06 | >.999 | 2.22 | 0.03 | >.999 | 2.03 | 0.04 | >.999 |
| Left Medial Temporal Lobe & Parahippocampal Gyrus | Left Angular Gyrus & Inferior Parietal Lobe | 1.77 | 0.08 | >.999 | 1.52 | 0.13 | >.999 | 2.04 | 0.04 | >.999 | 0.49 | 0.63 | >.999 | 0.04 | 0.97 | >.999 |
| Left Medial Temporal Lobe & Parahippocampal Gyrus | Right Angular Gyrus & Inferior Parietal Lobe | 1.18 | 0.24 | >.999 | 1.48 | 0.14 | >.999 | 2.55 | 0.01 | >.999 | -0.18 | 0.85 | >.999 | -1.80 | 0.07 | >.999 |
| Left Medial Temporal Lobe & Parahippocampal Gyrus | Left Inferior Parietal Lobe Angular Gyrus | 2.24 | 0.03 | >.999 | 2.40 | 0.02 | >.999 | 2.91 | <.001 | 0.60 | 0.50 | 0.62 | >.999 | -0.75 | 0.46 | >.999 |
| Left Medial Temporal Lobe & Parahippocampal Gyrus | Right Inferior Parietal Lobe Angular Gyrus | 0.73 | 0.47 | >.999 | 1.08 | 0.28 | >.999 | 2.31 | 0.02 | >.999 | -0.43 | 0.67 | >.999 | -1.59 | 0.11 | >.999 |
| Right Medial Temporal Lobe & Parahippocampal Gyrus | Left Angular Gyrus & Inferior Parietal Lobe | 2.61 | 0.01 | >.999 | 2.67 | 0.01 | >.999 | 2.84 | 0.01 | 0.69 | 2.70 | 0.01 | >.999 | 1.35 | 0.18 | >.999 |
| Right Medial Temporal Lobe & Parahippocampal Gyrus | Right Angular Gyrus & Inferior Parietal Lobe | 1.88 | 0.06 | >.999 | 1.81 | 0.07 | >.999 | 2.59 | 0.01 | >.999 | 1.16 | 0.25 | >.999 | -0.44 | 0.66 | >.999 |
| Right Medial Temporal Lobe & Parahippocampal Gyrus | Left Inferior Parietal Lobe Angular Gyrus | 1.75 | 0.08 | >.999 | 1.85 | 0.07 | >.999 | 2.74 | 0.01 | 0.83 | 1.84 | 0.07 | >.999 | 0.28 | 0.78 | >.999 |
| Right Medial Temporal Lobe & Parahippocampal Gyrus | Right Inferior Parietal Lobe Angular Gyrus | 1.27 | 0.21 | >.999 | 1.42 | 0.16 | >.999 | 2.28 | 0.02 | >.999 | 0.72 | 0.47 | >.999 | -0.23 | 0.82 | >.999 |
| Left Angular Gyrus & Inferior Parietal Lobe | Right Angular Gyrus & Inferior Parietal Lobe | 0.32 | 0.75 | >.999 | 1.74 | 0.08 | >.999 | 2.86 | <.001 | 0.68 | 0.49 | 0.62 | >.999 | -0.22 | 0.83 | >.999 |
| Left Angular Gyrus & Inferior Parietal Lobe | Left Inferior Parietal Lobe Angular Gyrus | 0.11 | 0.91 | >.999 | 0.07 | 0.95 | >.999 | 0.47 | 0.64 | >.999 | -0.41 | 0.68 | >.999 | -0.73 | 0.47 | >.999 |
| Left Angular Gyrus & Inferior Parietal Lobe | Right Inferior Parietal Lobe Angular Gyrus | 0.24 | 0.81 | >.999 | 2.16 | 0.03 | >.999 | 2.76 | 0.01 | 0.83 | 0.89 | 0.37 | >.999 | -0.25 | 0.81 | >.999 |
| Right Angular Gyrus & Inferior Parietal Lobe | Left Inferior Parietal Lobe Angular Gyrus | 0.81 | 0.42 | >.999 | 2.42 | 0.02 | >.999 | 3.24 | <.001 | 0.26 | 0.72 | 0.48 | >.999 | -0.63 | 0.53 | >.999 |
| Right Angular Gyrus & Inferior Parietal Lobe | Right Inferior Parietal Lobe Angular Gyrus | 0.07 | 0.95 | >.999 | 0.95 | 0.34 | >.999 | 2.20 | 0.03 | >.999 | 1.72 | 0.09 | >.999 | 1.10 | 0.27 | >.999 |
| Left Inferior Parietal Lobe Angular Gyrus | Right Inferior Parietal Lobe Angular Gyrus | -0.62 | 0.54 | >.999 | 0.83 | 0.41 | >.999 | 2.43 | 0.02 | >.999 | -0.27 | 0.79 | >.999 | -0.69 | 0.49 | >.999 |
| Left Supramarginal Gyrus | Left Pre-Frontal Cortex | 2.41 | 0.02 | >.999 | 3.05 | <.001 | 0.49 | 3.05 | <.001 | 0.42 | 3.07 | <.001 | 0.48 | 1.02 | 0.31 | >.999 |
| Left Supramarginal Gyrus | Right Pre-Frontal Cortex | 2.26 | 0.02 | >.999 | 2.63 | 0.01 | >.999 | 3.25 | <.001 | 0.25 | 3.19 | <.001 | 0.32 | 1.76 | 0.08 | >.999 |
| Left Supramarginal Gyrus | Left Orbital Frontal | 3.73 | <.001 | 0.05 | 3.84* | <.001 | 0.04 | 3.84* | <.001 | 0.03 | 4.36* | <.001 | <.001 | 2.22 | 0.03 | >.999 |
| Left Supramarginal Gyrus | Right Orbital Frontal | 3.66 | <.001 | 0.07 | 3.61 | <.001 | 0.08 | 3.75 | <.001 | 0.05 | 4.65* | <.001 | <.001 | 3.02 | <.001 | 0.57 |
| Left Supramarginal Gyrus | Left Occipital Cortex | 1.47 | 0.14 | >.999 | 1.63 | 0.10 | >.999 | 2.34 | 0.02 | >.999 | 1.06 | 0.29 | >.999 | -1.35 | 0.18 | >.999 |
| Left Supramarginal Gyrus | Right Occipital Cortex | 2.29 | 0.02 | >.999 | 2.48 | 0.01 | >.999 | 2.98 | <.001 | 0.50 | 2.57 | 0.01 | >.999 | 0.64 | 0.52 | >.999 |
| Left Supramarginal Gyrus | Left Posterior Cingulate & Superior Transverse Temporal Gyrus | 0.86 | 0.39 | >.999 | 1.39 | 0.17 | >.999 | 2.11 | 0.04 | >.999 | 0.82 | 0.42 | >.999 | -0.32 | 0.75 | >.999 |
| Left Supramarginal Gyrus | Right Posterior Cingulate & Superior Transverse Temporal Gyrus | 1.02 | 0.31 | >.999 | 1.26 | 0.21 | >.999 | 2.05 | 0.04 | >.999 | 0.87 | 0.39 | >.999 | <.001 | >.999 | >.999 |
| Left Supramarginal Gyrus | Left Posterior Cingulate & Cuneus | 1.46 | 0.15 | >.999 | 1.92 | 0.06 | >.999 | 2.63 | 0.01 | >.999 | 1.86 | 0.06 | >.999 | 0.67 | 0.50 | >.999 |
| Left Supramarginal Gyrus | Right Posterior Cingulate & Cuneus | 1.29 | 0.20 | >.999 | 1.56 | 0.12 | >.999 | 2.57 | 0.01 | >.999 | 1.34 | 0.18 | >.999 | 0.06 | 0.96 | >.999 |
| Left Supramarginal Gyrus | Left Medial Temporal Lobe & Parahippocampal Gyrus | 2.11 | 0.04 | >.999 | 2.57 | 0.01 | >.999 | 3.20 | <.001 | 0.28 | 2.43 | 0.02 | >.999 | 1.06 | 0.29 | >.999 |
| Left Supramarginal Gyrus | Right Medial Temporal Lobe & Parahippocampal Gyrus | 1.51 | 0.13 | >.999 | 2.08 | 0.04 | >.999 | 2.99 | <.001 | 0.50 | 1.85 | 0.07 | >.999 | 0.52 | 0.60 | >.999 |
| Left Supramarginal Gyrus | Left Angular Gyrus & Inferior Parietal Lobe | 0.47 | 0.64 | >.999 | 1.01 | 0.32 | >.999 | 1.69 | 0.09 | >.999 | 0.90 | 0.37 | >.999 | -0.73 | 0.46 | >.999 |
| Left Supramarginal Gyrus | Right Angular Gyrus & Inferior Parietal Lobe | 1.45 | 0.15 | >.999 | 1.93 | 0.06 | >.999 | 2.97 | <.001 | 0.52 | 1.67 | 0.10 | >.999 | -0.75 | 0.45 | >.999 |
| Left Supramarginal Gyrus | Left Inferior Parietal Lobe Angular Gyrus | -0.79 | 0.43 | >.999 | -0.45 | 0.65 | >.999 | 0.87 | 0.39 | >.999 | -0.03 | 0.98 | >.999 | -0.56 | 0.58 | >.999 |
| Left Supramarginal Gyrus | Right Inferior Parietal Lobe Angular Gyrus | 0.39 | 0.70 | >.999 | 1.21 | 0.23 | >.999 | 2.76 | 0.01 | 0.83 | 0.45 | 0.66 | >.999 | -0.37 | 0.72 | >.999 |
| Left Supramarginal Gyrus | Right Supramarginal Gyrus | 0.81 | 0.42 | >.999 | 1.30 | 0.20 | >.999 | 2.09 | 0.04 | >.999 | 0.88 | 0.38 | >.999 | 0.22 | 0.83 | >.999 |
| Right Supramarginal Gyrus | Left Pre-Frontal Cortex | 1.98 | 0.05 | >.999 | 2.21 | 0.03 | >.999 | 3.23 | <.001 | 0.26 | 1.47 | 0.14 | >.999 | 0.09 | 0.92 | >.999 |
| Right Supramarginal Gyrus | Right Pre-Frontal Cortex | 2.39 | 0.02 | >.999 | 2.88 | <.001 | 0.79 | 3.42 | <.001 | 0.15 | 2.62 | 0.01 | >.999 | 0.99 | 0.32 | >.999 |
| Right Supramarginal Gyrus | Left Orbital Frontal | 2.58 | 0.01 | >.999 | 3.28 | <.001 | 0.24 | 3.65 | <.001 | 0.07 | 2.18 | 0.03 | >.999 | 0.30 | 0.76 | >.999 |
| Right Supramarginal Gyrus | Right Orbital Frontal | 1.87 | 0.06 | >.999 | 2.91 | <.001 | 0.75 | 3.49 | <.001 | 0.12 | 2.58 | 0.01 | >.999 | 0.68 | 0.50 | >.999 |
| Right Supramarginal Gyrus | Left Occipital Cortex | 1.63 | 0.11 | >.999 | 1.50 | 0.13 | >.999 | 2.69 | 0.01 | 0.95 | 1.72 | 0.09 | >.999 | -0.39 | 0.69 | >.999 |
| Right Supramarginal Gyrus | Right Occipital Cortex | 1.26 | 0.21 | >.999 | 1.88 | 0.06 | >.999 | 2.91 | <.001 | 0.60 | 1.37 | 0.17 | >.999 | -0.85 | 0.40 | >.999 |
| Right Supramarginal Gyrus | Left Posterior Cingulate & Superior Transverse Temporal Gyrus | 1.44 | 0.15 | >.999 | 1.80 | 0.07 | >.999 | 2.74 | 0.01 | 0.84 | 1.01 | 0.32 | >.999 | -0.22 | 0.83 | >.999 |
| Right Supramarginal Gyrus | Right Posterior Cingulate & Superior Transverse Temporal Gyrus | 0.98 | 0.33 | >.999 | 1.59 | 0.11 | >.999 | 2.34 | 0.02 | >.999 | 0.70 | 0.48 | >.999 | -0.43 | 0.67 | >.999 |
| Right Supramarginal Gyrus | Left Posterior Cingulate & Cuneus | 1.46 | 0.15 | >.999 | 1.73 | 0.09 | >.999 | 2.63 | 0.01 | >.999 | 0.90 | 0.37 | >.999 | -0.53 | 0.60 | >.999 |
| Right Supramarginal Gyrus | Right Posterior Cingulate & Cuneus | 1.73 | 0.09 | >.999 | 1.90 | 0.06 | >.999 | 2.75 | 0.01 | 0.83 | 1.53 | 0.13 | >.999 | 0.36 | 0.72 | >.999 |
| Right Supramarginal Gyrus | Left Medial Temporal Lobe & Parahippocampal Gyrus | 1.32 | 0.19 | >.999 | 2.03 | 0.04 | >.999 | 2.78 | 0.01 | 0.80 | 1.07 | 0.29 | >.999 | -0.45 | 0.65 | >.999 |
| Right Supramarginal Gyrus | Right Medial Temporal Lobe & Parahippocampal Gyrus | 1.57 | 0.12 | >.999 | 2.18 | 0.03 | >.999 | 3.10 | <.001 | 0.37 | 1.95 | 0.05 | >.999 | 0.32 | 0.75 | >.999 |
| Right Supramarginal Gyrus | Left Angular Gyrus & Inferior Parietal Lobe | 1.35 | 0.18 | >.999 | 2.18 | 0.03 | >.999 | 2.93 | <.001 | 0.58 | 1.60 | 0.11 | >.999 | 0.08 | 0.94 | >.999 |
| Right Supramarginal Gyrus | Right Angular Gyrus & Inferior Parietal Lobe | -0.16 | 0.87 | >.999 | 0.80 | 0.42 | >.999 | 2.34 | 0.02 | >.999 | 0.93 | 0.36 | >.999 | 0.10 | 0.92 | >.999 |
| Right Supramarginal Gyrus | Left Inferior Parietal Lobe Angular Gyrus | 0.32 | 0.75 | >.999 | 1.51 | 0.13 | >.999 | 3.01 | <.001 | 0.47 | 0.34 | 0.74 | >.999 | -0.50 | 0.62 | >.999 |
| Right Supramarginal Gyrus | Right Inferior Parietal Lobe Angular Gyrus | -1.60 | 0.11 | >.999 | -0.63 | 0.53 | >.999 | 1.21 | 0.23 | >.999 | 0.21 | 0.84 | >.999 | 0.34 | 0.73 | >.999 |

P_BH_ = The Bonferroni-holm corrected post-hoc comparison was used; *P_BH_ < 0.05.

**Supplementary table S2.** Group comparisons of EEG source connectivity within reward salience network between IGD and HC groups

| **node** | | **Delta** | | | **Theta** | | | **Alpha** | | | **Beta** | | | **Gamma** | | |
| --- | --- | --- | --- | --- | --- | --- | --- | --- | --- | --- | --- | --- | --- | --- | --- | --- |
|  |  | **t** | **P** | **P_BH_** | **t** | **P** | **t** | **t** | **P** | **P_BH_** | **t** | **P** | **P_BH_** | **t** | **P** | **P_BH_** |
| Left Pre-Frontal Cortex | Right Pre-Frontal Cortex | -0.28 | 0.78 | >.999 | -0.67 | 0.50 | >.999 | -1.01 | 0.31 | >.999 | -0.75 | 0.46 | >.999 | -0.12 | 0.90 | >.999 |
| Left Pre-Frontal Cortex | Left Insula | 0.86 | 0.39 | >.999 | 0.66 | 0.51 | >.999 | 1.74 | 0.08 | >.999 | 0.89 | 0.37 | >.999 | 0.61 | 0.54 | >.999 |
| Left Pre-Frontal Cortex | Right Insula | 0.69 | 0.49 | >.999 | 1.21 | 0.23 | >.999 | 1.63 | 0.11 | >.999 | 0.44 | 0.66 | >.999 | 0.06 | 0.95 | >.999 |
| Left Pre-Frontal Cortex | Left Temporal Lobe | 1.62 | 0.11 | >.999 | 1.49 | 0.14 | >.999 | 2.34 | 0.02 | >.999 | 1.86 | 0.06 | >.999 | 1.14 | 0.26 | >.999 |
| Left Pre-Frontal Cortex | Right Temporal Lobe | 2.27 | 0.02 | >.999 | 2.07 | 0.04 | >.999 | 2.86 | <.001 | >.999 | 1.82 | 0.07 | >.999 | 0.12 | 0.91 | >.999 |
| Left Pre-Frontal Cortex | Left Posterior Cingulate Gyrus | 3.24 | <.001 | 0.56 | 2.87 | <.001 | >.999 | 3.42 | <.001 | 0.31 | 3.42 | <.001 | 0.30 | 2.62 | 0.01 | >.999 |
| Left Pre-Frontal Cortex | Right Posterior Cingulate Gyrus | 3.05 | <.001 | >.999 | 2.63 | 0.01 | >.999 | 3.27 | <.001 | 0.50 | 3.03 | <.001 | >.999 | 2.29 | 0.02 | >.999 |
| Left Pre-Frontal Cortex | Left Anterior Cingulate Gyrus | 2.50 | 0.01 | >.999 | 2.60 | 0.01 | >.999 | 3.30 | <.001 | 0.45 | 2.52 | 0.01 | >.999 | 1.92 | 0.06 | >.999 |
| Left Pre-Frontal Cortex | Right Anterior Cingulate Gyrus | 2.27 | 0.02 | >.999 | 2.40 | 0.02 | >.999 | 2.82 | 0.01 | >.999 | 2.14 | 0.03 | >.999 | 1.57 | 0.12 | >.999 |
| Left Pre-Frontal Cortex | Left Anterior Cingulate Gyrus | 0.40 | 0.69 | >.999 | 0.50 | 0.62 | >.999 | 1.37 | 0.17 | >.999 | 0.57 | 0.57 | >.999 | 0.30 | 0.77 | >.999 |
| Left Pre-Frontal Cortex | Right Anterior Cingulate Gyrus | 0.54 | 0.59 | >.999 | 0.68 | 0.50 | >.999 | 1.48 | 0.14 | >.999 | 0.93 | 0.35 | >.999 | 0.72 | 0.48 | >.999 |
| Left Pre-Frontal Cortex | Left Posterior Cingulate & Superior Transverse Temporal Gyrus | 2.29 | 0.02 | >.999 | 2.16 | 0.03 | >.999 | 2.92 | <.001 | >.999 | 2.64 | 0.01 | >.999 | 1.82 | 0.07 | >.999 |
| Left Pre-Frontal Cortex | Right Posterior Cingulate & Superior Transverse Temporal Gyrus | 2.18 | 0.03 | >.999 | 1.77 | 0.08 | >.999 | 2.68 | 0.01 | >.999 | 2.08 | 0.04 | >.999 | 1.19 | 0.24 | >.999 |
| Left Pre-Frontal Cortex | Left Posterior Cingulate & Cuneus | 1.65 | 0.10 | >.999 | 1.39 | 0.17 | >.999 | 2.27 | 0.02 | >.999 | 1.95 | 0.05 | >.999 | 1.46 | 0.15 | >.999 |
| Left Pre-Frontal Cortex | Right Posterior Cingulate & Cuneus | 1.74 | 0.08 | >.999 | 1.27 | 0.21 | >.999 | 2.14 | 0.03 | >.999 | 1.77 | 0.08 | >.999 | 1.09 | 0.28 | >.999 |
| Left Pre-Frontal Cortex | Left Anterior Cingulate Gyrus | 0.91 | 0.36 | >.999 | 1.35 | 0.18 | >.999 | 2.03 | 0.04 | >.999 | -0.35 | 0.73 | >.999 | -1.25 | 0.21 | >.999 |
| Left Pre-Frontal Cortex | Right Anterior Cingulate Gyrus | 0.82 | 0.41 | >.999 | 1.13 | 0.26 | >.999 | 1.14 | 0.26 | >.999 | 0.27 | 0.79 | >.999 | -0.08 | 0.93 | >.999 |
| Right Pre-Frontal Cortex | Left Insula | 0.82 | 0.41 | >.999 | 0.47 | 0.64 | >.999 | 1.05 | 0.29 | >.999 | 0.80 | 0.43 | >.999 | 0.72 | 0.47 | >.999 |
| Right Pre-Frontal Cortex | Right Insula | 0.42 | 0.68 | >.999 | 0.96 | 0.34 | >.999 | 1.49 | 0.14 | >.999 | 0.45 | 0.65 | >.999 | -0.08 | 0.94 | >.999 |
| Right Pre-Frontal Cortex | Left Temporal Lobe | 1.55 | 0.12 | >.999 | 1.29 | 0.20 | >.999 | 1.91 | 0.06 | >.999 | 2.46 | 0.01 | >.999 | 1.90 | 0.06 | >.999 |
| Right Pre-Frontal Cortex | Right Temporal Lobe | 1.73 | 0.09 | >.999 | 1.85 | 0.07 | >.999 | 2.26 | 0.03 | >.999 | 1.33 | 0.19 | >.999 | -0.03 | 0.97 | >.999 |
| Right Pre-Frontal Cortex | Left Posterior Cingulate Gyrus | 1.80 | 0.07 | >.999 | 1.94 | 0.05 | >.999 | 2.55 | 0.01 | >.999 | 3.16 | <.001 | 0.70 | 2.48 | 0.01 | >.999 |
| Right Pre-Frontal Cortex | Right Posterior Cingulate Gyrus | 1.63 | 0.10 | >.999 | 1.79 | 0.08 | >.999 | 2.36 | 0.02 | >.999 | 2.63 | 0.01 | >.999 | 1.85 | 0.07 | >.999 |
| Right Pre-Frontal Cortex | Left Anterior Cingulate Gyrus | 1.23 | 0.22 | >.999 | 1.19 | 0.24 | >.999 | 1.79 | 0.08 | >.999 | 2.47 | 0.01 | >.999 | 2.27 | 0.02 | >.999 |
| Right Pre-Frontal Cortex | Right Anterior Cingulate Gyrus | 2.06 | 0.04 | >.999 | 2.09 | 0.04 | >.999 | 2.64 | 0.01 | >.999 | 2.62 | 0.01 | >.999 | 2.10 | 0.04 | >.999 |
| Right Pre-Frontal Cortex | Left Anterior Cingulate Gyrus | 0.12 | 0.90 | >.999 | -0.27 | 0.79 | >.999 | 0.14 | 0.89 | >.999 | 0.65 | 0.52 | >.999 | 1.27 | 0.21 | >.999 |
| Right Pre-Frontal Cortex | Right Anterior Cingulate Gyrus | -0.38 | 0.70 | >.999 | -0.71 | 0.48 | >.999 | -0.29 | 0.78 | >.999 | 0.09 | 0.93 | >.999 | 0.60 | 0.55 | >.999 |
| Right Pre-Frontal Cortex | Left Posterior Cingulate & Superior Transverse Temporal Gyrus | 1.26 | 0.21 | >.999 | 1.28 | 0.20 | >.999 | 2.17 | 0.03 | >.999 | 2.59 | 0.01 | >.999 | 1.90 | 0.06 | >.999 |
| Right Pre-Frontal Cortex | Right Posterior Cingulate & Superior Transverse Temporal Gyrus | 0.67 | 0.51 | >.999 | 0.90 | 0.37 | >.999 | 1.92 | 0.06 | >.999 | 1.64 | 0.10 | >.999 | 0.74 | 0.46 | >.999 |
| Right Pre-Frontal Cortex | Left Posterior Cingulate & Cuneus | 1.44 | 0.15 | >.999 | 0.83 | 0.41 | >.999 | 1.34 | 0.18 | >.999 | 1.47 | 0.14 | >.999 | 1.17 | 0.24 | >.999 |
| Right Pre-Frontal Cortex | Right Posterior Cingulate & Cuneus | 0.38 | 0.70 | >.999 | 0.40 | 0.69 | >.999 | 1.11 | 0.27 | >.999 | 0.81 | 0.42 | >.999 | 0.37 | 0.72 | >.999 |
| Right Pre-Frontal Cortex | Left Anterior Cingulate Gyrus | 0.37 | 0.71 | >.999 | 0.60 | 0.55 | >.999 | 1.37 | 0.17 | >.999 | 0.78 | 0.44 | >.999 | 0.72 | 0.47 | >.999 |
| Right Pre-Frontal Cortex | Right Anterior Cingulate Gyrus | 1.15 | 0.25 | >.999 | 1.38 | 0.17 | >.999 | 1.33 | 0.19 | >.999 | 1.10 | 0.27 | >.999 | 0.77 | 0.44 | >.999 |
| Left Insula | Right Insula | 2.34 | 0.02 | >.999 | 2.34 | 0.02 | >.999 | 2.74 | 0.01 | >.999 | 2.65 | 0.01 | >.999 | 2.22 | 0.03 | >.999 |
| Left Insula | Left Parasubiculum hippocampal region | 1.90 | 0.06 | >.999 | 1.59 | 0.11 | >.999 | 1.99 | 0.05 | >.999 | 0.01 | >.999 | >.999 | -1.09 | 0.28 | >.999 |
| Left Insula | Right Parasubiculum hippocampal region | 2.09 | 0.04 | >.999 | 1.99 | 0.05 | >.999 | 2.26 | 0.03 | >.999 | 0.81 | 0.42 | >.999 | 0.05 | 0.96 | >.999 |
| Right Insula | Left Parasubiculum hippocampal region | 2.32 | 0.02 | >.999 | 2.93 | <.001 | >.999 | 2.73 | 0.01 | >.999 | 2.81 | 0.01 | >.999 | 2.61 | 0.01 | >.999 |
| Right Insula | Right Parasubiculum hippocampal region | 2.61 | 0.01 | >.999 | 3.25 | <.001 | 0.54 | 2.67 | 0.01 | >.999 | 2.35 | 0.02 | >.999 | 1.65 | 0.10 | >.999 |
| Left Temporal Lobe | Left Insula | 1.58 | 0.12 | >.999 | 0.87 | 0.39 | >.999 | 1.58 | 0.12 | >.999 | 2.14 | 0.03 | >.999 | 2.01 | 0.05 | >.999 |
| Left Temporal Lobe | Right Insula | 0.87 | 0.39 | >.999 | 1.55 | 0.12 | >.999 | 2.72 | 0.01 | >.999 | 2.98 | <.001 | >.999 | 2.14 | 0.03 | >.999 |
| Left Temporal Lobe | Right Temporal Lobe | 1.22 | 0.22 | >.999 | 1.91 | 0.06 | >.999 | 2.38 | 0.02 | >.999 | 2.06 | 0.04 | >.999 | 0.80 | 0.42 | >.999 |
| Left Temporal Lobe | Left Posterior Cingulate Gyrus | 0.50 | 0.62 | >.999 | 0.88 | 0.38 | >.999 | 1.42 | 0.16 | >.999 | 1.07 | 0.29 | >.999 | 0.69 | 0.49 | >.999 |
| Left Temporal Lobe | Right Posterior Cingulate Gyrus | 0.54 | 0.59 | >.999 | 1.00 | 0.32 | >.999 | 1.70 | 0.09 | >.999 | 1.44 | 0.15 | >.999 | 1.12 | 0.26 | >.999 |
| Left Temporal Lobe | Left Anterior Cingulate Gyrus | 1.13 | 0.26 | >.999 | 0.83 | 0.41 | >.999 | 1.99 | 0.05 | >.999 | 2.06 | 0.04 | >.999 | 1.21 | 0.23 | >.999 |
| Left Temporal Lobe | Right Anterior Cingulate Gyrus | 1.00 | 0.32 | >.999 | 0.86 | 0.39 | >.999 | 2.09 | 0.04 | >.999 | 2.58 | 0.01 | >.999 | 1.59 | 0.11 | >.999 |
| Left Temporal Lobe | Left Anterior Cingulate Gyrus | 1.40 | 0.16 | >.999 | 1.16 | 0.25 | >.999 | 2.18 | 0.03 | >.999 | 2.97 | <.001 | >.999 | 2.53 | 0.01 | >.999 |
| Left Temporal Lobe | Right Anterior Cingulate Gyrus | 1.38 | 0.17 | >.999 | 1.50 | 0.14 | >.999 | 2.61 | 0.01 | >.999 | 3.19 | <.001 | 0.64 | 2.46 | 0.01 | >.999 |
| Left Temporal Lobe | Left Posterior Cingulate & Superior Transverse Temporal Gyrus | 0.44 | 0.66 | >.999 | 0.62 | 0.54 | >.999 | 0.83 | 0.41 | >.999 | 0.39 | 0.70 | >.999 | 0.25 | 0.80 | >.999 |
| Left Temporal Lobe | Right Posterior Cingulate & Superior Transverse Temporal Gyrus | 0.62 | 0.53 | >.999 | 0.97 | 0.33 | >.999 | 1.62 | 0.11 | >.999 | 1.55 | 0.12 | >.999 | 1.41 | 0.16 | >.999 |
| Left Temporal Lobe | Left Posterior Cingulate & Cuneus | 1.02 | 0.31 | >.999 | 1.13 | 0.26 | >.999 | 1.54 | 0.13 | >.999 | 1.61 | 0.11 | >.999 | 1.61 | 0.11 | >.999 |
| Left Temporal Lobe | Right Posterior Cingulate & Cuneus | 1.35 | 0.18 | >.999 | 1.48 | 0.14 | >.999 | 2.19 | 0.03 | >.999 | 2.44 | 0.02 | >.999 | 1.99 | 0.05 | >.999 |
| Left Temporal Lobe | Left Anterior Cingulate Gyrus | 1.75 | 0.08 | >.999 | 1.53 | 0.13 | >.999 | 2.62 | 0.01 | >.999 | 1.76 | 0.08 | >.999 | 0.20 | 0.84 | >.999 |
| Left Temporal Lobe | Right Anterior Cingulate Gyrus | 1.39 | 0.17 | >.999 | 0.98 | 0.33 | >.999 | 1.89 | 0.06 | >.999 | 2.23 | 0.03 | >.999 | 0.81 | 0.42 | >.999 |
| Right Temporal Lobe | Left Insula | 2.94 | <.001 | >.999 | 2.44 | 0.02 | >.999 | 2.94 | <.001 | >.999 | 2.54 | 0.01 | >.999 | 1.06 | 0.29 | >.999 |
| Right Temporal Lobe | Right Insula | 2.99 | <.001 | >.999 | 3.13 | <.001 | 0.79 | 3.66 | <.001 | 0.14 | 3.82 | <.001 | 0.08 | 2.82 | 0.01 | >.999 |
| Right Temporal Lobe | Left Posterior Cingulate Gyrus | 2.58 | 0.01 | >.999 | 2.86 | <.001 | >.999 | 3.16 | <.001 | 0.70 | 2.69 | 0.01 | >.999 | 1.52 | 0.13 | >.999 |
| Right Temporal Lobe | Right Posterior Cingulate Gyrus | 2.80 | 0.01 | >.999 | 3.02 | <.001 | >.999 | 3.12 | <.001 | 0.78 | 2.82 | 0.01 | >.999 | 1.78 | 0.08 | >.999 |
| Right Temporal Lobe | Left Anterior Cingulate Gyrus | 2.38 | 0.02 | >.999 | 1.78 | 0.08 | >.999 | 2.86 | <.001 | >.999 | 1.92 | 0.06 | >.999 | 0.25 | 0.80 | >.999 |
| Right Temporal Lobe | Right Anterior Cingulate Gyrus | 2.20 | 0.03 | >.999 | 1.79 | 0.08 | >.999 | 2.68 | 0.01 | >.999 | 2.03 | 0.04 | >.999 | 0.38 | 0.70 | >.999 |
| Right Temporal Lobe | Left Anterior Cingulate Gyrus | 3.70 | <.001 | 0.12 | 3.41 | <.001 | 0.32 | 3.95 | <.001 | 0.05 | 3.63 | <.001 | 0.15 | 2.15 | 0.03 | >.999 |
| Right Temporal Lobe | Right Anterior Cingulate Gyrus | 3.56 | <.001 | 0.19 | 3.36 | <.001 | 0.38 | 3.99* | <.001 | 0.04 | 3.43 | <.001 | 0.30 | 1.99 | 0.05 | >.999 |
| Right Temporal Lobe | Left Posterior Cingulate & Superior Transverse Temporal Gyrus | 2.05 | 0.04 | >.999 | 2.40 | 0.02 | >.999 | 2.65 | 0.01 | >.999 | 2.02 | 0.05 | >.999 | 0.94 | 0.35 | >.999 |
| Right Temporal Lobe | Right Posterior Cingulate & Superior Transverse Temporal Gyrus | 2.82 | 0.01 | >.999 | 3.12 | <.001 | 0.81 | 2.93 | <.001 | >.999 | 2.68 | 0.01 | >.999 | 1.69 | 0.09 | >.999 |
| Right Temporal Lobe | Left Posterior Cingulate & Cuneus | 2.44 | 0.02 | >.999 | 2.57 | 0.01 | >.999 | 2.83 | 0.01 | >.999 | 2.17 | 0.03 | >.999 | 0.81 | 0.42 | >.999 |
| Right Temporal Lobe | Right Posterior Cingulate & Cuneus | 3.34 | <.001 | 0.41 | 3.48 | <.001 | 0.25 | 3.30 | <.001 | 0.45 | 2.83 | 0.01 | >.999 | 1.27 | 0.21 | >.999 |
| Right Temporal Lobe | Left Anterior Cingulate Gyrus | 1.46 | 0.15 | >.999 | 1.30 | 0.19 | >.999 | 2.43 | 0.02 | >.999 | 1.05 | 0.29 | >.999 | -1.22 | 0.23 | >.999 |
| Right Temporal Lobe | Right Anterior Cingulate Gyrus | 0.72 | 0.47 | >.999 | 0.75 | 0.45 | >.999 | 2.03 | 0.04 | >.999 | 0.33 | 0.74 | >.999 | -1.65 | 0.10 | >.999 |
| Left Posterior Cingulate Gyrus | Left Insula | 3.04 | <.001 | >.999 | 2.97 | <.001 | >.999 | 3.15 | <.001 | 0.73 | 2.45 | 0.02 | >.999 | 1.59 | 0.11 | >.999 |
| Left Posterior Cingulate Gyrus | Right Insula | 2.44 | 0.02 | >.999 | 3.07 | <.001 | 0.93 | 2.97 | <.001 | >.999 | 2.99 | <.001 | >.999 | 2.25 | 0.03 | >.999 |
| Left Posterior Cingulate Gyrus | Right Posterior Cingulate Gyrus | 2.18 | 0.03 | >.999 | 1.77 | 0.08 | >.999 | 2.01 | 0.05 | >.999 | 2.32 | 0.02 | >.999 | 2.05 | 0.04 | >.999 |
| Left Posterior Cingulate Gyrus | Left Anterior Cingulate Gyrus | 2.68 | 0.01 | >.999 | 1.59 | 0.11 | >.999 | 2.07 | 0.04 | >.999 | 2.51 | 0.01 | >.999 | 2.53 | 0.01 | >.999 |
| Left Posterior Cingulate Gyrus | Right Anterior Cingulate Gyrus | 1.95 | 0.05 | >.999 | 1.07 | 0.28 | >.999 | 1.62 | 0.11 | >.999 | 2.01 | 0.05 | >.999 | 2.32 | 0.02 | >.999 |
| Left Posterior Cingulate Gyrus | Left Anterior Cingulate Gyrus | 3.45 | <.001 | 0.29 | 3.34 | <.001 | 0.40 | 3.27 | <.001 | 0.50 | 3.79 | <.001 | 0.09 | 3.67 | <.001 | 0.13 |
| Left Posterior Cingulate Gyrus | Right Anterior Cingulate Gyrus | 3.17 | <.001 | 0.72 | 3.18 | <.001 | 0.68 | 3.09 | <.001 | 0.87 | 3.53 | <.001 | 0.21 | 3.57 | <.001 | 0.18 |
| Left Posterior Cingulate Gyrus | Left Posterior Cingulate & Superior Transverse Temporal Gyrus | 2.02 | 0.05 | >.999 | 1.81 | 0.07 | >.999 | 1.48 | 0.14 | >.999 | 2.18 | 0.03 | >.999 | 1.85 | 0.07 | >.999 |
| Left Posterior Cingulate Gyrus | Right Posterior Cingulate & Superior Transverse Temporal Gyrus | 1.69 | 0.09 | >.999 | 1.39 | 0.17 | >.999 | 1.46 | 0.15 | >.999 | 2.24 | 0.03 | >.999 | 2.01 | 0.05 | >.999 |
| Left Posterior Cingulate Gyrus | Left Posterior Cingulate & Cuneus | 1.91 | 0.06 | >.999 | 1.85 | 0.07 | >.999 | 1.83 | 0.07 | >.999 | 2.55 | 0.01 | >.999 | 2.53 | 0.01 | >.999 |
| Left Posterior Cingulate Gyrus | Right Posterior Cingulate & Cuneus | 1.88 | 0.06 | >.999 | 1.62 | 0.11 | >.999 | 1.88 | 0.06 | >.999 | 2.63 | 0.01 | >.999 | 2.44 | 0.02 | >.999 |
| Left Posterior Cingulate Gyrus | Left Anterior Cingulate Gyrus | 2.93 | <.001 | >.999 | 1.84 | 0.07 | >.999 | 2.72 | 0.01 | >.999 | 3.03 | <.001 | >.999 | 1.95 | 0.05 | >.999 |
| Left Posterior Cingulate Gyrus | Right Anterior Cingulate Gyrus | 1.39 | 0.17 | >.999 | 0.56 | 0.58 | >.999 | 1.52 | 0.13 | >.999 | 2.16 | 0.03 | >.999 | 1.86 | 0.07 | >.999 |
| Right Posterior Cingulate Gyrus | Left Insula | 2.86 | <.001 | >.999 | 2.88 | <.001 | >.999 | 3.00 | <.001 | >.999 | 2.07 | 0.04 | >.999 | 1.00 | 0.32 | >.999 |
| Right Posterior Cingulate Gyrus | Right Insula | 2.53 | 0.01 | >.999 | 3.26 | <.001 | 0.53 | 3.03 | <.001 | >.999 | 2.82 | 0.01 | >.999 | 1.94 | 0.05 | >.999 |
| Right Posterior Cingulate Gyrus | Left Anterior Cingulate Gyrus | 2.71 | 0.01 | >.999 | 1.55 | 0.12 | >.999 | 1.99 | 0.05 | >.999 | 2.44 | 0.02 | >.999 | 2.65 | 0.01 | >.999 |
| Right Posterior Cingulate Gyrus | Right Anterior Cingulate Gyrus | 1.96 | 0.05 | >.999 | 1.01 | 0.31 | >.999 | 1.47 | 0.14 | >.999 | 1.78 | 0.08 | >.999 | 2.19 | 0.03 | >.999 |
| Right Posterior Cingulate Gyrus | Left Anterior Cingulate Gyrus | 3.31 | <.001 | 0.45 | 3.29 | <.001 | 0.48 | 3.16 | <.001 | 0.69 | 3.38 | <.001 | 0.36 | 3.06 | <.001 | >.999 |
| Right Posterior Cingulate Gyrus | Right Anterior Cingulate Gyrus | 3.04 | <.001 | >.999 | 3.10 | <.001 | 0.87 | 2.93 | <.001 | >.999 | 3.07 | <.001 | 0.94 | 2.90 | <.001 | >.999 |
| Right Posterior Cingulate Gyrus | Left Posterior Cingulate & Superior Transverse Temporal Gyrus | 1.90 | 0.06 | >.999 | 1.62 | 0.11 | >.999 | 1.63 | 0.11 | >.999 | 1.85 | 0.07 | >.999 | 1.43 | 0.16 | >.999 |
| Right Posterior Cingulate Gyrus | Right Posterior Cingulate & Superior Transverse Temporal Gyrus | 2.09 | 0.04 | >.999 | 1.67 | 0.10 | >.999 | 1.39 | 0.17 | >.999 | 1.92 | 0.06 | >.999 | 1.61 | 0.11 | >.999 |
| Right Posterior Cingulate Gyrus | Left Posterior Cingulate & Cuneus | 1.75 | 0.08 | >.999 | 1.56 | 0.12 | >.999 | 1.64 | 0.10 | >.999 | 1.99 | 0.05 | >.999 | 1.81 | 0.07 | >.999 |
| Right Posterior Cingulate Gyrus | Right Posterior Cingulate & Cuneus | 2.25 | 0.03 | >.999 | 1.73 | 0.09 | >.999 | 1.65 | 0.10 | >.999 | 2.13 | 0.03 | >.999 | 1.96 | 0.05 | >.999 |
| Right Posterior Cingulate Gyrus | Left Anterior Cingulate Gyrus | 2.90 | <.001 | >.999 | 1.77 | 0.08 | >.999 | 2.59 | 0.01 | >.999 | 3.01 | <.001 | >.999 | 2.11 | 0.04 | >.999 |
| Right Posterior Cingulate Gyrus | Right Anterior Cingulate Gyrus | 1.45 | 0.15 | >.999 | 0.59 | 0.55 | >.999 | 1.46 | 0.15 | >.999 | 1.86 | 0.07 | >.999 | 1.53 | 0.13 | >.999 |
| Left Anterior Cingulate Gyrus | Left Insula | 1.17 | 0.24 | >.999 | 1.31 | 0.19 | >.999 | 2.61 | 0.01 | >.999 | 1.40 | 0.16 | >.999 | 0.65 | 0.52 | >.999 |
| Left Anterior Cingulate Gyrus | Right Insula | 1.93 | 0.06 | >.999 | 2.17 | 0.03 | >.999 | 2.35 | 0.02 | >.999 | 2.21 | 0.03 | >.999 | 1.53 | 0.13 | >.999 |
| Left Anterior Cingulate Gyrus | Right Anterior Cingulate Gyrus | -1.07 | 0.29 | >.999 | -1.47 | 0.14 | >.999 | -2.47 | 0.01 | >.999 | -0.66 | 0.51 | >.999 | -0.05 | 0.96 | >.999 |
| Left Anterior Cingulate Gyrus | Left Anterior Cingulate Gyrus | 1.92 | 0.06 | >.999 | 1.86 | 0.06 | >.999 | 2.29 | 0.02 | >.999 | 2.45 | 0.02 | >.999 | 1.78 | 0.08 | >.999 |
| Left Anterior Cingulate Gyrus | Right Anterior Cingulate Gyrus | 1.96 | 0.05 | >.999 | 1.86 | 0.06 | >.999 | 2.10 | 0.04 | >.999 | 2.44 | 0.02 | >.999 | 1.92 | 0.06 | >.999 |
| Left Anterior Cingulate Gyrus | Left Posterior Cingulate & Superior Transverse Temporal Gyrus | 2.27 | 0.02 | >.999 | 1.33 | 0.19 | >.999 | 1.96 | 0.05 | >.999 | 2.45 | 0.02 | >.999 | 2.08 | 0.04 | >.999 |
| Left Anterior Cingulate Gyrus | Right Posterior Cingulate & Superior Transverse Temporal Gyrus | 2.06 | 0.04 | >.999 | 0.96 | 0.34 | >.999 | 1.82 | 0.07 | >.999 | 2.03 | 0.04 | >.999 | 1.74 | 0.08 | >.999 |
| Left Anterior Cingulate Gyrus | Left Posterior Cingulate & Cuneus | 1.73 | 0.09 | >.999 | 1.16 | 0.25 | >.999 | 2.05 | 0.04 | >.999 | 2.69 | 0.01 | >.999 | 2.17 | 0.03 | >.999 |
| Left Anterior Cingulate Gyrus | Right Posterior Cingulate & Cuneus | 2.00 | 0.05 | >.999 | 0.93 | 0.36 | >.999 | 1.92 | 0.06 | >.999 | 2.62 | 0.01 | >.999 | 1.88 | 0.06 | >.999 |
| Left Anterior Cingulate Gyrus | Left Anterior Cingulate Gyrus | 0.65 | 0.52 | >.999 | 0.05 | 0.96 | >.999 | 0.36 | 0.72 | >.999 | 1.13 | 0.26 | >.999 | 0.47 | 0.64 | >.999 |
| Left Anterior Cingulate Gyrus | Right Anterior Cingulate Gyrus | -0.65 | 0.51 | >.999 | -0.86 | 0.39 | >.999 | -1.31 | 0.19 | >.999 | 0.51 | 0.61 | >.999 | 0.83 | 0.41 | >.999 |
| Left Anterior Cingulate Gyrus | Left Superior Temporal Gyrus & Subcallosal Gyrus-Entorhinal Area | 1.78 | 0.08 | >.999 | 1.67 | 0.10 | >.999 | 2.73 | 0.01 | >.999 | 2.29 | 0.02 | >.999 | 1.56 | 0.12 | >.999 |
| Left Anterior Cingulate Gyrus | Right Superior Temporal Gyrus & Subcallosal Gyrus-Entorhinal Area | 2.28 | 0.02 | >.999 | 1.82 | 0.07 | >.999 | 2.19 | 0.03 | >.999 | 2.63 | 0.01 | >.999 | 1.83 | 0.07 | >.999 |
| Left Anterior Cingulate Gyrus | Left Inferior Frontal and Extra Nuclear Gyrus of the Pre-Frontal Lobes | -0.15 | 0.88 | >.999 | 0.14 | 0.89 | >.999 | 1.29 | 0.20 | >.999 | -0.34 | 0.73 | >.999 | -0.99 | 0.33 | >.999 |
| Left Anterior Cingulate Gyrus | Right Inferior Frontal and Extra Nuclear Gyrus of the Pre-Frontal Lobes | 1.38 | 0.17 | >.999 | 1.36 | 0.18 | >.999 | 1.75 | 0.08 | >.999 | 1.52 | 0.13 | >.999 | 1.25 | 0.21 | >.999 |
| Left Anterior Cingulate Gyrus | Left Inferior Frontal and Extra Nuclear Gyrus of the Pre-Frontal Lobes | 1.05 | 0.30 | >.999 | 1.54 | 0.13 | >.999 | 2.28 | 0.02 | >.999 | 0.71 | 0.48 | >.999 | -0.30 | 0.76 | >.999 |
| Left Anterior Cingulate Gyrus | Right Inferior Frontal and Extra Nuclear Gyrus of the Pre-Frontal Lobes | 1.64 | 0.10 | >.999 | 1.92 | 0.06 | >.999 | 2.35 | 0.02 | >.999 | 2.24 | 0.03 | >.999 | 1.44 | 0.15 | >.999 |
| Left Anterior Cingulate Gyrus | Left Middle Frontal Gyrus | 0.81 | 0.42 | >.999 | 1.84 | 0.07 | >.999 | 2.48 | 0.01 | >.999 | 0.60 | 0.55 | >.999 | -0.27 | 0.79 | >.999 |
| Left Anterior Cingulate Gyrus | Right Middle Frontal Gyrus | 1.01 | 0.31 | >.999 | 1.41 | 0.16 | >.999 | 1.87 | 0.06 | >.999 | 1.63 | 0.10 | >.999 | 1.35 | 0.18 | >.999 |
| Left Anterior Cingulate Gyrus | Left Inferior Frontal | 1.90 | 0.06 | >.999 | 2.29 | 0.02 | >.999 | 3.33 | <.001 | 0.41 | 2.31 | 0.02 | >.999 | 1.43 | 0.15 | >.999 |
| Left Anterior Cingulate Gyrus | Right Inferior Frontal | 2.54 | 0.01 | >.999 | 2.82 | 0.01 | >.999 | 3.03 | <.001 | >.999 | 3.32 | <.001 | 0.43 | 2.36 | 0.02 | >.999 |
| Left Anterior Cingulate Gyrus | Left Parasubiculum hippocampal region | 1.63 | 0.11 | >.999 | 0.71 | 0.48 | >.999 | 1.73 | 0.09 | >.999 | 1.62 | 0.11 | >.999 | 1.31 | 0.19 | >.999 |
| Left Anterior Cingulate Gyrus | Right Parasubiculum hippocampal region | 1.85 | 0.07 | >.999 | 0.70 | 0.48 | >.999 | 1.69 | 0.09 | >.999 | 2.04 | 0.04 | >.999 | 1.92 | 0.06 | >.999 |
| Right Anterior Cingulate Gyrus | Left Insula | 1.06 | 0.29 | >.999 | 1.11 | 0.27 | >.999 | 2.43 | 0.02 | >.999 | 1.44 | 0.15 | >.999 | 1.00 | 0.32 | >.999 |
| Right Anterior Cingulate Gyrus | Right Insula | 2.42 | 0.02 | >.999 | 2.76 | 0.01 | >.999 | 2.55 | 0.01 | >.999 | 2.20 | 0.03 | >.999 | 1.40 | 0.16 | >.999 |
| Right Anterior Cingulate Gyrus | Left Anterior Cingulate Gyrus | 1.83 | 0.07 | >.999 | 1.95 | 0.05 | >.999 | 2.27 | 0.02 | >.999 | 2.92 | <.001 | >.999 | 2.63 | 0.01 | >.999 |
| Right Anterior Cingulate Gyrus | Right Anterior Cingulate Gyrus | 2.11 | 0.04 | >.999 | 2.22 | 0.03 | >.999 | 2.32 | 0.02 | >.999 | 2.84 | 0.01 | >.999 | 2.43 | 0.02 | >.999 |
| Right Anterior Cingulate Gyrus | Left Posterior Cingulate & Superior Transverse Temporal Gyrus | 1.65 | 0.10 | >.999 | 0.70 | 0.49 | >.999 | 1.56 | 0.12 | >.999 | 2.02 | 0.05 | >.999 | 2.18 | 0.03 | >.999 |
| Right Anterior Cingulate Gyrus | Right Posterior Cingulate & Superior Transverse Temporal Gyrus | 1.28 | 0.20 | >.999 | 0.39 | 0.70 | >.999 | 1.30 | 0.19 | >.999 | 1.16 | 0.25 | >.999 | 1.13 | 0.26 | >.999 |
| Right Anterior Cingulate Gyrus | Left Posterior Cingulate & Cuneus | 1.29 | 0.20 | >.999 | 0.60 | 0.55 | >.999 | 1.56 | 0.12 | >.999 | 2.28 | 0.02 | >.999 | 2.22 | 0.03 | >.999 |
| Right Anterior Cingulate Gyrus | Right Posterior Cingulate & Cuneus | 1.43 | 0.16 | >.999 | 0.57 | 0.57 | >.999 | 1.47 | 0.14 | >.999 | 1.61 | 0.11 | >.999 | 1.14 | 0.26 | >.999 |
| Right Anterior Cingulate Gyrus | Left Anterior Cingulate Gyrus | -0.37 | 0.72 | >.999 | -0.80 | 0.42 | >.999 | -0.71 | 0.48 | >.999 | 0.41 | 0.68 | >.999 | 0.17 | 0.87 | >.999 |
| Right Anterior Cingulate Gyrus | Right Anterior Cingulate Gyrus | 0.39 | 0.70 | >.999 | -0.25 | 0.80 | >.999 | -0.69 | 0.49 | >.999 | 1.33 | 0.18 | >.999 | 1.41 | 0.16 | >.999 |
| Right Anterior Cingulate Gyrus | Left Superior Temporal Gyrus & Subcallosal Gyrus-Entorhinal Area | 1.53 | 0.13 | >.999 | 1.46 | 0.15 | >.999 | 2.51 | 0.01 | >.999 | 2.42 | 0.02 | >.999 | 2.04 | 0.04 | >.999 |
| Right Anterior Cingulate Gyrus | Right Superior Temporal Gyrus & Subcallosal Gyrus-Entorhinal Area | 2.27 | 0.02 | >.999 | 1.96 | 0.05 | >.999 | 2.14 | 0.03 | >.999 | 2.46 | 0.02 | >.999 | 1.81 | 0.07 | >.999 |
| Right Anterior Cingulate Gyrus | Left Inferior Frontal and Extra Nuclear Gyrus of the Pre-Frontal Lobes | -0.15 | 0.88 | >.999 | 0.04 | 0.97 | >.999 | 1.37 | 0.17 | >.999 | 0.23 | 0.82 | >.999 | 0.45 | 0.65 | >.999 |
| Right Anterior Cingulate Gyrus | Right Inferior Frontal and Extra Nuclear Gyrus of the Pre-Frontal Lobes | 1.77 | 0.08 | >.999 | 1.89 | 0.06 | >.999 | 1.70 | 0.09 | >.999 | 2.13 | 0.03 | >.999 | 1.91 | 0.06 | >.999 |
| Right Anterior Cingulate Gyrus | Left Inferior Frontal and Extra Nuclear Gyrus of the Pre-Frontal Lobes | 1.14 | 0.25 | >.999 | 1.49 | 0.14 | >.999 | 2.23 | 0.03 | >.999 | 1.46 | 0.15 | >.999 | 1.10 | 0.27 | >.999 |
| Right Anterior Cingulate Gyrus | Right Inferior Frontal and Extra Nuclear Gyrus of the Pre-Frontal Lobes | 2.13 | 0.03 | >.999 | 2.62 | 0.01 | >.999 | 2.41 | 0.02 | >.999 | 2.38 | 0.02 | >.999 | 1.59 | 0.11 | >.999 |
| Right Anterior Cingulate Gyrus | Left Middle Frontal Gyrus | 0.74 | 0.46 | >.999 | 1.75 | 0.08 | >.999 | 2.50 | 0.01 | >.999 | 1.24 | 0.22 | >.999 | 1.04 | 0.30 | >.999 |
| Right Anterior Cingulate Gyrus | Right Middle Frontal Gyrus | 1.57 | 0.12 | >.999 | 2.06 | 0.04 | >.999 | 1.82 | 0.07 | >.999 | 1.77 | 0.08 | >.999 | 1.38 | 0.17 | >.999 |
| Right Anterior Cingulate Gyrus | Left Inferior Frontal | 1.74 | 0.08 | >.999 | 1.90 | 0.06 | >.999 | 2.98 | <.001 | >.999 | 2.22 | 0.03 | >.999 | 1.55 | 0.12 | >.999 |
| Right Anterior Cingulate Gyrus | Right Inferior Frontal | 3.17 | <.001 | 0.72 | 3.57 | <.001 | 0.18 | 3.46 | <.001 | 0.27 | 3.38 | <.001 | 0.35 | 2.21 | 0.03 | >.999 |
| Right Anterior Cingulate Gyrus | Left Parasubiculum hippocampal region | 0.98 | 0.33 | >.999 | 0.23 | 0.82 | >.999 | 1.11 | 0.27 | >.999 | 1.19 | 0.24 | >.999 | 1.42 | 0.16 | >.999 |
| Right Anterior Cingulate Gyrus | Right Parasubiculum hippocampal region | 1.26 | 0.21 | >.999 | 0.31 | 0.76 | >.999 | 1.11 | 0.27 | >.999 | 1.32 | 0.19 | >.999 | 1.66 | 0.10 | >.999 |
| Left Anterior Cingulate Gyrus | Left Insula | -0.02 | 0.99 | >.999 | -0.06 | 0.95 | >.999 | 0.97 | 0.33 | >.999 | -1.18 | 0.24 | >.999 | -2.02 | 0.04 | >.999 |
| Left Anterior Cingulate Gyrus | Right Insula | 3.11 | <.001 | 0.87 | 3.70 | <.001 | 0.12 | 3.78 | <.001 | 0.09 | 3.89 | <.001 | 0.06 | 3.49 | <.001 | 0.25 |
| Left Anterior Cingulate Gyrus | Right Anterior Cingulate Gyrus | 2.50 | 0.01 | >.999 | 2.25 | 0.03 | >.999 | 1.15 | 0.25 | >.999 | 3.83 | <.001 | 0.07 | 3.91 | <.001 | 0.06 |
| Left Anterior Cingulate Gyrus | Left Posterior Cingulate & Superior Transverse Temporal Gyrus | 2.47 | 0.01 | >.999 | 2.46 | 0.01 | >.999 | 2.59 | 0.01 | >.999 | 3.02 | <.001 | >.999 | 2.94 | <.001 | >.999 |
| Left Anterior Cingulate Gyrus | Right Posterior Cingulate & Superior Transverse Temporal Gyrus | 2.21 | 0.03 | >.999 | 2.48 | 0.01 | >.999 | 2.61 | 0.01 | >.999 | 2.49 | 0.01 | >.999 | 1.78 | 0.08 | >.999 |
| Left Anterior Cingulate Gyrus | Left Posterior Cingulate & Cuneus | 1.65 | 0.10 | >.999 | 1.27 | 0.21 | >.999 | 1.55 | 0.12 | >.999 | 1.10 | 0.27 | >.999 | 0.71 | 0.48 | >.999 |
| Left Anterior Cingulate Gyrus | Right Posterior Cingulate & Cuneus | 1.83 | 0.07 | >.999 | 2.09 | 0.04 | >.999 | 2.49 | 0.01 | >.999 | 2.06 | 0.04 | >.999 | 1.07 | 0.29 | >.999 |
| Left Anterior Cingulate Gyrus | Left Anterior Cingulate Gyrus | 0.04 | 0.97 | >.999 | -0.23 | 0.82 | >.999 | 1.08 | 0.28 | >.999 | -0.20 | 0.84 | >.999 | -1.11 | 0.27 | >.999 |
| Left Anterior Cingulate Gyrus | Right Anterior Cingulate Gyrus | -0.17 | 0.86 | >.999 | -0.33 | 0.74 | >.999 | 0.34 | 0.73 | >.999 | 0.64 | 0.52 | >.999 | 0.56 | 0.58 | >.999 |
| Left Anterior Cingulate Gyrus | Left Superior Temporal Gyrus & Subcallosal Gyrus-Entorhinal Area | 1.90 | 0.06 | >.999 | 1.52 | 0.13 | >.999 | 1.55 | 0.12 | >.999 | 1.82 | 0.07 | >.999 | 1.50 | 0.14 | >.999 |
| Left Anterior Cingulate Gyrus | Right Superior Temporal Gyrus & Subcallosal Gyrus-Entorhinal Area | 3.36 | <.001 | 0.39 | 3.62 | <.001 | 0.16 | 3.48 | <.001 | 0.26 | 4.01* | <.001 | 0.04 | 3.59 | <.001 | 0.18 |
| Left Anterior Cingulate Gyrus | Left Inferior Frontal and Extra Nuclear Gyrus of the Pre-Frontal Lobes | 0.24 | 0.81 | >.999 | 0.28 | 0.78 | >.999 | 1.58 | 0.12 | >.999 | -0.38 | 0.70 | >.999 | -1.20 | 0.23 | >.999 |
| Left Anterior Cingulate Gyrus | Right Inferior Frontal and Extra Nuclear Gyrus of the Pre-Frontal Lobes | 2.47 | 0.01 | >.999 | 2.57 | 0.01 | >.999 | 3.01 | <.001 | >.999 | 3.22 | <.001 | 0.59 | 2.98 | <.001 | >.999 |
| Left Anterior Cingulate Gyrus | Left Inferior Frontal and Extra Nuclear Gyrus of the Pre-Frontal Lobes | 0.39 | 0.70 | >.999 | 0.95 | 0.34 | >.999 | 1.73 | 0.09 | >.999 | 0.84 | 0.40 | >.999 | 0.16 | 0.88 | >.999 |
| Left Anterior Cingulate Gyrus | Right Inferior Frontal and Extra Nuclear Gyrus of the Pre-Frontal Lobes | 2.18 | 0.03 | >.999 | 2.63 | 0.01 | >.999 | 2.98 | <.001 | >.999 | 3.21 | <.001 | 0.60 | 2.93 | <.001 | >.999 |
| Left Anterior Cingulate Gyrus | Left Middle Frontal Gyrus | 0.57 | 0.57 | >.999 | 1.57 | 0.12 | >.999 | 2.46 | 0.02 | >.999 | 0.68 | 0.50 | >.999 | 0.03 | 0.98 | >.999 |
| Left Anterior Cingulate Gyrus | Right Middle Frontal Gyrus | 0.95 | 0.34 | >.999 | 1.41 | 0.16 | >.999 | 1.92 | 0.06 | >.999 | 1.75 | 0.08 | >.999 | 2.24 | 0.03 | >.999 |
| Left Anterior Cingulate Gyrus | Left Inferior Frontal | 0.55 | 0.59 | >.999 | 0.75 | 0.46 | >.999 | 1.66 | 0.10 | >.999 | -0.68 | 0.50 | >.999 | -1.72 | 0.09 | >.999 |
| Left Anterior Cingulate Gyrus | Right Inferior Frontal | 2.96 | <.001 | >.999 | 3.84 | <.001 | 0.07 | 3.93 | <.001 | 0.05 | 3.90 | <.001 | 0.06 | 3.71 | <.001 | 0.11 |
| Left Anterior Cingulate Gyrus | Left Parasubiculum hippocampal region | 3.08 | <.001 | 0.94 | 2.58 | 0.01 | >.999 | 2.55 | 0.01 | >.999 | 2.75 | 0.01 | >.999 | 2.93 | <.001 | >.999 |
| Left Anterior Cingulate Gyrus | Right Parasubiculum hippocampal region | 3.30 | <.001 | 0.46 | 3.34 | <.001 | 0.40 | 3.26 | <.001 | 0.51 | 3.28 | <.001 | 0.48 | 2.91 | <.001 | >.999 |
| Right Anterior Cingulate Gyrus | Left Insula | 0.83 | 0.41 | >.999 | 0.83 | 0.41 | >.999 | 1.73 | 0.09 | >.999 | 0.42 | 0.67 | >.999 | 0.06 | 0.95 | >.999 |
| Right Anterior Cingulate Gyrus | Right Insula | 2.97 | <.001 | >.999 | 3.55 | <.001 | 0.20 | 3.61 | <.001 | 0.16 | 3.65 | <.001 | 0.14 | 3.11 | <.001 | 0.84 |
| Right Anterior Cingulate Gyrus | Left Posterior Cingulate & Superior Transverse Temporal Gyrus | 2.21 | 0.03 | >.999 | 2.28 | 0.02 | >.999 | 2.50 | 0.01 | >.999 | 2.76 | 0.01 | >.999 | 2.87 | <.001 | >.999 |
| Right Anterior Cingulate Gyrus | Right Posterior Cingulate & Superior Transverse Temporal Gyrus | 1.98 | 0.05 | >.999 | 2.26 | 0.03 | >.999 | 2.37 | 0.02 | >.999 | 2.09 | 0.04 | >.999 | 1.52 | 0.13 | >.999 |
| Right Anterior Cingulate Gyrus | Left Posterior Cingulate & Cuneus | 1.55 | 0.12 | >.999 | 1.26 | 0.21 | >.999 | 1.68 | 0.10 | >.999 | 1.23 | 0.22 | >.999 | 1.10 | 0.27 | >.999 |
| Right Anterior Cingulate Gyrus | Right Posterior Cingulate & Cuneus | 1.54 | 0.12 | >.999 | 1.80 | 0.07 | >.999 | 2.06 | 0.04 | >.999 | 1.60 | 0.11 | >.999 | 0.73 | 0.47 | >.999 |
| Right Anterior Cingulate Gyrus | Left Anterior Cingulate Gyrus | 0.31 | 0.76 | >.999 | 0.07 | 0.94 | >.999 | 1.27 | 0.21 | >.999 | 0.31 | 0.76 | >.999 | -0.52 | 0.60 | >.999 |
| Right Anterior Cingulate Gyrus | Right Anterior Cingulate Gyrus | -0.27 | 0.79 | >.999 | -0.49 | 0.63 | >.999 | -0.02 | 0.98 | >.999 | 0.14 | 0.89 | >.999 | -0.01 | 0.99 | >.999 |
| Right Anterior Cingulate Gyrus | Left Superior Temporal Gyrus & Subcallosal Gyrus-Entorhinal Area | 2.11 | 0.04 | >.999 | 2.02 | 0.05 | >.999 | 2.17 | 0.03 | >.999 | 2.47 | 0.01 | >.999 | 2.49 | 0.01 | >.999 |
| Right Anterior Cingulate Gyrus | Right Superior Temporal Gyrus & Subcallosal Gyrus-Entorhinal Area | 2.89 | <.001 | >.999 | 3.22 | <.001 | 0.59 | 3.24 | <.001 | 0.55 | 3.65 | <.001 | 0.14 | 3.24 | <.001 | 0.56 |
| Right Anterior Cingulate Gyrus | Left Inferior Frontal and Extra Nuclear Gyrus of the Pre-Frontal Lobes | 0.68 | 0.49 | >.999 | 0.83 | 0.41 | >.999 | 2.08 | 0.04 | >.999 | 1.07 | 0.29 | >.999 | 0.68 | 0.50 | >.999 |
| Right Anterior Cingulate Gyrus | Right Inferior Frontal and Extra Nuclear Gyrus of the Pre-Frontal Lobes | 2.23 | 0.03 | >.999 | 2.41 | 0.02 | >.999 | 2.87 | <.001 | >.999 | 2.96 | <.001 | >.999 | 2.80 | 0.01 | >.999 |
| Right Anterior Cingulate Gyrus | Left Inferior Frontal and Extra Nuclear Gyrus of the Pre-Frontal Lobes | 1.11 | 0.27 | >.999 | 1.70 | 0.09 | >.999 | 2.25 | 0.03 | >.999 | 2.37 | 0.02 | >.999 | 1.77 | 0.08 | >.999 |
| Right Anterior Cingulate Gyrus | Right Inferior Frontal and Extra Nuclear Gyrus of the Pre-Frontal Lobes | 1.82 | 0.07 | >.999 | 2.37 | 0.02 | >.999 | 2.71 | 0.01 | >.999 | 2.58 | 0.01 | >.999 | 2.32 | 0.02 | >.999 |
| Right Anterior Cingulate Gyrus | Left Middle Frontal Gyrus | 1.23 | 0.22 | >.999 | 2.33 | 0.02 | >.999 | 3.02 | <.001 | >.999 | 2.32 | 0.02 | >.999 | 1.57 | 0.12 | >.999 |
| Right Anterior Cingulate Gyrus | Right Middle Frontal Gyrus | 0.23 | 0.82 | >.999 | 0.76 | 0.45 | >.999 | 1.31 | 0.19 | >.999 | 0.45 | 0.66 | >.999 | 0.85 | 0.40 | >.999 |
| Right Anterior Cingulate Gyrus | Left Inferior Frontal | 1.36 | 0.18 | >.999 | 1.53 | 0.13 | >.999 | 2.26 | 0.03 | >.999 | 1.06 | 0.29 | >.999 | 0.45 | 0.65 | >.999 |
| Right Anterior Cingulate Gyrus | Right Inferior Frontal | 2.63 | 0.01 | >.999 | 3.56 | <.001 | 0.19 | 3.58 | <.001 | 0.18 | 3.29 | <.001 | 0.47 | 2.98 | <.001 | >.999 |
| Right Anterior Cingulate Gyrus | Left Parasubiculum hippocampal region | 2.78 | 0.01 | >.999 | 2.39 | 0.02 | >.999 | 2.39 | 0.02 | >.999 | 2.75 | 0.01 | >.999 | 3.34 | <.001 | 0.41 |
| Right Anterior Cingulate Gyrus | Right Parasubiculum hippocampal region | 3.06 | <.001 | 0.98 | 3.10 | <.001 | 0.86 | 2.80 | 0.01 | >.999 | 2.85 | 0.01 | >.999 | 2.57 | 0.01 | >.999 |
| Left Posterior Cingulate & Superior Transverse Temporal Gyrus | Left Insula | 2.25 | 0.03 | >.999 | 2.32 | 0.02 | >.999 | 2.53 | 0.01 | >.999 | 1.59 | 0.11 | >.999 | 0.77 | 0.44 | >.999 |
| Left Posterior Cingulate & Superior Transverse Temporal Gyrus | Right Insula | 1.61 | 0.11 | >.999 | 2.17 | 0.03 | >.999 | 2.39 | 0.02 | >.999 | 1.99 | 0.05 | >.999 | 1.26 | 0.21 | >.999 |
| Left Posterior Cingulate & Superior Transverse Temporal Gyrus | Right Posterior Cingulate & Superior Transverse Temporal Gyrus | 1.15 | 0.25 | >.999 | 0.75 | 0.46 | >.999 | 1.20 | 0.23 | >.999 | 1.60 | 0.11 | >.999 | 1.26 | 0.21 | >.999 |
| Left Posterior Cingulate & Superior Transverse Temporal Gyrus | Left Posterior Cingulate & Cuneus | 1.57 | 0.12 | >.999 | 1.69 | 0.09 | >.999 | 1.76 | 0.08 | >.999 | 2.56 | 0.01 | >.999 | 2.60 | 0.01 | >.999 |
| Left Posterior Cingulate & Superior Transverse Temporal Gyrus | Right Posterior Cingulate & Cuneus | 1.16 | 0.25 | >.999 | >.999 | 0.32 | >.999 | 1.66 | 0.10 | >.999 | 2.39 | 0.02 | >.999 | 2.47 | 0.01 | >.999 |
| Left Posterior Cingulate & Superior Transverse Temporal Gyrus | Left Anterior Cingulate Gyrus | 2.38 | 0.02 | >.999 | 1.59 | 0.11 | >.999 | 2.50 | 0.01 | >.999 | 2.64 | 0.01 | >.999 | 1.26 | 0.21 | >.999 |
| Left Posterior Cingulate & Superior Transverse Temporal Gyrus | Right Anterior Cingulate Gyrus | 1.14 | 0.26 | >.999 | 0.20 | 0.84 | >.999 | 1.37 | 0.17 | >.999 | 2.20 | 0.03 | >.999 | 1.76 | 0.08 | >.999 |
| Right Posterior Cingulate & Superior Transverse Temporal Gyrus | Left Insula | 1.81 | 0.07 | >.999 | 2.16 | 0.03 | >.999 | 2.39 | 0.02 | >.999 | 1.25 | 0.21 | >.999 | 0.22 | 0.82 | >.999 |
| Right Posterior Cingulate & Superior Transverse Temporal Gyrus | Right Insula | 1.93 | 0.06 | >.999 | 2.54 | 0.01 | >.999 | 2.53 | 0.01 | >.999 | 1.83 | 0.07 | >.999 | 0.98 | 0.33 | >.999 |
| Right Posterior Cingulate & Superior Transverse Temporal Gyrus | Left Posterior Cingulate & Cuneus | 1.12 | 0.26 | >.999 | 0.97 | 0.33 | >.999 | 1.49 | 0.14 | >.999 | 1.72 | 0.09 | >.999 | 1.53 | 0.13 | >.999 |
| Right Posterior Cingulate & Superior Transverse Temporal Gyrus | Right Posterior Cingulate & Cuneus | 1.94 | 0.05 | >.999 | 1.41 | 0.16 | >.999 | 1.02 | 0.31 | >.999 | 1.67 | 0.10 | >.999 | 1.60 | 0.11 | >.999 |
| Right Posterior Cingulate & Superior Transverse Temporal Gyrus | Left Anterior Cingulate Gyrus | 2.41 | 0.02 | >.999 | 1.24 | 0.22 | >.999 | 2.33 | 0.02 | >.999 | 2.66 | 0.01 | >.999 | 1.51 | 0.13 | >.999 |
| Right Posterior Cingulate & Superior Transverse Temporal Gyrus | Right Anterior Cingulate Gyrus | 0.78 | 0.44 | >.999 | 0.01 | 0.99 | >.999 | 1.27 | 0.20 | >.999 | 1.13 | 0.26 | >.999 | 0.55 | 0.58 | >.999 |
| Left Posterior Cingulate & Cuneus | Left Insula | 1.62 | 0.11 | >.999 | 1.14 | 0.26 | >.999 | 1.38 | 0.17 | >.999 | -0.38 | 0.71 | >.999 | -1.14 | 0.25 | >.999 |
| Left Posterior Cingulate & Cuneus | Right Insula | 1.61 | 0.11 | >.999 | 1.81 | 0.07 | >.999 | 2.04 | 0.04 | >.999 | 1.25 | 0.22 | >.999 | 0.63 | 0.53 | >.999 |
| Left Posterior Cingulate & Cuneus | Right Posterior Cingulate & Cuneus | 0.80 | 0.43 | >.999 | 0.55 | 0.59 | >.999 | 1.58 | 0.12 | >.999 | 1.75 | 0.08 | >.999 | 1.75 | 0.08 | >.999 |
| Left Posterior Cingulate & Cuneus | Left Anterior Cingulate Gyrus | 1.72 | 0.09 | >.999 | 1.47 | 0.14 | >.999 | 2.48 | 0.01 | >.999 | 2.50 | 0.01 | >.999 | 1.19 | 0.24 | >.999 |
| Left Posterior Cingulate & Cuneus | Right Anterior Cingulate Gyrus | 1.01 | 0.31 | >.999 | 0.22 | 0.82 | >.999 | 1.12 | 0.26 | >.999 | 1.83 | 0.07 | >.999 | 1.29 | 0.20 | >.999 |
| Right Posterior Cingulate & Cuneus | Left Insula | 1.32 | 0.19 | >.999 | 1.36 | 0.18 | >.999 | 1.83 | 0.07 | >.999 | 0.35 | 0.73 | >.999 | -0.37 | 0.71 | >.999 |
| Right Posterior Cingulate & Cuneus | Right Insula | 1.80 | 0.07 | >.999 | 2.16 | 0.03 | >.999 | 2.18 | 0.03 | >.999 | 1.46 | 0.15 | >.999 | 0.75 | 0.45 | >.999 |
| Right Posterior Cingulate & Cuneus | Left Anterior Cingulate Gyrus | 2.33 | 0.02 | >.999 | 1.21 | 0.23 | >.999 | 2.21 | 0.03 | >.999 | 2.77 | 0.01 | >.999 | 1.45 | 0.15 | >.999 |
| Right Posterior Cingulate & Cuneus | Right Anterior Cingulate Gyrus | 0.71 | 0.48 | >.999 | -0.09 | 0.93 | >.999 | 1.04 | 0.30 | >.999 | 0.78 | 0.44 | >.999 | 0.23 | 0.82 | >.999 |
| Left Anterior Cingulate Gyrus | Left Insula | 0.52 | 0.61 | >.999 | 0.84 | 0.40 | >.999 | 2.43 | 0.02 | >.999 | 1.04 | 0.30 | >.999 | 0.34 | 0.73 | >.999 |
| Left Anterior Cingulate Gyrus | Right Insula | 0.95 | 0.34 | >.999 | 1.06 | 0.29 | >.999 | 1.93 | 0.06 | >.999 | 0.68 | 0.50 | >.999 | -0.28 | 0.78 | >.999 |
| Left Anterior Cingulate Gyrus | Right Anterior Cingulate Gyrus | -1.40 | 0.16 | >.999 | -1.18 | 0.24 | >.999 | -1.06 | 0.29 | >.999 | -0.15 | 0.88 | >.999 | 0.02 | 0.98 | >.999 |
| Left Anterior Cingulate Gyrus | Left Superior Temporal Gyrus & Subcallosal Gyrus-Entorhinal Area | 1.05 | 0.30 | >.999 | 1.10 | 0.27 | >.999 | 2.68 | 0.01 | >.999 | 1.33 | 0.19 | >.999 | 0.25 | 0.81 | >.999 |
| Left Anterior Cingulate Gyrus | Right Superior Temporal Gyrus & Subcallosal Gyrus-Entorhinal Area | 1.62 | 0.11 | >.999 | 1.07 | 0.29 | >.999 | 2.01 | 0.05 | >.999 | 1.68 | 0.10 | >.999 | 0.27 | 0.79 | >.999 |
| Left Anterior Cingulate Gyrus | Left Inferior Frontal and Extra Nuclear Gyrus of the Pre-Frontal Lobes | 0.74 | 0.46 | >.999 | 1.17 | 0.24 | >.999 | 2.25 | 0.03 | >.999 | 1.05 | 0.30 | >.999 | 0.48 | 0.63 | >.999 |
| Left Anterior Cingulate Gyrus | Right Inferior Frontal and Extra Nuclear Gyrus of the Pre-Frontal Lobes | 0.61 | 0.54 | >.999 | 0.90 | 0.37 | >.999 | 2.01 | 0.05 | >.999 | 0.26 | 0.79 | >.999 | -0.60 | 0.55 | >.999 |
| Left Anterior Cingulate Gyrus | Left Inferior Frontal and Extra Nuclear Gyrus of the Pre-Frontal Lobes | 0.29 | 0.77 | >.999 | 1.01 | 0.32 | >.999 | 2.10 | 0.04 | >.999 | 0.62 | 0.54 | >.999 | -0.16 | 0.87 | >.999 |
| Left Anterior Cingulate Gyrus | Right Inferior Frontal and Extra Nuclear Gyrus of the Pre-Frontal Lobes | 0.91 | 0.37 | >.999 | 1.20 | 0.23 | >.999 | 2.25 | 0.03 | >.999 | 0.84 | 0.40 | >.999 | -0.24 | 0.81 | >.999 |
| Left Anterior Cingulate Gyrus | Left Middle Frontal Gyrus | 0.13 | 0.89 | >.999 | 1.58 | 0.12 | >.999 | 2.35 | 0.02 | >.999 | 0.43 | 0.67 | >.999 | -0.21 | 0.83 | >.999 |
| Left Anterior Cingulate Gyrus | Right Middle Frontal Gyrus | 0.77 | 0.44 | >.999 | 1.51 | 0.13 | >.999 | 2.68 | 0.01 | >.999 | 1.63 | 0.10 | >.999 | 1.12 | 0.26 | >.999 |
| Left Anterior Cingulate Gyrus | Left Inferior Frontal | 0.35 | 0.73 | >.999 | 0.94 | 0.35 | >.999 | 2.42 | 0.02 | >.999 | 0.84 | 0.40 | >.999 | 0.05 | 0.96 | >.999 |
| Left Anterior Cingulate Gyrus | Right Inferior Frontal | 1.44 | 0.15 | >.999 | 1.75 | 0.08 | >.999 | 2.64 | 0.01 | >.999 | 1.82 | 0.07 | >.999 | 0.78 | 0.44 | >.999 |
| Left Anterior Cingulate Gyrus | Left Parasubiculum hippocampal region | 1.48 | 0.14 | >.999 | 0.81 | 0.42 | >.999 | 2.21 | 0.03 | >.999 | 1.75 | 0.08 | >.999 | 0.83 | 0.41 | >.999 |
| Left Anterior Cingulate Gyrus | Right Parasubiculum hippocampal region | 1.66 | 0.10 | >.999 | 0.71 | 0.48 | >.999 | 2.07 | 0.04 | >.999 | 2.09 | 0.04 | >.999 | 1.24 | 0.22 | >.999 |
| Right Anterior Cingulate Gyrus | Left Insula | 0.45 | 0.66 | >.999 | 0.44 | 0.66 | >.999 | 1.86 | 0.07 | >.999 | 1.40 | 0.16 | >.999 | 0.84 | 0.40 | >.999 |
| Right Anterior Cingulate Gyrus | Right Insula | 0.74 | 0.46 | >.999 | 0.82 | 0.41 | >.999 | 0.85 | 0.40 | >.999 | -0.34 | 0.74 | >.999 | -1.07 | 0.29 | >.999 |
| Right Anterior Cingulate Gyrus | Left Superior Temporal Gyrus & Subcallosal Gyrus-Entorhinal Area | 0.70 | 0.49 | >.999 | 0.50 | 0.62 | >.999 | 1.74 | 0.08 | >.999 | 1.67 | 0.10 | >.999 | 0.95 | 0.34 | >.999 |
| Right Anterior Cingulate Gyrus | Right Superior Temporal Gyrus & Subcallosal Gyrus-Entorhinal Area | 0.65 | 0.51 | >.999 | 0.17 | 0.87 | >.999 | 0.77 | 0.44 | >.999 | 0.70 | 0.48 | >.999 | 0.33 | 0.74 | >.999 |
| Right Anterior Cingulate Gyrus | Left Inferior Frontal and Extra Nuclear Gyrus of the Pre-Frontal Lobes | -0.08 | 0.94 | >.999 | 0.29 | 0.77 | >.999 | 1.81 | 0.07 | >.999 | 1.39 | 0.17 | >.999 | 1.46 | 0.15 | >.999 |
| Right Anterior Cingulate Gyrus | Right Inferior Frontal and Extra Nuclear Gyrus of the Pre-Frontal Lobes | 1.60 | 0.11 | >.999 | 1.59 | 0.11 | >.999 | 1.95 | 0.05 | >.999 | 1.73 | 0.09 | >.999 | 1.28 | 0.20 | >.999 |
| Right Anterior Cingulate Gyrus | Left Inferior Frontal and Extra Nuclear Gyrus of the Pre-Frontal Lobes | 0.96 | 0.34 | >.999 | 1.28 | 0.20 | >.999 | 2.02 | 0.04 | >.999 | 1.93 | 0.05 | >.999 | 1.52 | 0.13 | >.999 |
| Right Anterior Cingulate Gyrus | Right Inferior Frontal and Extra Nuclear Gyrus of the Pre-Frontal Lobes | 0.89 | 0.37 | >.999 | 1.39 | 0.17 | >.999 | 1.31 | 0.19 | >.999 | 0.38 | 0.71 | >.999 | -0.37 | 0.71 | >.999 |
| Right Anterior Cingulate Gyrus | Left Middle Frontal Gyrus | 0.55 | 0.58 | >.999 | 1.54 | 0.12 | >.999 | 2.39 | 0.02 | >.999 | 1.51 | 0.13 | >.999 | 1.42 | 0.16 | >.999 |
| Right Anterior Cingulate Gyrus | Right Middle Frontal Gyrus | 1.30 | 0.20 | >.999 | 2.11 | 0.04 | >.999 | 2.03 | 0.04 | >.999 | 2.26 | 0.03 | >.999 | 1.82 | 0.07 | >.999 |
| Right Anterior Cingulate Gyrus | Left Inferior Frontal | 1.00 | 0.32 | >.999 | 0.96 | 0.34 | >.999 | 2.11 | 0.04 | >.999 | 1.71 | 0.09 | >.999 | 1.11 | 0.27 | >.999 |
| Right Anterior Cingulate Gyrus | Right Inferior Frontal | 1.10 | 0.27 | >.999 | 1.35 | 0.18 | >.999 | 1.23 | 0.22 | >.999 | 0.85 | 0.40 | >.999 | -0.06 | 0.95 | >.999 |
| Right Anterior Cingulate Gyrus | Left Parasubiculum hippocampal region | 0.32 | 0.75 | >.999 | -0.42 | 0.67 | >.999 | 0.75 | 0.46 | >.999 | 1.08 | 0.28 | >.999 | 1.10 | 0.27 | >.999 |
| Right Anterior Cingulate Gyrus | Right Parasubiculum hippocampal region | 0.27 | 0.79 | >.999 | -0.64 | 0.53 | >.999 | 0.55 | 0.59 | >.999 | 0.75 | 0.45 | >.999 | 0.91 | 0.36 | >.999 |
| Left Superior Temporal Gyrus & Subcallosal Gyrus-Entorhinal Area | Left Insula | -0.11 | 0.91 | >.999 | -0.20 | 0.84 | >.999 | 0.52 | 0.61 | >.999 | -2.27 | 0.02 | >.999 | -3.18 | <.001 | 0.68 |
| Left Superior Temporal Gyrus & Subcallosal Gyrus-Entorhinal Area | Right Insula | 2.59 | 0.01 | >.999 | 3.25 | <.001 | 0.54 | 3.27 | <.001 | 0.50 | 2.99 | <.001 | >.999 | 2.65 | 0.01 | >.999 |
| Left Superior Temporal Gyrus & Subcallosal Gyrus-Entorhinal Area | Right Superior Temporal Gyrus & Subcallosal Gyrus-Entorhinal Area | 2.41 | 0.02 | >.999 | 2.84 | 0.01 | >.999 | 2.61 | 0.01 | >.999 | 2.21 | 0.03 | >.999 | 1.95 | 0.05 | >.999 |
| Left Superior Temporal Gyrus & Subcallosal Gyrus-Entorhinal Area | Left Inferior Frontal and Extra Nuclear Gyrus of the Pre-Frontal Lobes | -0.13 | 0.90 | >.999 | 0.07 | 0.95 | >.999 | 1.26 | 0.21 | >.999 | -2.10 | 0.04 | >.999 | -2.96 | <.001 | >.999 |
| Left Superior Temporal Gyrus & Subcallosal Gyrus-Entorhinal Area | Right Inferior Frontal and Extra Nuclear Gyrus of the Pre-Frontal Lobes | 2.17 | 0.03 | >.999 | 2.24 | 0.03 | >.999 | 2.68 | 0.01 | >.999 | 2.08 | 0.04 | >.999 | 1.89 | 0.06 | >.999 |
| Left Superior Temporal Gyrus & Subcallosal Gyrus-Entorhinal Area | Left Inferior Frontal and Extra Nuclear Gyrus of the Pre-Frontal Lobes | 0.97 | 0.33 | >.999 | 1.60 | 0.11 | >.999 | 2.10 | 0.04 | >.999 | 0.45 | 0.66 | >.999 | -0.58 | 0.56 | >.999 |
| Left Superior Temporal Gyrus & Subcallosal Gyrus-Entorhinal Area | Right Inferior Frontal and Extra Nuclear Gyrus of the Pre-Frontal Lobes | 2.06 | 0.04 | >.999 | 2.35 | 0.02 | >.999 | 2.74 | 0.01 | >.999 | 2.67 | 0.01 | >.999 | 2.46 | 0.02 | >.999 |
| Left Superior Temporal Gyrus & Subcallosal Gyrus-Entorhinal Area | Left Middle Frontal Gyrus | 1.46 | 0.15 | >.999 | 2.21 | 0.03 | >.999 | 2.97 | <.001 | >.999 | 0.86 | 0.39 | >.999 | 0.05 | 0.96 | >.999 |
| Left Superior Temporal Gyrus & Subcallosal Gyrus-Entorhinal Area | Right Middle Frontal Gyrus | 1.51 | 0.13 | >.999 | 1.55 | 0.12 | >.999 | 2.12 | 0.04 | >.999 | 1.96 | 0.05 | >.999 | 1.82 | 0.07 | >.999 |
| Left Superior Temporal Gyrus & Subcallosal Gyrus-Entorhinal Area | Left Inferior Frontal | 1.22 | 0.22 | >.999 | 1.20 | 0.23 | >.999 | 1.70 | 0.09 | >.999 | -1.07 | 0.29 | >.999 | -2.46 | 0.02 | >.999 |
| Left Superior Temporal Gyrus & Subcallosal Gyrus-Entorhinal Area | Right Inferior Frontal | 2.69 | 0.01 | >.999 | 3.34 | <.001 | 0.41 | 3.36 | <.001 | 0.38 | 3.24 | <.001 | 0.55 | 3.07 | <.001 | 0.96 |
| Left Superior Temporal Gyrus & Subcallosal Gyrus-Entorhinal Area | Left Parasubiculum hippocampal region | 3.06 | <.001 | >.999 | 2.79 | 0.01 | >.999 | 3.07 | <.001 | 0.90 | 2.50 | 0.01 | >.999 | 2.20 | 0.03 | >.999 |
| Left Superior Temporal Gyrus & Subcallosal Gyrus-Entorhinal Area | Right Parasubiculum hippocampal region | 2.52 | 0.01 | >.999 | 2.52 | 0.01 | >.999 | 2.95 | <.001 | >.999 | 2.46 | 0.02 | >.999 | 2.12 | 0.04 | >.999 |
| Right Superior Temporal Gyrus & Subcallosal Gyrus-Entorhinal Area | Left Insula | 1.93 | 0.06 | >.999 | 1.88 | 0.06 | >.999 | 1.95 | 0.05 | >.999 | 1.22 | 0.23 | >.999 | 0.68 | 0.50 | >.999 |
| Right Superior Temporal Gyrus & Subcallosal Gyrus-Entorhinal Area | Right Insula | 1.37 | 0.17 | >.999 | 2.13 | 0.03 | >.999 | 2.07 | 0.04 | >.999 | 1.68 | 0.10 | >.999 | 0.93 | 0.36 | >.999 |
| Right Superior Temporal Gyrus & Subcallosal Gyrus-Entorhinal Area | Left Inferior Frontal and Extra Nuclear Gyrus of the Pre-Frontal Lobes | 1.54 | 0.13 | >.999 | 1.61 | 0.11 | >.999 | 2.43 | 0.02 | >.999 | 2.07 | 0.04 | >.999 | 1.12 | 0.27 | >.999 |
| Right Superior Temporal Gyrus & Subcallosal Gyrus-Entorhinal Area | Right Inferior Frontal and Extra Nuclear Gyrus of the Pre-Frontal Lobes | 1.15 | 0.25 | >.999 | 1.50 | 0.14 | >.999 | 1.83 | 0.07 | >.999 | 0.63 | 0.53 | >.999 | 0.21 | 0.84 | >.999 |
| Right Superior Temporal Gyrus & Subcallosal Gyrus-Entorhinal Area | Left Inferior Frontal and Extra Nuclear Gyrus of the Pre-Frontal Lobes | 2.25 | 0.03 | >.999 | 2.52 | 0.01 | >.999 | 2.43 | 0.02 | >.999 | 2.89 | <.001 | >.999 | 1.87 | 0.06 | >.999 |
| Right Superior Temporal Gyrus & Subcallosal Gyrus-Entorhinal Area | Right Inferior Frontal and Extra Nuclear Gyrus of the Pre-Frontal Lobes | 0.87 | 0.39 | >.999 | 1.61 | 0.11 | >.999 | 2.21 | 0.03 | >.999 | 0.82 | 0.41 | >.999 | 0.28 | 0.78 | >.999 |
| Right Superior Temporal Gyrus & Subcallosal Gyrus-Entorhinal Area | Left Middle Frontal Gyrus | 2.23 | 0.03 | >.999 | 2.93 | <.001 | >.999 | 3.15 | <.001 | 0.71 | 2.72 | 0.01 | >.999 | 1.32 | 0.19 | >.999 |
| Right Superior Temporal Gyrus & Subcallosal Gyrus-Entorhinal Area | Right Middle Frontal Gyrus | -0.21 | 0.83 | >.999 | 0.25 | 0.80 | >.999 | 1.08 | 0.28 | >.999 | -1.03 | 0.31 | >.999 | -0.75 | 0.45 | >.999 |
| Right Superior Temporal Gyrus & Subcallosal Gyrus-Entorhinal Area | Left Inferior Frontal | 2.37 | 0.02 | >.999 | 2.45 | 0.02 | >.999 | 2.25 | 0.03 | >.999 | 1.79 | 0.08 | >.999 | 0.91 | 0.36 | >.999 |
| Right Superior Temporal Gyrus & Subcallosal Gyrus-Entorhinal Area | Right Inferior Frontal | 1.21 | 0.23 | >.999 | 2.25 | 0.03 | >.999 | 2.64 | 0.01 | >.999 | 1.38 | 0.17 | >.999 | 0.81 | 0.42 | >.999 |
| Right Superior Temporal Gyrus & Subcallosal Gyrus-Entorhinal Area | Left Parasubiculum hippocampal region | 2.72 | 0.01 | >.999 | 2.89 | <.001 | >.999 | 2.71 | 0.01 | >.999 | 2.69 | 0.01 | >.999 | 2.57 | 0.01 | >.999 |
| Right Superior Temporal Gyrus & Subcallosal Gyrus-Entorhinal Area | Right Parasubiculum hippocampal region | 3.60 | <.001 | 0.17 | 3.68 | <.001 | 0.13 | 2.74 | 0.01 | >.999 | 2.81 | 0.01 | >.999 | 2.13 | 0.03 | >.999 |
| Left Inferior Frontal and Extra Nuclear Gyrus of the Pre-Frontal Lobes | Left Insula | 0.57 | 0.57 | >.999 | 0.76 | 0.45 | >.999 | 2.04 | 0.04 | >.999 | -0.10 | 0.92 | >.999 | -1.44 | 0.15 | >.999 |
| Left Inferior Frontal and Extra Nuclear Gyrus of the Pre-Frontal Lobes | Right Insula | 1.40 | 0.16 | >.999 | 1.80 | 0.07 | >.999 | 2.84 | 0.01 | >.999 | 2.58 | 0.01 | >.999 | 1.41 | 0.16 | >.999 |
| Left Inferior Frontal and Extra Nuclear Gyrus of the Pre-Frontal Lobes | Right Inferior Frontal and Extra Nuclear Gyrus of the Pre-Frontal Lobes | 2.01 | 0.05 | >.999 | 1.65 | 0.10 | >.999 | 2.74 | 0.01 | >.999 | 1.65 | 0.10 | >.999 | 0.99 | 0.32 | >.999 |
| Left Inferior Frontal and Extra Nuclear Gyrus of the Pre-Frontal Lobes | Left Inferior Frontal and Extra Nuclear Gyrus of the Pre-Frontal Lobes | 1.50 | 0.13 | >.999 | 1.84 | 0.07 | >.999 | 3.01 | <.001 | >.999 | 1.77 | 0.08 | >.999 | 1.47 | 0.14 | >.999 |
| Left Inferior Frontal and Extra Nuclear Gyrus of the Pre-Frontal Lobes | Right Inferior Frontal and Extra Nuclear Gyrus of the Pre-Frontal Lobes | 1.42 | 0.16 | >.999 | 1.47 | 0.14 | >.999 | 2.65 | 0.01 | >.999 | 1.69 | 0.09 | >.999 | 0.33 | 0.74 | >.999 |
| Left Inferior Frontal and Extra Nuclear Gyrus of the Pre-Frontal Lobes | Left Middle Frontal Gyrus | 1.74 | 0.08 | >.999 | 2.13 | 0.03 | >.999 | 3.33 | <.001 | 0.42 | 2.06 | 0.04 | >.999 | 2.03 | 0.04 | >.999 |
| Left Inferior Frontal and Extra Nuclear Gyrus of the Pre-Frontal Lobes | Right Middle Frontal Gyrus | 1.09 | 0.28 | >.999 | 1.01 | 0.31 | >.999 | 2.48 | 0.01 | >.999 | >.999 | 0.32 | >.999 | 0.48 | 0.63 | >.999 |
| Left Inferior Frontal and Extra Nuclear Gyrus of the Pre-Frontal Lobes | Left Inferior Frontal | 1.65 | 0.10 | >.999 | 1.93 | 0.05 | >.999 | 2.80 | 0.01 | >.999 | 0.88 | 0.38 | >.999 | -0.54 | 0.59 | >.999 |
| Left Inferior Frontal and Extra Nuclear Gyrus of the Pre-Frontal Lobes | Right Inferior Frontal | 1.49 | 0.14 | >.999 | 1.75 | 0.08 | >.999 | 2.88 | <.001 | >.999 | 2.78 | 0.01 | >.999 | 1.33 | 0.18 | >.999 |
| Left Inferior Frontal and Extra Nuclear Gyrus of the Pre-Frontal Lobes | Left Parasubiculum hippocampal region | 0.41 | 0.68 | >.999 | 0.34 | 0.73 | >.999 | 1.43 | 0.15 | >.999 | -0.71 | 0.48 | >.999 | -1.45 | 0.15 | >.999 |
| Left Inferior Frontal and Extra Nuclear Gyrus of the Pre-Frontal Lobes | Right Parasubiculum hippocampal region | 0.95 | 0.34 | >.999 | 0.98 | 0.33 | >.999 | 1.99 | 0.05 | >.999 | 0.91 | 0.36 | >.999 | 0.73 | 0.47 | >.999 |
| Right Inferior Frontal and Extra Nuclear Gyrus of the Pre-Frontal Lobes | Left Insula | 1.94 | 0.05 | >.999 | 1.70 | 0.09 | >.999 | 2.42 | 0.02 | >.999 | 1.96 | 0.05 | >.999 | 1.42 | 0.16 | >.999 |
| Right Inferior Frontal and Extra Nuclear Gyrus of the Pre-Frontal Lobes | Right Insula | 0.67 | 0.50 | >.999 | 1.05 | 0.30 | >.999 | 1.14 | 0.26 | >.999 | 0.63 | 0.53 | >.999 | 0.13 | 0.90 | >.999 |
| Right Inferior Frontal and Extra Nuclear Gyrus of the Pre-Frontal Lobes | Left Inferior Frontal and Extra Nuclear Gyrus of the Pre-Frontal Lobes | 1.65 | 0.10 | >.999 | 1.49 | 0.14 | >.999 | 2.22 | 0.03 | >.999 | 2.21 | 0.03 | >.999 | 1.27 | 0.21 | >.999 |
| Right Inferior Frontal and Extra Nuclear Gyrus of the Pre-Frontal Lobes | Right Inferior Frontal and Extra Nuclear Gyrus of the Pre-Frontal Lobes | 2.50 | 0.01 | >.999 | 2.85 | 0.01 | >.999 | 2.89 | <.001 | >.999 | 1.87 | 0.06 | >.999 | 1.05 | 0.29 | >.999 |
| Right Inferior Frontal and Extra Nuclear Gyrus of the Pre-Frontal Lobes | Left Middle Frontal Gyrus | 1.70 | 0.09 | >.999 | 2.07 | 0.04 | >.999 | 2.77 | 0.01 | >.999 | 1.83 | 0.07 | >.999 | 0.50 | 0.62 | >.999 |
| Right Inferior Frontal and Extra Nuclear Gyrus of the Pre-Frontal Lobes | Right Middle Frontal Gyrus | 1.90 | 0.06 | >.999 | 1.67 | 0.10 | >.999 | 2.24 | 0.03 | >.999 | 1.09 | 0.28 | >.999 | 1.36 | 0.18 | >.999 |
| Right Inferior Frontal and Extra Nuclear Gyrus of the Pre-Frontal Lobes | Left Inferior Frontal | 2.24 | 0.03 | >.999 | 1.83 | 0.07 | >.999 | 2.44 | 0.02 | >.999 | 2.08 | 0.04 | >.999 | 1.28 | 0.20 | >.999 |
| Right Inferior Frontal and Extra Nuclear Gyrus of the Pre-Frontal Lobes | Right Inferior Frontal | 1.88 | 0.06 | >.999 | 2.12 | 0.04 | >.999 | 2.84 | 0.01 | >.999 | 2.22 | 0.03 | >.999 | 1.74 | 0.08 | >.999 |
| Right Inferior Frontal and Extra Nuclear Gyrus of the Pre-Frontal Lobes | Left Parasubiculum hippocampal region | 1.34 | 0.18 | >.999 | 1.28 | 0.20 | >.999 | 1.85 | 0.07 | >.999 | 1.03 | 0.30 | >.999 | 1.15 | 0.25 | >.999 |
| Right Inferior Frontal and Extra Nuclear Gyrus of the Pre-Frontal Lobes | Right Parasubiculum hippocampal region | 0.86 | 0.39 | >.999 | 1.29 | 0.20 | >.999 | 1.53 | 0.13 | >.999 | 0.49 | 0.63 | >.999 | 0.42 | 0.67 | >.999 |
| Left Inferior Frontal and Extra Nuclear Gyrus of the Pre-Frontal Lobes | Left Insula | -0.23 | 0.82 | >.999 | 0.25 | 0.80 | >.999 | 0.86 | 0.39 | >.999 | 0.10 | 0.92 | >.999 | -0.64 | 0.53 | >.999 |
| Left Inferior Frontal and Extra Nuclear Gyrus of the Pre-Frontal Lobes | Right Insula | 2.32 | 0.02 | >.999 | 2.48 | 0.01 | >.999 | 2.68 | 0.01 | >.999 | 3.00 | <.001 | >.999 | 2.07 | 0.04 | >.999 |
| Left Inferior Frontal and Extra Nuclear Gyrus of the Pre-Frontal Lobes | Right Inferior Frontal and Extra Nuclear Gyrus of the Pre-Frontal Lobes | 2.04 | 0.04 | >.999 | 1.96 | 0.05 | >.999 | 2.41 | 0.02 | >.999 | 2.56 | 0.01 | >.999 | 1.30 | 0.19 | >.999 |
| Left Inferior Frontal and Extra Nuclear Gyrus of the Pre-Frontal Lobes | Left Middle Frontal Gyrus | -0.08 | 0.94 | >.999 | 0.67 | 0.50 | >.999 | 1.88 | 0.06 | >.999 | 0.51 | 0.61 | >.999 | 0.28 | 0.78 | >.999 |
| Left Inferior Frontal and Extra Nuclear Gyrus of the Pre-Frontal Lobes | Right Middle Frontal Gyrus | 1.51 | 0.13 | >.999 | 1.77 | 0.08 | >.999 | 2.32 | 0.02 | >.999 | 1.44 | 0.15 | >.999 | 0.49 | 0.62 | >.999 |
| Left Inferior Frontal and Extra Nuclear Gyrus of the Pre-Frontal Lobes | Left Inferior Frontal | -0.61 | 0.54 | >.999 | -0.19 | 0.85 | >.999 | 0.45 | 0.65 | >.999 | -0.11 | 0.91 | >.999 | -0.70 | 0.49 | >.999 |
| Left Inferior Frontal and Extra Nuclear Gyrus of the Pre-Frontal Lobes | Right Inferior Frontal | 2.43 | 0.02 | >.999 | 2.65 | 0.01 | >.999 | 2.81 | 0.01 | >.999 | 3.08 | <.001 | 0.90 | 2.13 | 0.03 | >.999 |
| Left Inferior Frontal and Extra Nuclear Gyrus of the Pre-Frontal Lobes | Left Parasubiculum hippocampal region | 2.30 | 0.02 | >.999 | 2.50 | 0.01 | >.999 | 2.66 | 0.01 | >.999 | 1.50 | 0.14 | >.999 | 0.76 | 0.45 | >.999 |
| Left Inferior Frontal and Extra Nuclear Gyrus of the Pre-Frontal Lobes | Right Parasubiculum hippocampal region | 2.51 | 0.01 | >.999 | 2.70 | 0.01 | >.999 | 2.63 | 0.01 | >.999 | 2.42 | 0.02 | >.999 | 1.47 | 0.14 | >.999 |
| Right Inferior Frontal and Extra Nuclear Gyrus of the Pre-Frontal Lobes | Left Insula | 1.97 | 0.05 | >.999 | 1.80 | 0.07 | >.999 | 2.48 | 0.01 | >.999 | 2.62 | 0.01 | >.999 | 1.96 | 0.05 | >.999 |
| Right Inferior Frontal and Extra Nuclear Gyrus of the Pre-Frontal Lobes | Right Insula | -0.94 | 0.35 | >.999 | -0.19 | 0.85 | >.999 | 0.12 | 0.90 | >.999 | -1.08 | 0.28 | >.999 | -1.09 | 0.28 | >.999 |
| Right Inferior Frontal and Extra Nuclear Gyrus of the Pre-Frontal Lobes | Left Middle Frontal Gyrus | 1.94 | 0.05 | >.999 | 2.23 | 0.03 | >.999 | 2.78 | 0.01 | >.999 | 1.35 | 0.18 | >.999 | -0.35 | 0.73 | >.999 |
| Right Inferior Frontal and Extra Nuclear Gyrus of the Pre-Frontal Lobes | Right Middle Frontal Gyrus | -1.24 | 0.22 | >.999 | -0.88 | 0.38 | >.999 | -0.59 | 0.56 | >.999 | -2.59 | 0.01 | >.999 | -2.49 | 0.01 | >.999 |
| Right Inferior Frontal and Extra Nuclear Gyrus of the Pre-Frontal Lobes | Left Inferior Frontal | 2.49 | 0.01 | >.999 | 2.15 | 0.03 | >.999 | 2.60 | 0.01 | >.999 | 2.57 | 0.01 | >.999 | 1.84 | 0.07 | >.999 |
| Right Inferior Frontal and Extra Nuclear Gyrus of the Pre-Frontal Lobes | Right Inferior Frontal | -0.04 | 0.97 | >.999 | 0.50 | 0.62 | >.999 | 0.93 | 0.35 | >.999 | 0.38 | 0.70 | >.999 | -0.02 | 0.99 | >.999 |
| Right Inferior Frontal and Extra Nuclear Gyrus of the Pre-Frontal Lobes | Left Parasubiculum hippocampal region | 1.63 | 0.11 | >.999 | 1.47 | 0.14 | >.999 | 1.94 | 0.05 | >.999 | 2.41 | 0.02 | >.999 | 2.35 | 0.02 | >.999 |
| Right Inferior Frontal and Extra Nuclear Gyrus of the Pre-Frontal Lobes | Right Parasubiculum hippocampal region | 1.89 | 0.06 | >.999 | 2.05 | 0.04 | >.999 | 2.18 | 0.03 | >.999 | 1.94 | 0.05 | >.999 | 1.44 | 0.15 | >.999 |
| Left Middle Frontal Gyrus | Left Insula | 0.43 | 0.66 | >.999 | 1.05 | 0.29 | >.999 | 2.07 | 0.04 | >.999 | 0.51 | 0.61 | >.999 | -0.32 | 0.75 | >.999 |
| Left Middle Frontal Gyrus | Right Insula | 2.14 | 0.03 | >.999 | 2.71 | 0.01 | >.999 | 3.12 | <.001 | 0.78 | 2.49 | 0.01 | >.999 | 1.12 | 0.26 | >.999 |
| Left Middle Frontal Gyrus | Right Middle Frontal Gyrus | 1.25 | 0.21 | >.999 | 1.96 | 0.05 | >.999 | 2.73 | 0.01 | >.999 | <.001 | >.999 | >.999 | -0.56 | 0.57 | >.999 |
| Left Middle Frontal Gyrus | Left Inferior Frontal | -0.37 | 0.71 | >.999 | 0.45 | 0.65 | >.999 | 1.16 | 0.25 | >.999 | -0.75 | 0.45 | >.999 | -1.57 | 0.12 | >.999 |
| Left Middle Frontal Gyrus | Right Inferior Frontal | 2.19 | 0.03 | >.999 | 2.82 | 0.01 | >.999 | 3.28 | <.001 | 0.49 | 2.47 | 0.01 | >.999 | 1.22 | 0.22 | >.999 |
| Left Middle Frontal Gyrus | Left Parasubiculum hippocampal region | 2.26 | 0.03 | >.999 | 2.71 | 0.01 | >.999 | 3.33 | <.001 | 0.42 | 1.92 | 0.06 | >.999 | 0.97 | 0.33 | >.999 |
| Left Middle Frontal Gyrus | Right Parasubiculum hippocampal region | 2.47 | 0.01 | >.999 | 3.03 | <.001 | >.999 | 3.48 | <.001 | 0.26 | 2.75 | 0.01 | >.999 | 1.40 | 0.16 | >.999 |
| Right Middle Frontal Gyrus | Left Insula | 1.35 | 0.18 | >.999 | 1.25 | 0.21 | >.999 | 2.03 | 0.04 | >.999 | 1.48 | 0.14 | >.999 | 0.75 | 0.45 | >.999 |
| Right Middle Frontal Gyrus | Right Insula | -1.34 | 0.18 | >.999 | -0.94 | 0.35 | >.999 | -0.62 | 0.54 | >.999 | -2.94 | <.001 | >.999 | -3.13 | <.001 | 0.80 |
| Right Middle Frontal Gyrus | Left Inferior Frontal | 1.93 | 0.05 | >.999 | 1.83 | 0.07 | >.999 | 2.22 | 0.03 | >.999 | 1.40 | 0.16 | >.999 | 0.53 | 0.60 | >.999 |
| Right Middle Frontal Gyrus | Right Inferior Frontal | -1.71 | 0.09 | >.999 | -1.33 | 0.18 | >.999 | -1.08 | 0.28 | >.999 | -2.36 | 0.02 | >.999 | -2.38 | 0.02 | >.999 |
| Right Middle Frontal Gyrus | Left Parasubiculum hippocampal region | 0.75 | 0.45 | >.999 | 0.50 | 0.62 | >.999 | 1.36 | 0.18 | >.999 | 1.31 | 0.19 | >.999 | 1.36 | 0.18 | >.999 |
| Right Middle Frontal Gyrus | Right Parasubiculum hippocampal region | 0.61 | 0.54 | >.999 | 0.75 | 0.45 | >.999 | 1.41 | 0.16 | >.999 | 0.46 | 0.64 | >.999 | 0.48 | 0.63 | >.999 |
| Left Inferior Frontal | Left Insula | 1.22 | 0.22 | >.999 | 1.19 | 0.23 | >.999 | 1.61 | 0.11 | >.999 | 1.02 | 0.31 | >.999 | 0.26 | 0.80 | >.999 |
| Left Inferior Frontal | Right Insula | 2.79 | 0.01 | >.999 | 2.64 | 0.01 | >.999 | 2.84 | 0.01 | >.999 | 2.91 | <.001 | >.999 | 2.59 | 0.01 | >.999 |
| Left Inferior Frontal | Right Inferior Frontal | 2.73 | 0.01 | >.999 | 2.65 | 0.01 | >.999 | 2.85 | <.001 | >.999 | 3.00 | <.001 | >.999 | 2.91 | <.001 | >.999 |
| Left Inferior Frontal | Left Parasubiculum hippocampal region | 2.96 | <.001 | >.999 | 2.76 | 0.01 | >.999 | 3.00 | <.001 | >.999 | 1.56 | 0.12 | >.999 | 0.36 | 0.72 | >.999 |
| Left Inferior Frontal | Right Parasubiculum hippocampal region | 2.91 | <.001 | >.999 | 2.84 | 0.01 | >.999 | 2.79 | 0.01 | >.999 | 1.84 | 0.07 | >.999 | 0.65 | 0.51 | >.999 |
| Right Inferior Frontal | Left Insula | 2.30 | 0.02 | >.999 | 2.23 | 0.03 | >.999 | 2.75 | 0.01 | >.999 | 2.75 | 0.01 | >.999 | 2.34 | 0.02 | >.999 |
| Right Inferior Frontal | Right Insula | 1.10 | 0.27 | >.999 | 1.80 | 0.07 | >.999 | 2.84 | 0.01 | >.999 | 1.28 | 0.20 | >.999 | 0.59 | 0.56 | >.999 |
| Right Inferior Frontal | Left Parasubiculum hippocampal region | 2.86 | <.001 | >.999 | 3.21 | <.001 | 0.60 | 3.01 | <.001 | >.999 | 3.59 | <.001 | 0.17 | 3.26 | <.001 | 0.54 |
| Right Inferior Frontal | Right Parasubiculum hippocampal region | 3.07 | <.001 | 0.98 | 3.60 | <.001 | 0.17 | 3.15 | <.001 | 0.72 | 2.87 | <.001 | >.999 | 2.03 | 0.04 | >.999 |
| Left Parasubiculum hippocampal region | Right Parasubiculum hippocampal region | 1.55 | 0.12 | >.999 | 0.82 | 0.41 | >.999 | 1.27 | 0.21 | >.999 | 1.93 | 0.06 | >.999 | 2.08 | 0.04 | >.999 |
| Left Pre-Frontal Cortex | Left Pre-Frontal Cortex | 2.29 | 0.02 | >.999 | 2.43 | 0.02 | >.999 | 3.37 | <.001 | 0.36 | 1.67 | 0.10 | >.999 | 0.81 | 0.42 | >.999 |
| Left Pre-Frontal Cortex | Right Pre-Frontal Cortex | 1.63 | 0.10 | >.999 | 1.41 | 0.16 | >.999 | 2.36 | 0.02 | >.999 | 1.55 | 0.12 | >.999 | 1.47 | 0.14 | >.999 |
| Left Pre-Frontal Cortex | Left Insula | 0.07 | 0.94 | >.999 | 0.42 | 0.67 | >.999 | 1.86 | 0.07 | >.999 | -0.32 | 0.75 | >.999 | -0.85 | 0.40 | >.999 |
| Left Pre-Frontal Cortex | Right Insula | 1.65 | 0.10 | >.999 | 1.54 | 0.13 | >.999 | 3.05 | <.001 | 0.96 | 2.07 | 0.04 | >.999 | 1.71 | 0.09 | >.999 |
| Left Pre-Frontal Cortex | Left Temporal Lobe | -0.08 | 0.94 | >.999 | 0.70 | 0.49 | >.999 | 2.21 | 0.03 | >.999 | 1.28 | 0.20 | >.999 | 0.66 | 0.51 | >.999 |
| Left Pre-Frontal Cortex | Right Temporal Lobe | 0.89 | 0.37 | >.999 | 1.04 | 0.30 | >.999 | 2.80 | 0.01 | >.999 | 1.75 | 0.08 | >.999 | 0.76 | 0.45 | >.999 |
| Left Pre-Frontal Cortex | Left Posterior Cingulate Gyrus | 0.81 | 0.42 | >.999 | 0.05 | 0.96 | >.999 | 1.27 | 0.20 | >.999 | 0.18 | 0.85 | >.999 | 0.40 | 0.69 | >.999 |
| Left Pre-Frontal Cortex | Right Posterior Cingulate Gyrus | 0.99 | 0.32 | >.999 | 0.18 | 0.86 | >.999 | 1.51 | 0.13 | >.999 | 0.76 | 0.45 | >.999 | 1.16 | 0.25 | >.999 |
| Left Pre-Frontal Cortex | Left Anterior Cingulate Gyrus | -2.88 | <.001 | >.999 | -2.56 | 0.01 | >.999 | -2.55 | 0.01 | >.999 | -2.88 | <.001 | >.999 | -2.79 | 0.01 | >.999 |
| Left Pre-Frontal Cortex | Right Anterior Cingulate Gyrus | -2.26 | 0.03 | >.999 | -2.40 | 0.02 | >.999 | -2.36 | 0.02 | >.999 | -2.63 | 0.01 | >.999 | -2.64 | 0.01 | >.999 |
| Left Pre-Frontal Cortex | Left Anterior Cingulate Gyrus | 0.53 | 0.60 | >.999 | 0.70 | 0.49 | >.999 | 2.13 | 0.03 | >.999 | 0.36 | 0.72 | >.999 | -0.20 | 0.84 | >.999 |
| Left Pre-Frontal Cortex | Right Anterior Cingulate Gyrus | 0.84 | 0.40 | >.999 | 0.85 | 0.40 | >.999 | 2.26 | 0.03 | >.999 | 0.81 | 0.42 | >.999 | 0.37 | 0.71 | >.999 |
| Left Pre-Frontal Cortex | Left Posterior Cingulate & Superior Transverse Temporal Gyrus | 0.84 | 0.40 | >.999 | 0.11 | 0.91 | >.999 | 1.52 | 0.13 | >.999 | 0.70 | 0.48 | >.999 | 0.10 | 0.92 | >.999 |
| Left Pre-Frontal Cortex | Right Posterior Cingulate & Superior Transverse Temporal Gyrus | 1.22 | 0.23 | >.999 | -0.06 | 0.95 | >.999 | 1.56 | 0.12 | >.999 | 1.50 | 0.13 | >.999 | 1.28 | 0.20 | >.999 |
| Left Pre-Frontal Cortex | Left Posterior Cingulate & Cuneus | 1.22 | 0.23 | >.999 | 0.94 | 0.35 | >.999 | 2.28 | 0.02 | >.999 | 1.25 | 0.21 | >.999 | -0.13 | 0.89 | >.999 |
| Left Pre-Frontal Cortex | Right Posterior Cingulate & Cuneus | 1.60 | 0.11 | >.999 | 0.30 | 0.76 | >.999 | 1.93 | 0.05 | >.999 | 2.38 | 0.02 | >.999 | 1.32 | 0.19 | >.999 |
| Left Pre-Frontal Cortex | Left Anterior Cingulate Gyrus | -0.13 | 0.89 | >.999 | 0.12 | 0.91 | >.999 | 0.45 | 0.65 | >.999 | -0.14 | 0.89 | >.999 | -0.39 | 0.70 | >.999 |
| Left Pre-Frontal Cortex | Right Anterior Cingulate Gyrus | -0.65 | 0.52 | >.999 | -0.69 | 0.49 | >.999 | 0.15 | 0.88 | >.999 | -0.36 | 0.72 | >.999 | -0.53 | 0.60 | >.999 |
| Left Pre-Frontal Cortex | Right Pre-Frontal Cortex | -1.09 | 0.28 | >.999 | -1.27 | 0.21 | >.999 | -0.92 | 0.36 | >.999 | -1.14 | 0.26 | >.999 | -0.40 | 0.69 | >.999 |
| Left Pre-Frontal Cortex | Left Pre-Frontal Cortex | -0.65 | 0.52 | >.999 | -0.49 | 0.62 | >.999 | -0.47 | 0.64 | >.999 | 0.25 | 0.80 | >.999 | 0.32 | 0.75 | >.999 |
| Left Pre-Frontal Cortex | Right Pre-Frontal Cortex | -0.68 | 0.50 | >.999 | 0.03 | 0.97 | >.999 | 1.20 | 0.23 | >.999 | 0.01 | 0.99 | >.999 | 0.01 | 0.99 | >.999 |
| Right Pre-Frontal Cortex | Left Pre-Frontal Cortex | 1.72 | 0.09 | >.999 | 2.15 | 0.03 | >.999 | 2.45 | 0.02 | >.999 | -0.02 | 0.99 | >.999 | -0.10 | 0.92 | >.999 |
| Right Pre-Frontal Cortex | Right Pre-Frontal Cortex | 2.20 | 0.03 | >.999 | 2.08 | 0.04 | >.999 | 2.18 | 0.03 | >.999 | 0.52 | 0.61 | >.999 | 0.31 | 0.76 | >.999 |
| Right Pre-Frontal Cortex | Left Insula | 2.14 | 0.03 | >.999 | 1.68 | 0.10 | >.999 | 2.56 | 0.01 | >.999 | 1.60 | 0.11 | >.999 | 1.67 | 0.10 | >.999 |
| Right Pre-Frontal Cortex | Right Insula | 0.49 | 0.63 | >.999 | 0.73 | 0.47 | >.999 | 0.99 | 0.32 | >.999 | -1.00 | 0.32 | >.999 | -1.18 | 0.24 | >.999 |
| Right Pre-Frontal Cortex | Left Temporal Lobe | 1.45 | 0.15 | >.999 | 1.14 | 0.26 | >.999 | 2.06 | 0.04 | >.999 | 1.34 | 0.18 | >.999 | 0.34 | 0.73 | >.999 |
| Right Pre-Frontal Cortex | Right Temporal Lobe | 0.52 | 0.61 | >.999 | 1.04 | 0.30 | >.999 | 2.88 | <.001 | >.999 | 2.02 | 0.04 | >.999 | 0.90 | 0.37 | >.999 |
| Right Pre-Frontal Cortex | Left Posterior Cingulate Gyrus | 0.14 | 0.89 | >.999 | -0.32 | 0.75 | >.999 | 1.47 | 0.14 | >.999 | 0.38 | 0.71 | >.999 | 0.87 | 0.38 | >.999 |
| Right Pre-Frontal Cortex | Right Posterior Cingulate Gyrus | -0.06 | 0.95 | >.999 | -0.30 | 0.76 | >.999 | 1.57 | 0.12 | >.999 | 0.18 | 0.86 | >.999 | 0.81 | 0.42 | >.999 |
| Right Pre-Frontal Cortex | Left Anterior Cingulate Gyrus | -1.39 | 0.17 | >.999 | -1.68 | 0.09 | >.999 | -2.48 | 0.01 | >.999 | -1.41 | 0.16 | >.999 | -0.26 | 0.79 | >.999 |
| Right Pre-Frontal Cortex | Right Anterior Cingulate Gyrus | -1.91 | 0.06 | >.999 | -2.04 | 0.04 | >.999 | -3.20 | <.001 | 0.62 | -2.09 | 0.04 | >.999 | -0.97 | 0.33 | >.999 |
| Right Pre-Frontal Cortex | Left Anterior Cingulate Gyrus | 1.53 | 0.13 | >.999 | 1.13 | 0.26 | >.999 | 1.89 | 0.06 | >.999 | 0.93 | 0.36 | >.999 | 1.29 | 0.20 | >.999 |
| Right Pre-Frontal Cortex | Right Anterior Cingulate Gyrus | 1.14 | 0.26 | >.999 | 0.87 | 0.38 | >.999 | 1.56 | 0.12 | >.999 | 0.53 | 0.60 | >.999 | 1.06 | 0.29 | >.999 |
| Right Pre-Frontal Cortex | Left Posterior Cingulate & Superior Transverse Temporal Gyrus | 0.57 | 0.57 | >.999 | -0.37 | 0.71 | >.999 | 1.47 | 0.14 | >.999 | 1.00 | 0.32 | >.999 | 1.12 | 0.27 | >.999 |
| Right Pre-Frontal Cortex | Right Posterior Cingulate & Superior Transverse Temporal Gyrus | 0.37 | 0.71 | >.999 | -0.02 | 0.99 | >.999 | 1.99 | 0.05 | >.999 | 0.62 | 0.54 | >.999 | 0.75 | 0.45 | >.999 |
| Right Pre-Frontal Cortex | Left Posterior Cingulate & Cuneus | 0.33 | 0.74 | >.999 | -0.38 | 0.71 | >.999 | 1.37 | 0.17 | >.999 | 0.88 | 0.38 | >.999 | 0.91 | 0.36 | >.999 |
| Right Pre-Frontal Cortex | Right Posterior Cingulate & Cuneus | 0.50 | 0.62 | >.999 | <.001 | >.999 | >.999 | 1.93 | 0.06 | >.999 | 0.83 | 0.41 | >.999 | 0.59 | 0.56 | >.999 |
| Right Pre-Frontal Cortex | Left Anterior Cingulate Gyrus | 0.04 | 0.96 | >.999 | -0.11 | 0.92 | >.999 | -0.21 | 0.84 | >.999 | -0.06 | 0.95 | >.999 | 0.26 | 0.80 | >.999 |
| Right Pre-Frontal Cortex | Right Anterior Cingulate Gyrus | 0.65 | 0.52 | >.999 | 0.13 | 0.90 | >.999 | -0.02 | 0.99 | >.999 | 0.38 | 0.70 | >.999 | 0.64 | 0.52 | >.999 |
| Right Pre-Frontal Cortex | Left Pre-Frontal Cortex | 0.23 | 0.82 | >.999 | 0.46 | 0.65 | >.999 | 1.33 | 0.18 | >.999 | 0.76 | 0.45 | >.999 | 1.35 | 0.18 | >.999 |
| Right Pre-Frontal Cortex | Right Pre-Frontal Cortex | 0.62 | 0.53 | >.999 | 0.19 | 0.85 | >.999 | -0.31 | 0.76 | >.999 | -0.11 | 0.91 | >.999 | 0.32 | 0.75 | >.999 |
| Left Pre-Frontal Cortex | Left Pre-Frontal Cortex | 1.39 | 0.17 | >.999 | 1.97 | 0.05 | >.999 | 2.62 | 0.01 | >.999 | 0.67 | 0.50 | >.999 | -0.38 | 0.71 | >.999 |
| Left Pre-Frontal Cortex | Right Pre-Frontal Cortex | 1.01 | 0.32 | >.999 | 1.70 | 0.09 | >.999 | 2.70 | 0.01 | >.999 | 2.09 | 0.04 | >.999 | 2.06 | 0.04 | >.999 |
| Left Pre-Frontal Cortex | Left Insula | -0.31 | 0.76 | >.999 | 0.27 | 0.79 | >.999 | 1.38 | 0.17 | >.999 | -0.49 | 0.62 | >.999 | -1.17 | 0.24 | >.999 |
| Left Pre-Frontal Cortex | Right Insula | 1.74 | 0.08 | >.999 | 2.10 | 0.04 | >.999 | 3.18 | <.001 | 0.66 | 2.87 | <.001 | >.999 | 1.84 | 0.07 | >.999 |
| Left Pre-Frontal Cortex | Left Temporal Lobe | 1.86 | 0.06 | >.999 | 1.90 | 0.06 | >.999 | 2.57 | 0.01 | >.999 | 2.42 | 0.02 | >.999 | 1.70 | 0.09 | >.999 |
| Left Pre-Frontal Cortex | Right Temporal Lobe | 0.82 | 0.41 | >.999 | 0.92 | 0.36 | >.999 | 2.16 | 0.03 | >.999 | 1.12 | 0.27 | >.999 | -0.18 | 0.86 | >.999 |
| Left Pre-Frontal Cortex | Left Posterior Cingulate Gyrus | 1.68 | 0.10 | >.999 | 1.20 | 0.23 | >.999 | 1.82 | 0.07 | >.999 | 0.95 | 0.34 | >.999 | 0.21 | 0.84 | >.999 |
| Left Pre-Frontal Cortex | Right Posterior Cingulate Gyrus | 1.73 | 0.09 | >.999 | 1.31 | 0.19 | >.999 | 1.97 | 0.05 | >.999 | 1.45 | 0.15 | >.999 | 0.73 | 0.46 | >.999 |
| Left Pre-Frontal Cortex | Left Anterior Cingulate Gyrus | -1.89 | 0.06 | >.999 | -1.47 | 0.14 | >.999 | -1.03 | 0.30 | >.999 | -1.70 | 0.09 | >.999 | -2.00 | 0.05 | >.999 |
| Left Pre-Frontal Cortex | Right Anterior Cingulate Gyrus | -1.42 | 0.16 | >.999 | -1.10 | 0.27 | >.999 | -0.37 | 0.71 | >.999 | -0.92 | 0.36 | >.999 | -1.04 | 0.30 | >.999 |
| Left Pre-Frontal Cortex | Left Anterior Cingulate Gyrus | -0.26 | 0.79 | >.999 | 0.17 | 0.87 | >.999 | 1.36 | 0.18 | >.999 | -0.44 | 0.66 | >.999 | -1.09 | 0.28 | >.999 |
| Left Pre-Frontal Cortex | Right Anterior Cingulate Gyrus | 0.25 | 0.80 | >.999 | 0.81 | 0.42 | >.999 | 2.03 | 0.04 | >.999 | 0.77 | 0.44 | >.999 | 0.29 | 0.77 | >.999 |
| Left Pre-Frontal Cortex | Left Posterior Cingulate & Superior Transverse Temporal Gyrus | 1.33 | 0.19 | >.999 | 1.26 | 0.21 | >.999 | 1.80 | 0.07 | >.999 | 0.78 | 0.44 | >.999 | -0.35 | 0.73 | >.999 |
| Left Pre-Frontal Cortex | Right Posterior Cingulate & Superior Transverse Temporal Gyrus | 1.76 | 0.08 | >.999 | 1.07 | 0.28 | >.999 | 2.02 | 0.05 | >.999 | 1.97 | 0.05 | >.999 | 0.83 | 0.41 | >.999 |
| Left Pre-Frontal Cortex | Left Posterior Cingulate & Cuneus | 1.79 | 0.08 | >.999 | 2.32 | 0.02 | >.999 | 2.55 | 0.01 | >.999 | 1.21 | 0.23 | >.999 | -0.07 | 0.95 | >.999 |
| Left Pre-Frontal Cortex | Right Posterior Cingulate & Cuneus | 1.99 | 0.05 | >.999 | 1.33 | 0.19 | >.999 | 2.26 | 0.03 | >.999 | 2.06 | 0.04 | >.999 | 0.51 | 0.61 | >.999 |
| Left Pre-Frontal Cortex | Left Anterior Cingulate Gyrus | -0.80 | 0.43 | >.999 | <.001 | >.999 | >.999 | 0.61 | 0.55 | >.999 | -0.42 | 0.67 | >.999 | -0.83 | 0.41 | >.999 |
| Left Pre-Frontal Cortex | Right Anterior Cingulate Gyrus | -0.89 | 0.38 | >.999 | -0.14 | 0.89 | >.999 | 1.55 | 0.12 | >.999 | 0.60 | 0.55 | >.999 | 0.71 | 0.48 | >.999 |
| Left Pre-Frontal Cortex | Right Pre-Frontal Cortex | 0.50 | 0.62 | >.999 | 1.47 | 0.14 | >.999 | 2.76 | 0.01 | >.999 | 1.96 | 0.05 | >.999 | 1.88 | 0.06 | >.999 |
| Right Pre-Frontal Cortex | Left Pre-Frontal Cortex | 0.95 | 0.34 | >.999 | 1.61 | 0.11 | >.999 | 1.85 | 0.07 | >.999 | 0.09 | 0.93 | >.999 | 0.16 | 0.88 | >.999 |
| Right Pre-Frontal Cortex | Right Pre-Frontal Cortex | 1.31 | 0.19 | >.999 | 1.66 | 0.10 | >.999 | 1.51 | 0.13 | >.999 | 1.06 | 0.29 | >.999 | 1.02 | 0.31 | >.999 |
| Right Pre-Frontal Cortex | Left Insula | 1.16 | 0.25 | >.999 | 1.01 | 0.31 | >.999 | 2.12 | 0.04 | >.999 | 1.10 | 0.28 | >.999 | 0.53 | 0.59 | >.999 |
| Right Pre-Frontal Cortex | Right Insula | -0.63 | 0.53 | >.999 | -0.31 | 0.76 | >.999 | -0.38 | 0.71 | >.999 | -2.23 | 0.03 | >.999 | -2.44 | 0.02 | >.999 |
| Right Pre-Frontal Cortex | Left Temporal Lobe | 1.29 | 0.20 | >.999 | 0.66 | 0.51 | >.999 | 1.36 | 0.18 | >.999 | 1.09 | 0.28 | >.999 | 0.04 | 0.97 | >.999 |
| Right Pre-Frontal Cortex | Right Temporal Lobe | 0.28 | 0.78 | >.999 | 1.13 | 0.26 | >.999 | 2.80 | 0.01 | >.999 | 0.56 | 0.58 | >.999 | -0.39 | 0.70 | >.999 |
| Right Pre-Frontal Cortex | Left Posterior Cingulate Gyrus | 0.35 | 0.72 | >.999 | -0.01 | 0.99 | >.999 | 1.51 | 0.13 | >.999 | 0.95 | 0.34 | >.999 | 0.65 | 0.51 | >.999 |
| Right Pre-Frontal Cortex | Right Posterior Cingulate Gyrus | 0.52 | 0.60 | >.999 | 0.37 | 0.71 | >.999 | 1.85 | 0.07 | >.999 | 0.90 | 0.37 | >.999 | 0.54 | 0.59 | >.999 |
| Right Pre-Frontal Cortex | Left Anterior Cingulate Gyrus | -1.32 | 0.19 | >.999 | -1.00 | 0.32 | >.999 | -1.30 | 0.20 | >.999 | -0.44 | 0.66 | >.999 | 0.22 | 0.82 | >.999 |
| Right Pre-Frontal Cortex | Right Anterior Cingulate Gyrus | -1.66 | 0.10 | >.999 | -1.39 | 0.17 | >.999 | -2.36 | 0.02 | >.999 | -1.25 | 0.21 | >.999 | -0.45 | 0.65 | >.999 |
| Right Pre-Frontal Cortex | Left Anterior Cingulate Gyrus | 0.03 | 0.98 | >.999 | 0.15 | 0.88 | >.999 | 1.06 | 0.29 | >.999 | 0.34 | 0.73 | >.999 | 0.76 | 0.45 | >.999 |
| Right Pre-Frontal Cortex | Right Anterior Cingulate Gyrus | -0.47 | 0.64 | >.999 | -0.29 | 0.78 | >.999 | 0.35 | 0.73 | >.999 | -0.38 | 0.71 | >.999 | 0.15 | 0.88 | >.999 |
| Right Pre-Frontal Cortex | Left Posterior Cingulate & Superior Transverse Temporal Gyrus | 0.37 | 0.71 | >.999 | -0.37 | 0.71 | >.999 | 1.20 | 0.23 | >.999 | 1.16 | 0.25 | >.999 | 0.99 | 0.33 | >.999 |
| Right Pre-Frontal Cortex | Right Posterior Cingulate & Superior Transverse Temporal Gyrus | 0.55 | 0.58 | >.999 | 0.45 | 0.65 | >.999 | 2.03 | 0.04 | >.999 | 0.95 | 0.34 | >.999 | 0.23 | 0.82 | >.999 |
| Right Pre-Frontal Cortex | Left Posterior Cingulate & Cuneus | 0.37 | 0.71 | >.999 | -0.29 | 0.77 | >.999 | 1.08 | 0.28 | >.999 | 0.70 | 0.48 | >.999 | 0.60 | 0.55 | >.999 |
| Right Pre-Frontal Cortex | Right Posterior Cingulate & Cuneus | 0.32 | 0.75 | >.999 | 0.12 | 0.91 | >.999 | 1.75 | 0.08 | >.999 | 0.02 | 0.98 | >.999 | -0.39 | 0.70 | >.999 |
| Right Pre-Frontal Cortex | Left Anterior Cingulate Gyrus | -0.61 | 0.54 | >.999 | 0.12 | 0.90 | >.999 | 0.65 | 0.52 | >.999 | 0.75 | 0.45 | >.999 | 1.03 | 0.31 | >.999 |
| Right Pre-Frontal Cortex | Right Anterior Cingulate Gyrus | -0.42 | 0.67 | >.999 | -0.23 | 0.82 | >.999 | -0.64 | 0.52 | >.999 | 0.99 | 0.32 | >.999 | 1.51 | 0.13 | >.999 |

P_BH_ = The Bonferroni-holm corrected post-hoc comparison was used; *P_BH_ < 0.05.
